# Phylogenetic patterns suggest frequent multiple origins of secondary metabolites across the seed-plant ‘tree of life’

**DOI:** 10.1093/nsr/nwaa105

**Published:** 2020-05-21

**Authors:** Yongzeng Zhang, Tao Deng, Lu Sun, Jacob B Landis, Michael J Moore, Hengchang Wang, Yuehua Wang, Xiaojiang Hao, Jijun Chen, Shenghong Li, Maonian Xu, Pema-Tenzin Puno, Peter H Raven, Hang Sun

**Affiliations:** CAS Key Laboratory for Plant Diversity and Biogeography of East Asia, Kunming Institute of Botany, Chinese Academy of Sciences, Kunming 650201, China; University of the Chinese Academy of Sciences, Beijing 100049, China; CAS Key Laboratory for Plant Diversity and Biogeography of East Asia, Kunming Institute of Botany, Chinese Academy of Sciences, Kunming 650201, China; CAS Key Laboratory for Plant Diversity and Biogeography of East Asia, Kunming Institute of Botany, Chinese Academy of Sciences, Kunming 650201, China; University of the Chinese Academy of Sciences, Beijing 100049, China; Department of Botany and Plant Sciences, University of California Riverside, Riverside, CA 92521, USA; School of Integrative Plant Science, Section of Plant Biology and the L.H. Bailey Hortorium, Cornell University, Ithaca, NY 14853, USA; Department of Biology, Oberlin College, Oberlin, OH 44074, USA; CAS Key Laboratory of Plant Germplasm Enhancement and Specialty Agriculture, Chinese Academy of Sciences, Wuhan 430074, China; School of Life Science, Yunnan University, Kunming 650091, China; State Key Laboratory of Phytochemistry and Plant Resources in West China, Kunming Institute of Botany, Chinese Academy of Sciences, Kunming 650201, China; State Key Laboratory of Phytochemistry and Plant Resources in West China, Kunming Institute of Botany, Chinese Academy of Sciences, Kunming 650201, China; State Key Laboratory of Phytochemistry and Plant Resources in West China, Kunming Institute of Botany, Chinese Academy of Sciences, Kunming 650201, China; Pharmaceutical Sciences, University of Iceland, 107 Reykjavik, Iceland; State Key Laboratory of Phytochemistry and Plant Resources in West China, Kunming Institute of Botany, Chinese Academy of Sciences, Kunming 650201, China; Missouri Botanical Garden, St. Louis, MO 63110, USA; CAS Key Laboratory for Plant Diversity and Biogeography of East Asia, Kunming Institute of Botany, Chinese Academy of Sciences, Kunming 650201, China

**Keywords:** secondary metabolites, phylogenetic tree, phylogenetic signal, co-diversification, evolution, bioprospecting, seed plants

## Abstract

To evaluate the phylogenetic patterns of the distribution and evolution of plant secondary metabolites (PSMs), we selected 8 classes of PSMs and mapped them onto an updated phylogenetic tree including 437 families of seed plants. A significant phylogenetic signal was detected in 17 of the 18 tested seed-plant clades for at least 1 of the 8 PSM classes using the D statistic. The phylogenetic signal, nevertheless, indicated weak clustering of PSMs compared to a random distribution across all seed plants. The observed signal suggests strong diversifying selection during seed-plant evolution and/or relatively weak evolutionary constraints on the evolution of PSMs. In the survey of the current phylogenetic distributions of PSMs, we found that multiple origins of PSM biosynthesis due to external selective forces for diverse genetic pathways may have played important roles. In contrast, a single origin of PSMs seems rather uncommon. The distribution patterns for PSMs observed in this study may also be useful in the search for natural compounds for medicinal purposes.

## INTRODUCTION

Plant secondary metabolites (PSMs) are ubiquitous in plants [1]. They play diverse ecological and physiological roles in defense against herbivores, pathogenic microbes and competing plants [2,3]. They also mediate interactions with pollinators, mycorrhizal fungi and other plants, and confer protection against abiotic stressors such as ultraviolet (UV) radiation, frost and drought [4]. PSMs are important to humans in many ways, representing a major source of medicinal drugs. Many approved drugs, and those currently in clinical trials, are derived from natural products, including 25% being plant-derived [5]. PSMs are characterized by great structural diversity, with >200 000 compounds already identified and many more remaining to be discovered [1].

Despite the importance of PSMs, the patterns and processes of their diversification across seed plants have not yet been thoroughly investigated [2]. The establishment of a clear pattern of phylogenetic diversification is necessary to meaningfully understand the distribution of PSMs [6]. Using comparative phylogenetic approaches and mapping of chemical characteristics, we can identify lineages producing similar PSMs and thus gain an improved understanding of the evolution of secondary metabolic traits. Of practical importance, plants used for the treatment of ailments are often significantly clustered phylogenetically [7]; closely related plants often harbor similar chemical substances and bioactivities [8]. The understanding associated with such findings could assist in the process of engineering biosynthetic pathways to obtain new biologically active and pharmaceutically relevant compounds.

The continuous development of more well-supported plant phylogenies, improved knowledge of plant chemistry and the application of advanced statistical tools and methods have allowed us to resolve some of the patterns in PSM distribution within and between different plant clades. Over the past decade, improvements in DNA sequencing and analytical methods have revolutionized our understanding of seed-plant phylogeny [9]. These advances have allowed us to investigate large-scale patterns of trait evolution in plants, including those involving PSMs, with ever-increasing precision.

A first step in understanding PSM evolution in a phylogenetic context is to test for phylogenetic signals in various PSMs. A phylogenetic signal is the tendency of related species to resemble each other with respect to evolutionarily related traits more than would be expected for species drawn at random from a phylogenetic tree [10], indicating a relationship between the degree of phylogenetic relatedness and phenotypic similarity [11]. Measures of phylogenetic signal are widely employed in ecological and evolutionary studies in many organisms spanning a wide range of traits such as, but not limited to, ecophysiological traits, growth form, habitat and life history [12]. A significant phylogenetic signal can be maintained when closely related species respond to similar ecological pressures, maintaining their adaptive features regardless of whether the traits evolved once in a common ancestor or convergently in the course of evolution. Past studies have often shown the widespread sharing of particular PSMs among closely related plant species [13]. For example, root secondary compounds, especially phenolics, display a significant phylogenetic signal in species of *Eucalyptus* (Myrtaceae) [13]. Studying various members of such groups can often reveal variation in their characteristic PSM groups [14]. Similar investigations of families and higher taxonomic groups have also been fruitful, but comparisons of PSM distribution across entire complete clades are still in their early stages. Large-scale comparisons are of increasing interest as more is learned about the chemical groups, while larger, more inclusive phylogenies become available.

In this study, we investigate the evolution of PSM classes across seed plants at the family level, using an original data set of PSM composition across 437 plant families arrayed according to a robust phylogenetic hypothesis. More specifically, we test (i) the distribution and evolution pattern that PSMs present in the phylogeny of seed plants; (ii) whether the diversity and composition of PSMs display significant a phylogenetic signal and, if so, their evolutionary patterns; (iii) what factors determine the current PSM diversification pattern and (iv) whether PSM diversity and evolution are associated with other biotic evolutionary factors. Finally, we highlight the opportunities to apply approaches that utilize phylogenetic signals for bioprospecting.

## RESULTS

### Numbers of PSMs and their distribution in seed plants

**Figure 1.**
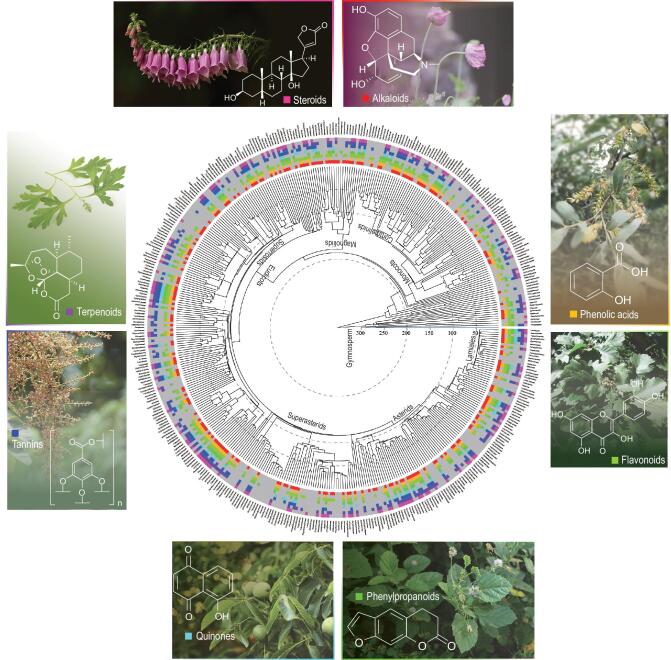
Distribution of major classes of PSMs across seed-plant phylogeny. Each color represents one PSM class. The key to the different colors is provided by the circles surrounding the tree. A gray color indicates absent or missing data.

Our analyses indicate that usable data were available for 309 of the 437 families of angiosperms and gymnosperms (Supplementary Table 2), highlighting the need for more extensive investigations to achieve a comprehensive analysis of the overall PSM distribution for seed plants. Among all PSM classes tested (alkaloids, phenolic acids, flavonoids, tannins, terpenoids, phenylpropanoids, quinones and steroids), a single class was found in 43 families; 41 families had two classes, 36 families had three, 27 families had four, 33 families had five, 39 families had six, 49 families had seven, and 41 families had all eight classes of PSMs (Fig. 1 and Supplementary Table 2). Among gymnosperms (12 families included in this study), relatively few of the PSM classes have been recorded, with only Pinaceae having all eight PSMs investigated. For angiosperms, most early-diverging clades had fewer classes of PSMs than the eudicots or monocots; for example, the earliest-diverging family Nymphaeaceae had three classes of PSMs, whereas the eudicot family Lamiaceae had all eight (Supplementary Table 2).

**Figure 2.**
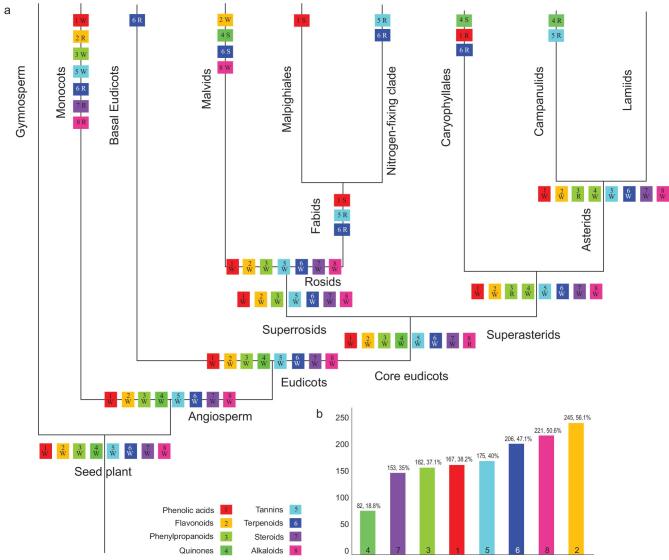
Phylogenetic signal in eight classes of PSMs across seed-plant phylogeny, and PSM class presence among major seed-plant clades. Each color and number represents one PSM class. (a) Phylogenetic signals in PSMs for 17 major clades of seed plants. The presence of a given PSM color and number within a clade indicates a significant phylogenetic signal for that PSM in that clade. R, randomly distributed (D > 1); W, weak clustering signal (0 < D < 1); S, strong clustering signal (D < 0). (b) Histogram depicting the number of seed-plant families with known occurrences of a given PSM class. The percentage of all families tested which contain that class is also indicated.

Among all PSM classes, flavonoids (present in 245 families) were the most widely observed in seed plants, followed by alkaloids (present in 221 families), terpenoids (present in 206 families), tannins (present in 175 families), phenolic acids (present in 167 families), phenylpropanoids (present in 162 families), steroids (present in 153 families) and quinones (present in 82 families) (Fig. 2b).

Alkaloids were most frequent among the families of magnoliids, Ranunculales, Saxifragales, Myrtales, Sapindales, Rosales, Ericales, Gentianales and Lamiales, but were not reported in the earliest-diverging angiosperm lineages (only reported in Nymphaeaceae; Supplementary Table 2 and Supplementary Fig. 1).

Flavonoids were widely distributed in gymnosperms, monocots, Piperales, magnoliids, Proteales, Ranunculales, Sapindales, and fabids (Supplementary Table 2 and Supplementary Fig. 2). Among monocots and eudicots, phenolic acids were best represented in relatively few clades, including commelinids, Saxifragales, Rosales, the nitrogen-fixing clade, Ericales and Lamiales (Fig. 1 and Supplementary Fig. 3).

Phenylpropanoids were widespread in gymnosperms, but less so in angiosperms (Supplementary Table 2 and Supplementary Fig. 4), being frequently reported in only a few clades including Poales, Piperales, Ranunculales, Myrtales and Rosales (Fig. 1 and Supplementary Fig. 4).

Quinones were found only sporadically among members of the families sampled. Among gymnosperms, they were reported only in Pinaceae and, in angiosperms, they were frequent only in Myrtales, Sapindales and Gentianales, with no records from Pandanales, Dioscoreales, Geraniales, Crossosomatales, Brassicales, Oxalidales, Cucurbitales, Santalales or Cornales (Supplementary Table 2, Fig. 1 and Supplementary Fig. 5).

Tannins were relatively widely distributed. Among gymnosperms, they were found in Ginkgoaceae, Ephedraceae, Pinaceae and

Cupressaceae. In angiosperms, they were frequently found in commelinids, Ranunculales, superrosids and Ericales, and only occasionally in the superasterids (Fig. 1 and Supplementary Fig. 6).

Terpenoids occur abundantly in several diverse groups of seed plants. In gymnosperms, they were widely distributed in conifers; in monocots, they were frequent in commelinids; and, in eudicots, they were widely present in fabids and superasterids (Fig. 1 and Supplementary Fig. 7).

Steroids were rare in gymnosperms, but widespread in angiosperms. In the angiosperms, they were frequent in commelinids, Piperales, Ranunculales, Myrtales, Sapindales, Rosales, Gentianales and Ericales (Fig. 1 and Supplementary Fig. 8).

### Phylogenetic signal in PSMs across seed plants

All eight PSM classes tested exhibit a distribution that is significantly different from one expected under a Brownian-motion model (*p__Brownian motion_* < 0.05), while only six of the eight tested PSM classes show a distribution significantly different

from a random distribution (*p__Rand__om_* < 0.05), with steroids and phenylpropanoids being the outliers (*p__Rand__om_* > 0.05). The remaining six PSM classes had a D value of between 0.672 and 0.798 (Table 1), suggesting that these PSMs show a weak clustering when all seed plants are considered, with some approaching, but still significantly different from, a random distribution. Across seed plants as a whole, and the subset of angiosperms only, a significant phylogenetic signal was observed for all eight PSM classes, although some of the smaller subsets tested showed no significant phylogenetic signal for some of the eight PSMs.

**Table 1.** Phylogenetic signals for eight classes of PSMs across seed plants.

		*p-*values	
PSMs	D statistic	Random shuffle	Brownian motion
Alkaloids	0.785424	0.016^*^	0.001
Phenolic acids	0.788535	0.028^*^	<0.001
Flavonoids	0.762775	0.014^*^	<0.001
Phenylpropanoids	0.826109	0.063	<0.001
Quinones	0.694286	0.005^*^	0.006
Tannins	0.798074	0.036^*^	<0.001
Terpenoids	0.671852	0.002^*^	0.001
Steroids	0.975770	0.400	<0.001

^*^ *p__Rand_*_om_ < 0.05 means that the corresponding D value is statistically significant.

Of the 17 sub-clades of seed plants tested, a statistically significant phylogenetic signal was detected in 16 clades for at least one PSM (with the Lamiids being the one exception; for details, see Supplementary Table 3 and Fig. 2a). Across large clades, as the number of families increases, the number of observed cases of significant phylogenetic signal also increases; decreasing the scope from large-scale (e.g. eudicots and superasterids) to ordinal-level (e.g. Malpighiales and Caryophyllales) comparisons showed a reduction in the number of observations with a significant phylogenetic signal (Fig. 2a). A phylogenetic signal was observed in higher taxonomic orders such as the eudicots, core eudicots, superasterids and asterids, with all eight PSMs showing a phylogenetic signal; monocots, superrosids and rosids show a significant phylogenetic signal in seven of the eight PSMs; and, for many groups above the ordinal level, such as malvids, fabids and campanulids, and at the ordinal level (Malpighiales and Caryophyllales), a significant phylogenetic signal was observed in one to four of the eight PSMs (Fig. 2a and Supplementary Table 3).

The scale investigated changes the interpretations of phylogenetic signals, such as, when looking at seed plants, angiosperms, eudicots, core eudicots and superrosids, all PSMs show D values lower than 1, indicating weak phylogenetic clustering (0 < D < 1). Conversely, when looking at groups such as commelinids, fabids and campanulids, only a few PSMs show a significant phylogenetic signal, with most PSMs having an observed phylogenetic signal reflecting a random distribution (D value close to 1), with a few examples of overdispersion with D values >1 (Supplementary Table 3 and Fig. 2a).

In general, quinones and terpenoids tend to show phylogenetic conservation across different taxonomic scales (e.g. malvids and campanulids), while steroids tend to be randomly distributed across most scales. On lower taxonomic scales, tannins appear to be randomly distributed but, at the level of eudicots and above, they appear to show weak phylogenetic clustering. Only quinones, terpenoids and phenolic acids show a strong phylogenetic signal suggesting extreme phylogenetic conservation (Supplementary Table 3 and Fig. 2a).

After recalculating the D statistic with different subsamples to test the robustness of our estimates based on taxonomic sampling, we found that recalculating the D statistic showed little fluctuation in the recovered D values and no differences in the level of significance due to the amount of missing or unobserved data with 50%, 70%, 80% and 95% of observed cases being used and the remaining set as absent or unknown.

## DISCUSSION

### Distribution and diversification of PSM classes in seed plants

**Figure 3.**
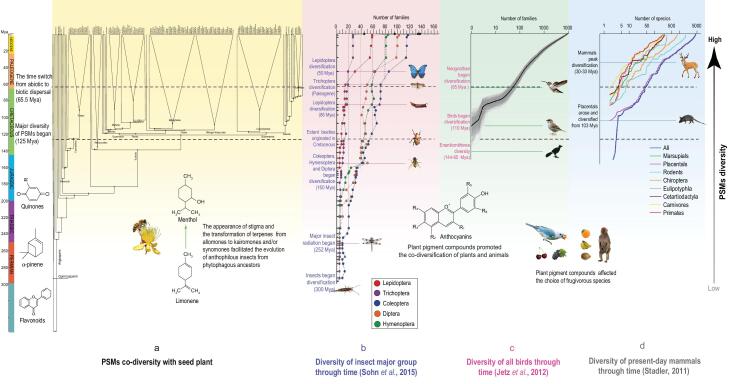
Diversification of seed plants compared to the diversification of herbivorous and pollinating animals. (a) PSM and seed-plant diversification, (b) diversification of five insect groups (according to Sohn *et al.* [40]) important in pollination (Trichoptera, Coleoptera, Diptera, Lepidoptera and Hymenoptera), (c) bird diversification according to Jetz *et al.* [49] and (d) mammal diversification according to Stadler [61].

Based on the phylogenetic placement of PSMs and applicable fossils (see below), seven of the eight classes were likely present in the common ancestor of seed plants and seven PSM classes (quinones, phenylpropanoids, flavonoids, phenolic acids, steroids, terpenoids and alkaloids) experienced rapid expansion during the radiation of the angiosperms in the Late Cretaceous and subsequent diversification (as shown in Fig 3a). Quinones appear to have preceded the origin of chloroplasts, with ubiquinones and plastoquinone, which function respectively in photosynthesis and mitochondrial electron transport [15,16]. The broad sharing of quinones and other PSM classes among various living organisms appears to have involved symbiotic origins of mitochondria and chloroplasts, and thus subsequent differentiation of the eukaryotic groups that acquired them. For example, quinone diversification appears to have been associated with the rapid evolution that occurred relatively early in the history of seed plants (Figs. 3a and Supplementary Fig. 5). Only a few quinones are known in ferns and conifers [17], but perhaps this is due more to insufficient investigation in these groups to date than an actual lack of quinones. Quinones are clearly frequent and widely distributed in angiosperms [17], although with many of them (e.g. naphthoquinones) involved in allelopathy among plants [15].

Phenylpropanoids likely originated in early land plants and are found only in their descendants [18,19]. Flavonoids shield photosynthesis and other metabolic activities from UV radiation, thus enabling the evolution and survival to land

 

[19], and occur in all extant land plants, including bryophytes, pteridophytes and lycophytes [20]. Simple flavonoids, such as biflavonyls, occur in all of the previously mentioned groups as well as in gymnosperms, while complex and diverse flavonoid structures are known only in angiosperms [20]. As with quinones, most flavonoid diversification appears to have accompanied the explosive radiation of angiosperms.

Steroids and phenolic acids are first known from Paleozoic fossils [18] and are ubiquitous in living plants. Understanding the patterns of variation in phenolic acids remains challenging, however, due to their structural complexity and frequent occurrence as members of chemical complexes with proteins, carbohydrates, lipids and other molecules [21]. Although phenolic acids are of special interest with their wide occurrence in foods obtained from plants, including fruits, vegetables, coffee, wine, beer and olive oil, our comparative analysis of their distribution remains incomplete. One hypothesis is that they likely occur in far more than the 167 families in which they have been detected thus far. The limited distribution of phenolic acids in gymnosperms (6 of 12 families) may also be an artifact of insufficient detection analyses (Supplementary Table 2). According to the available data, steroids are distributed sporadically among different groups of seed plants (Fig. 1 and Supplementary Fig. 8) and more diverse in eudicots than in magnoliids.

Terpenoids are found in essentially all living organisms and are ubiquitous in seed plants (Fig. 1 and Supplementary Fig. 7); familiar examples with significant functions include chlorophyll, abscisic acid and gibberellins. These compounds are responsible for most of the fragrances that plants produce and thus are common in everyday products. Terpenoids were likely present in the common ancestor of all land plants, with Otto *et al.* [22] reporting terpenoids from Eocene and Miocene conifer fossils. Terpenoids also occur in *Ginkgo biloba*, whose origins can be traced back to the Jurassic period [23].

Alkaloids represent yet another class of PSMs that occur in all major groups of organisms [24] but became extraordinarily diverse as the angiosperms radiated (Fig. 1 and Supplementary Fig. 1). The recorded occurrence of alkaloids in vascular plants other than angiosperms is infrequent [25] but likely insufficient sampling has been conducted to date to draw significant conclusions. Judging from the diversity of alkaloids in both early angiosperms such as magnoliids and in more modern groups such as Ranunculales and superrosids (Fig. 1 and Supplementary Fig. 1), this group of chemicals has been diversifying with angiosperms from the beginning.

### Phylogenetic signals across the seed-plant tree of life

The observed significant phylogenetic signal for PSMs can be explained by phylogenetic conservatism or evolutionary constraints on the development of the compounds' biosynthetic pathways [11], implying that heredity and natural selection could bring about higher levels of phylogenetic signal. Conversely, finding a weaker phylogenetic signal than expected under Brownian motion suggests that diversifying selection may be occurring.

Overall, the phylogenetic signal of PSMs in seed plants and smaller clades show a D statistic of between 0 and 1 (Supplementary Table 3 and Fig. 2a), indicating that PSMs are weakly clustering (D value approaching 0) or randomly distributed (D value close to 1). The current heterogeneous distribution of PSMs likely was governed by historical conditions during the evolution of the plants but the necessity for the genes cannot be ignored. Tohge *et al.* [26] suggested that the evolution of phenolics has been shaped by prevailing environmental conditions and ecological niches, as well as other key factors including gene duplication and *cis*-regulatory evolution. There are several hypotheses that help to explain the observations that PSMs have a random distribution: (i) multiple origins and the probability of horizontal gene transfer [27]; for example, diterpenoid alkaloids are currently only found in distantly related clades: Ranunculaceae (*Aconitum*, *Delphinium*, *Thalictrum*), Rosaceae (*Spiraea japonica* complex), Garryaceae (*Garrya*), Escalloniaceae (*Anopterus*) and Polygonaceae (*Rumex pictus*) [28]; additionally, Pichersky and Lewinsohn [29] insist that members of Rubiaceae (*Coffea arabica*), Theaceae (*Camellia sinensis*), Aquifoliaceae (*Ilex paraguariensis*), Sapindaceae (*Paullinia cupana*), Malvaceae (*Cola acuminata*) and Rutaceae (*Citrus* spp.) evolved caffeine for pollination and seed-dispersal purposes, and many plant families contain cyanogenic glycosides for defense against herbivores due to convergent evolution; (ii) the co-evolution between plant species, plants and their herbivores, and plants and microbes (e.g. bacteria, fungus and virus); for example, Salazar *et al.* [30] found that generalist herbivores played an important role in shaping chemical diversity in the Burseraceae; and (iii) abiotic environmental conditions (e.g. nutrients, light, water) bring about plasticity in PSMs expression; for example, congeneric species in resource-limiting environments evolved low maximal growth rates and invest more in defense traits, while, in high-resource environments, species evolved high maximal growth rates, invest less in defense and are more tolerant to damage [31]. All the factors mentioned above have played important roles alongside heredity in the formation of observed high PSM diversity.

Across seed plants as a whole, closely related species tend to have similar PSMs in the following categories: alkaloids, phenolic acids, flavonoids, quinones, tannins and terpenoids (Table 1). Most cases in which a significant phylogenetic signal was observed occur at higher taxonomic levels and exhibit a signal of weak clustering (i.e. 0 < D < 1), likely reflecting the high diversity of most PSMs. The high diversity and low signal across most PSMs are unsurprising given that seed plants are a large, globally distributed and diverse clade inhabiting every terrestrial environment available and many aquatic environments. These results indicate that these complex and diverse environments are important external factors for the formation of observed high PSM diversity. Their exceptional biochemical diversity may result from strong diversifying selection during seed-plant diversification and/or relatively weak evolutionary constraints on PSMs [32].

The finding of a significant phylogenetic signal for certain PSM classes in specific clades likely reflects either strong conservation of PSM compounds and/or bursts of diversification. The ability to distinguish between these two possibilities is difficult given the undertaken approach plus different processes and rates can produce similar results in terms of observed phylogenetic signals [33]. For example, terpenoids exhibit weak phylogenetic clustering in rosids (D = 0.292) and asterids (D = 0.641; Fig. 2a and Supplementary Table 3), which can be partially explained by the abundance of diterpenes in rosids [34] and the abundance of iridoids in asterids [35]. Other PSM classes show similar significant patterns, such as quinones in malvids (D = –0.775), Caryophyllales (D = –0.389) and superasterids (D = 0.582; Fig. 2a and Supplementary Table 3), which is driven by the presence of polyketide-derived anthraquinones in malvids and anthraquinones derived from shikimic and isochorismic acids in certain families of superasterids (including Caryophyllales) [15].

Wink [36] indicated that 20%–30% of higher plants have been investigated for phytochemistry, while our results show that ∼70% of families of seed plants have been investigated. The broad-scale nature of our study, and the many gaps in our knowledge of the presence or absence of certain groups of chemicals in plant groups, inhibits our ability to detect significant phylogenetic signals across all scales of inquiry. A very clear and well-known example involves the restriction of betalains to most families of Caryophyllales (i.e. the earlier Centrospermae) [37]. In most species, the anthocyanin pigments that are widespread in all other angiosperms have been replaced by betalains. As analyses of PSM distributions increase, along with structural studies of the molecules, additional robust examples similar to betalains will doubtless be encountered.

### PSMs and animal co-diversification

For over 50 years, it has been well understood that PSMs acting as defensive compounds in land plants have played a major role in the diversification of both the plants and animal groups that rely on them [1,3]. Co-evolution or the process of stepwise evolution that is set in motion when animal herbivores gain the ability to feed on plants that are largely protected from most other groups of herbivores by the PSMs they produce and thus gain the ability to radiate and diversify on the plant group [38] has a rich history in the literature, with literally thousands of papers having been written on the subject over the past few decades. With the recently gained ability to analyse phylogenies on both sides of the co-evolutionary race and accurate analysis of the protective compounds, studies demonstrating co-evolution have become increasingly precise and the ubiquity of the process has become evident. What remains is further consideration of the patterns and their history at deep taxonomic levels in both plants and their herbivores, and a further investigation into the evolutionary processes that shaped the pathways of secondary metabolites [39].

The weak phylogenetic signal suggests that the interaction between plants and animals during evolution may also be an important impetus to form the current high PSM diversity; our analyses are consistent with the hypothesis of herbivore/plant co-diversification. For example, although the major insect radiation began about 250 Mya [40,41], two shifts in the diversity of Lepidoptera occurred ∼85 and ∼52 Mya, the major diversification of Coleoptera, Diptera and Hymenoptera began ∼150 Mya [40] (Fig. 3b). The major diversification of alkaloids, terpenoids, tannins and flavonoids occurred concomitantly with these events throughout the Late Cretaceous and Tertiary (Fig. 3a). Condamine *et al.* [41] postulated that the diversification of angiosperms did not lead to an immediate increase in insect diversification within major groups. In contrast, Zhang *et al.* [42] suggested that angiosperm diversification in the Cretaceous helped drive the hyperdiversity of herbivorous beetles, with ∼64% of the extant families of beetles originating during this period.

Pollination systems, which are often characteristic of species or clades, can change in a short span of evolutionary time. Volatile terpenes often are important in attracting insects and variable among species within many groups of plants (Fig. 3). We hypothesize that the development of volatile terpenes from allomones to kairomones and synomones has accompanied the evolution of anthophilous insects from their phytophagous ancestors (Fig. 3); extant pollinator groups developed phytophagy before becoming flower visitors. Likewise, pollen carrying appeared subsequent to the development of stigmas and the capacity of flowers to emit volatile terpenoids [43]. Courtois [44] found that at least two major episodes of diversification of volatile terpenes may have occurred in tropical angiosperm trees: one in magnoliids ∼122–125 Ma and the other in Sapindales ∼70 Ma. Coincidentally, the major co-evolution episode involving pollinating insects and angiosperms happened during the same periods of the Cretaceous [45]. To sum up, our analyses suggest that the evolution of the PSM toolkit in angiosperms helped to promote their explosive diversification and that of their pollinating insects (Fig. 3a and b).

Most flower colors, with a few exceptions (e.g. yellow flowers with carotenoids or reddish/purplish flowers with betalains), result from anthocyanins—a class of flavonoids that form the largest group of water-soluble natural pigments [46]. Anthocyanins play an important role in attracting insects to flowers, including the frequent ultraviolet floral markings that are not visible to human eyes but clearly visible to most insects [46]. Flowers awaiting pollination are often brightly colored, while becoming dark and much less conspicuous once they have been fertilized. The co-evolution of floral anthocyanins and pollinators has been important and ongoing for both groups since the Late Cretaceous [47]. Flowers regularly pollinated by birds are often red, signaling large energetic reserves, while being invisible to most insects [48]. Red, ripe fruits stand out vividly for birds (and for humans) but fade into the background of green leaves for insects. The colors of fruits and flowers became more diverse and began to include more red as the diversification of modern birds took place from the Upper Cretaceous Period onward with a strong increase in diversification rates from ∼50 Mya to the near present [49]. The increased rates of diversification in modern birds coincide with angiosperm diversification, with a great deal of co-evolution taking place between both groups (Fig. 3a–c).

A number of studies have noted that the evolution of seed dispersal by birds and mammals has likely contributed significantly to angiosperm diversification and to co-evolution between plants and animals [50]. Similarly, the diversification of modern mammals—a mainly Tertiary phenomenon—has been accompanied by a great deal of co-evolutionary diversification in plants. In particular for primates, frugivory seems to have predominated in some of the early-diverging primate lineages and affected their characteristics [51] (Fig. 3a and d).

### Phylogenetic signals in PSMs for bioprospecting

One traditional method of bioprospecting consists of random taxon selection followed by phytochemical screening or biological assays and/or following up reports of biological activity or ethnomedical (traditional medicine) uses of plants [52]. Although ethnomedicine-based screens are expected to lead to high success rates [52,53], the plants tested are often found not to be pharmaceutically effective [53]. Since related species often share similar biochemical profiles [5,8,14], employing a phylogenetic relationship as a bioprospecting criterion should provide a solid guide to identifying species producing similar chemical compounds.

Based on large-scale patterns, our results indicate that a phylogenetic signal is weak, but there are some branches or clades (e.g. malvids) and certain PSMs showing strong phylogenetic signals (e.g. phenolic acids in fabids). The existence of a strong phylogenetic signal in PSMs may provide indirect evidence of underlying bioactivity and biochemical properties [7]. Such signals can therefore be a useful guide in studying novel natural products [5,7], finding new potentially drug-producing groups of plants [8], and certainly in searching for specific PSMs in related plants [7].

### Caveats

We have collected and analysed a comprehensive PSM data set but, as mentioned above, many gaps remain in our knowledge of PSM diversity in seed plants. In addition, the D statistic is sensitive to errors in the phylogeny utilized [54]. Today, however, most relationships among seed-plant families have become clear and are highly supported by increasing amounts and multiple kinds of molecular data. As additional PSM data are reported, it will be valuable to re-evaluate phylogenetic signals among all eight biochemical groups, and especially to explore signals at lower taxonomic levels (e.g. within subfamilies or genera) and within more specific groups of chemicals.

## MATERIALS AND METHODS

### PSM data collection

We aggregated data for four major chemical groups (which were further divided into eight classes) of PSMs that differ in their biosynthetic pathways: alkaloids, phenolics (which were subdivided into phenolic acids, flavonoids and tannins), isoprenoids (which were subdivided into terpenoids and steroids) and quinones. The eighth class, phenylpropanoids, were grouped as lignans and coumarins together, but their phenylpropanoid counterparts, the flavonoids, which are widespread and extraordinarily important in seed plants, were treated separately. For details of PSM classification, see Supplementary Table 1. Some PSMs such as glucosinolates, cyanogenic glycosides, lignin, gibberellins, abscisic acid and sterols were not considered in our study because either they are ubiquitous or because too little is known about their distribution in seed plants to be informative. We aggregated data on PSM presence from journals, books (Supplemental Table 4) and a chemistry database (SciFinder: https://scifinder.cas.org/). The PSM data matrix used in the current analyses is provided in Supplementary Table 2.

### PSM mapping

A time-calibrated family-level phylogeny was used for tracking the evolutionary patterns of PSMs across seed plants and calculating the phylogenetic signal of each PSM. We explored phylogenetic patterns of PSMs in seed plants at the family level because (i) seed-plant-family relationships are largely well resolved (e.g. APG IV [55]) and (ii) PSM distribution has not yet been worked out in detail for many of these clades. Qian and Zhang [56] suggested that using a family-level phylogeny might be informative for the phylogenetic analyses of biological and functional traits when a species-level phylogeny is unattainable. The family-level phylogenies of Qian and Zhang [56] (see Supplementary Appendix 3 of their paper) and Zanne *et al.* [57] were selected because they include broad sampling of all families of extant seed plants, representing the most comprehensive phylogenies available at the onset of this study. A recent larger seed-plant phylogeny was published by Smith and Brown [9] during the course of this project, but the higher taxonomic relationships and divergence time estimates are congruent with the phylogenies used during our analyses. The temporal diversification of PSMs was estimated by mapping PSMs on the time-calibrated phylogeny of Zanne *et al.* [57], in which the divergence times were estimated for 32 223 species of land plants. We collapsed the Zanne *et al.* [57] tree to the family level for our analyses. The standard of phylogenetic nomenclature of the angiosperm was according to APG IV [55].

We collected data on PSMs at the species or generic level for all plants but the familial level was the unit of analysis for all reconstructions. PSM characters were coded as binary traits: 1 if the trait was present in at least one taxon within the family and 0 if the trait was absent from, or unclear in, all known taxa within the family. Character-state distributions along the phylogeny were performed using the ‘trait.plot’ function as implemented in the R package diversitree version 0.9–9 [58].

### Testing for phylogenetic signals

There are multiple methods to calculate phylogenetic signals (i.e. Moran's I, Blomberg's K and Pagel's λ) [59] but only the D statistic is designed for discrete binary data and is therefore suitable for our work data [54]. We used the D statistic [54] to test for phylogenetic signals for each of the eight classes of PSMs using the package Caper version 1.0.1 [60] in R. The significance of the difference (*p*-value) between observed and expected values was investigated by using the variance of phylogenetically independent contrasts relative to 1000 tip-shuffling randomizations. Only when *p* < 0.05 is the D value statistically significant. A value of D < 0 indicates that a PSM class is phylogenetically conserved (i.e. there is a strong phylogenetic signal); a value of D = 0 indicates that a PSM class is clustered as one would expect if the continuous trait had evolved under a Brownian-motion model and then was converted into a binary trait using a threshold that reproduces the prevalence of the observed trait; a D value of 1 indicates a random distribution of a PSM class across the tips; and a value of D > 1 indicates phylogenetic overdispersion of a PSM class [54]. Because the D statistic is not powerful for trees with <25 tips [54], we calculated D statistics for 18 major clades of seed plants (Supplementary Table 3). To explore the robustness of phylogenetic signals with different levels of taxonomic sampling, we subsampled the observed presence data for PSMs to include 50%, 70%, 80% and 95% of observed cases, setting the rest to missing or unknown.

## Supplementary Material

nwaa105_Supplemental_File

## References

[1] Hartmann T . From waste products to ecochemicals: fifty years research of plant secondary metabolism. Phytochemistry 2007; 68: 2831–46. 17980895 10.1016/j.phytochem.2007.09.017

[2] Smilanich AM , Fincher RM, Dyer LA. Does plant apparency matter? Thirty years of data provide limited support but reveal clear patterns of the effects of plant chemistry on herbivores. New Phytol 2016; 210: 1044–57. 26889654 10.1111/nph.13875

[3] Grubb PJ . A positive distrust in simplicity—Lessons from plant defences and from competition among plants and among animals. J Ecol 1992; 80: 585–610.

[4] Moore BD , Andrew RL, Kulheim C et al. Explaining intraspecific diversity in plant secondary metabolites in an ecological context. New Phytol 2014; 201: 733–50. 24117919 10.1111/nph.12526

[5] Saslis-Lagoudakis CH , Savolainen V, Williamson EM et al. Phylogenies reveal predictive power of traditional medicine in bioprospecting. Proc Natl Acad Sci USA 2012; 109: 15835–40. 22984175 10.1073/pnas.1202242109 PMC3465383

[6] Agrawal AA . Macroevolution of plant defense strategies. Trends Ecol Evol 2007; 22: 103–9. 17097760 10.1016/j.tree.2006.10.012

[7] Saslis-Lagoudakis CH , Klitgaard BB, Forest F et al. The use of phylogeny to interpret cross-cultural patterns in plant use and guide medicinal plant discovery: an example from *Pterocarpus* (Leguminosae). PLoS One 2011; 6: e22275. 21789247 10.1371/journal.pone.0022275 PMC3138776

[8] Zhu F , Qin C, Tao L et al. Clustered patterns of species origins of nature-derived drugs and clues for future bioprospecting. Proc Natl Acad Sci USA 2011; 108: 12943–8. 21768386 10.1073/pnas.1107336108 PMC3150889

[9] Smith SA , Brown JW. Constructing a broadly inclusive seed plant phylogeny. Am J Bot 2018; 105: 302–14. 29746720 10.1002/ajb2.1019

[10] José Alexandre F. Diniz-Filho Thiago Santos, Thiago Fernando Rangel et al. A comparison of metrics for estimating phylogenetic signal under alternative evolutionary models. Genet Mol Biol 2012; 35: 673–9. 23055808 10.1590/S1415-47572012005000053 PMC3459419

[11] Losos JB . Phylogenetic niche conservatism, phylogenetic signal and the relationship between phylogenetic relatedness and ecological similarity among species. Ecol Lett 2008; 11: 995–1003. 18673385 10.1111/j.1461-0248.2008.01229.x

[12] Kamilar JM , Cooper N. Phylogenetic signal in primate behaviour, ecology and life history. Philos Trans R Soc Lond B Biol Sci 2013; 368: 20120341. 23569289 10.1098/rstb.2012.0341 PMC3638444

[13] Senior JK , Potts BM, Davies NW et al. Phylogeny explains variation in the root chemistry of *Eucalyptus* species. J Chem Ecol 2016; 42: 1086–97. 27577951 10.1007/s10886-016-0750-7

[14] Rønsted N , Savolainen V, Mølgaard P et al. Phylogenetic selection of *Narcissus* species for drug discovery. Biochem Syst Ecol 2008; 36: 417–22.

[15] Seigler DS. Plant Secondary Metabolism. New York: Springer Science & Business Media, 1998.

[16] Kruk J , Strzałka K. Occurrence and function of α-tocopherol quinone in plants. J Plant Physiol 1995; 145: 405–9.

[17] Thomson RH. Distribution of naturally occurring quinones. Int J Clin Pharm 1991; 13: 70–3. 10.1007/BF01974983 1870945

[18] Niklas KJ , Gensel PG. Chemotaxonomy of some Paleozoic vascular plants. Part I: Chemical compositions and preliminary cluster analyses. Brittonia 1976; 28: 353–78.

[19] Weng JK. The evolutionary paths towards complexity: a metabolic perspective. New Phytol 2014; 201: 1141–9. 23889087 10.1111/nph.12416

[20] Marin P. Flavonoids as taxonomic markers in flowering plants. Glas Inst Bot I Baste Univ U Beoggradu 1996; 30: 19–37.

[21] Luthria DL , Pastor-Corrales MA. Phenolic acids content of fifteen dry edible bean (*Phaseolus vulgaris* L.) varieties. J Food Compos Anal 2006; 19: 205–11.

[22] Otto A , D.White J, R.T.Simoneit B. Natural product terpenoids in Eocene and Miocene conifer fossils. Science 2002; 297: 1543–5. 12202827 10.1126/science.1074225

[23] Carrier DJ , Beek TAv, Heijden Rvd et al. Distribution of ginkgolides and terpenoid biosynthetic activity in *Ginkgo biloba*. Phytochemistry 1998; 48: 89–92.

[24] Vickery ML , Vickery B. Secondary Plant Metabolism. London and Basingstoke: The Macmillan Press Ltd, 1981.

[25] Lazur’Evskii G , Terent’Eva I. Distribution of alkaloids in the higher plants. Chem Nat Comp 1974; 10: 342–9.

[26] Tohge T , Watanabe M, Hoefgen R et al. The evolution of phenylpropanoid metabolism in the green lineage. Crit Rev Biochem Mol Biol 2013; 48: 123–52. 23350798 10.3109/10409238.2012.758083

[27] Yue J , Hu X, Sun H et al. Widespread impact of horizontal gene transfer on plant colonization of land. Nat Commun 2012; 3: 1152. 23093189 10.1038/ncomms2148 PMC3493653

[28] Wang FP , Liang XT. C20-diterpenoid alkaloids. In: Cordell GA (ed). The Alkaloids: Chemistry and Biology. New York: Academic Press, 2002, 1–280. 10.1016/s0099-9598(02)59008-8 12561418

[29] Pichersky E , Lewinsohn E. Convergent evolution in plant specialized metabolism. Annu Rev Plant Biol 2011; 62: 549–66. 21275647 10.1146/annurev-arplant-042110-103814

[30] Salazar D , Lokvam J, Mesones I et al. Origin and maintenance of chemical diversity in a species-rich tropical tree lineage. Nat Ecol Evol 2018; 2: 983–90. 29760441 10.1038/s41559-018-0552-0

[31] Orians CM , Ward D. Evolution of plant defenses in nonindigenous environments. Annu Rev Entomol 2010; 55: 439–59. 19737084 10.1146/annurev-ento-112408-085333

[32] Carmona D , Lajeunesse MJ, Johnson MTJ. Plant traits that predict resistance to herbivores. Funct Ecol 2011; 25: 358–67.

[33] Revell LJ , Harmon LJ, Collar DC. Phylogenetic signal, evolutionary process, and rate. Syst Biol 2008; 57: 591–601. 18709597 10.1080/10635150802302427

[34] Jonathan G , Tom MJ. Secondary metabolites and the higher classification of angiosperms. Nord J Bot 1983; 3: 5–34.

[35] Stull GW , Schori M, Soltis DE et al. Character evolution and missing (morphological) data across Asteridae. Am J Bot 2018; 105: 470–9. 29656519 10.1002/ajb2.1050

[36] Wink M . Introduction: biochemistry, physiology and ecological functions of secondary metabolites. In: Wink M (ed). Biochemistry of Plant Secondary Metabolism (Second Edition). Chichester: Blackwell Publishing Ltd. 2010, 1–19.

[37] Mabry T . The Betacyanins, a New Class of Red Violet Pigments, and Their Phylogenetic Significance. New York: Roland Press, 1964.

[38] Sussmann RW , Raven PH. Pollination by lemurs and marsupials: an archaic coevolutionary system. Science 1978; 200: 731–6. 17743224 10.1126/science.200.4343.731

[39] Ober D . Seeing double: gene duplication and diversification in plant secondary metabolism. Trends Plant Sci 2005; 10: 444–9. 16054418 10.1016/j.tplants.2005.07.007

[40] Sohn JC , Labandeira CC, Davis DR. The fossil record and taphonomy of butterflies and moths (Insecta, Lepidoptera): implications for evolutionary diversity and divergence-time estimates. BMC Evol Biol 2015; 15: 12. 25649001 10.1186/s12862-015-0290-8 PMC4326409

[41] Condamine FL , Clapham ME, Kergoat GJ. Global patterns of insect diversification: towards a reconciliation of fossil and molecular evidence? Sci Rep 2016; 6: 19208. 26778170 10.1038/srep19208 PMC4725974

[42] Zhang S-Q , Che L-H, Li Y et al. Evolutionary history of Coleoptera revealed by extensive sampling of genes and species. Nat Commun 2018; 9: 1–11. 29335414 10.1038/s41467-017-02644-4 PMC5768713

[43] Pellmyr O , Thien LB. Insect reproduction and floral fragrances: keys to the evolution of the angiosperms? Taxon 1986; 35: 76–85.

[44] Courtois EA , Dexter KG, Paine CET et al. Evolutionary patterns of volatile terpene emissions across 202 tropical tree species. Ecol Evol 2015; 6: 2854–64. 10.1002/ece3.1810 PMC4803801 27069586

[45] Grimaldi D. The co-radiations of pollinating insects and angiosperms in the Cretaceous. Ann Missouri Bot Gard 1999; 86: 373–406.

[46] Winefield C. Anthocyanins: Biosynthesis, Functions, and Applications. New York: Springer-Verlag, 2009.

[47] Ehrlich PR , Raven PH. Butterflies and plants: a study on coevolution. Evolution 1965; 18: 586–608.

[48] Raven PH. Why are bird-visited flowers predominantly red? Evolution 1973; 26: 674. 10.1111/j.1558-5646.1972.tb01975.x 28563347

[49] Jetz W , Thomas GH, Joy JB et al. The global diversity of birds in space and time. Nature 2012; 491: 444–8. 23123857 10.1038/nature11631

[50] Herrery CM. Seed dispersal by animals: a role in angiosperm diversification. Am Nat 1989; 133: 309–22.

[51] Sussman RW , Rasmussen DT, Raven PH. Rethinking primate origins again. Am J Primatol 2013; 75: 95–106. 23184701 10.1002/ajp.22096

[52] Cordell GA , Quinn-Beattie ML, Farnsworth NR. The potential of alkaloids in drug discovery. Phytother Res 2001; 15: 183–205. 11351353 10.1002/ptr.890

[53] Saslis-Lagoudakis CH , Williamson EM, Savolainen V et al. Cross-cultural comparison of three medicinal floras and implications for bioprospecting strategies. J Ethnopharmacol 2011; 135: 476–87. 21457769 10.1016/j.jep.2011.03.044

[54] Fritz SA , Purvis A. Selectivity in mammalian extinction risk and threat types: a new measure of phylogenetic signal strength in binary traits. Conserv Biol 2010; 24: 1042–51. 20184650 10.1111/j.1523-1739.2010.01455.x

[55] APG Group . An update of the Angiosperm Phylogeny Group classification for the orders and families of flowering plants: APG IV. Bot J Linn Soc 2016; 181: 1–20.

[56] Qian H , Zhang J. Using an updated time-calibrated family-level phylogeny of seed plants to test for non-random patterns of life forms across the phylogeny. J Syst Evol 2014; 52: 423–30.

[57] Zanne AE , Tank DC, Cornwell WK et al. Three keys to the radiation of angiosperms into freezing environments. Nature 2014; 506: 89–92. 24362564 10.1038/nature12872

[58] FitzJohn RG . Diversitree: comparative phylogenetic analyses of diversification in R. Methods Ecol Evol 2012; 3: 1084–92.

[59] Tamara M , Sébastien L, Bruno B et al. How to measure and test phylogenetic signal. Methods Ecol Evol 2012; 3: 743–56.

[60] Orme D , Freckleton R, Thomas G et al. Caper: comparative analyses of phylogenetics and evolution in R. R package version 1.0.1. https://CRAN.R-project.org/package=caper(12 March 2018, date last accessed).

[61] Stadler T . Mammalian phylogeny reveals recent diversification rate shifts. Proc Natl Acad Sci USA 2011; 108: 6187–92. 21444816 10.1073/pnas.1016876108 PMC3076834

