# Supplementary material for: Phylogenetic patterns suggest frequent multiple origins of secondary metabolites across the seed-plant ‘tree of life’
Source: Natl Sci Rev. 2020 May 21;8(4):nwaa105. doi: 10.1093/nsr/nwaa105 (PMC8288438; doi:10.1093/nsr/nwaa105)
Supplement: nwaa105_Supplemental_File [file nwaa105_supplemental_file.docx]

Supplementary Information for

**Phylogenetic patterns suggest frequent multiple origins of secondary metabolites across the seed plant “tree of life”**

Yongzeng Zhang^1,2,^†, Tao Deng^1,^†, Lu Sun^1,2,^†, Jacob B Landis^3,4,^†, Michael J Moore^5^, Hengchang Wang^6^, Yuehua Wang^7^, Xiaojiang Hao^8^, Jijun Chen^8^, Shenghong Li^8^, Maonian Xu^9^, Pema-Tenzin Puno^8^, Peter H Raven^10,^* and Hang Sun^1,^*

Correspondence to: sunhang@mail.kib.ac.cn (H. Sun), peter.raven@mobot.org (P. Raven)

**This file** **includes:**

**Fig. S1.** **Distribution of alkaloids across seed plant phylogeny**

**Fig. S2. Distribution of flavonoids across seed plant phylogeny**

**Fig. S3. Distribution of phenolic acids across seed plant phylogeny**

**Fig. S4. Distribution of phenylpropanoids across seed plant phylogeny**

**Fig. S5. Distribution of quinones across seed plant phylogeny**

**Fig. S6. Distribution of tannins across seed plant phylogeny**

**Fig. S7. Distribution of terpenoids across seed plant phylogeny**

**Fig. S8. Distribution of steroids across seed plant phylogeny**

Table S1. Classification of eight class of PSM

**Table S2. Original data for PSMs in seed plant.**

Table S3. Original results for D values in each major seed plant clade tested.

Table S4. Original sources for PSM data


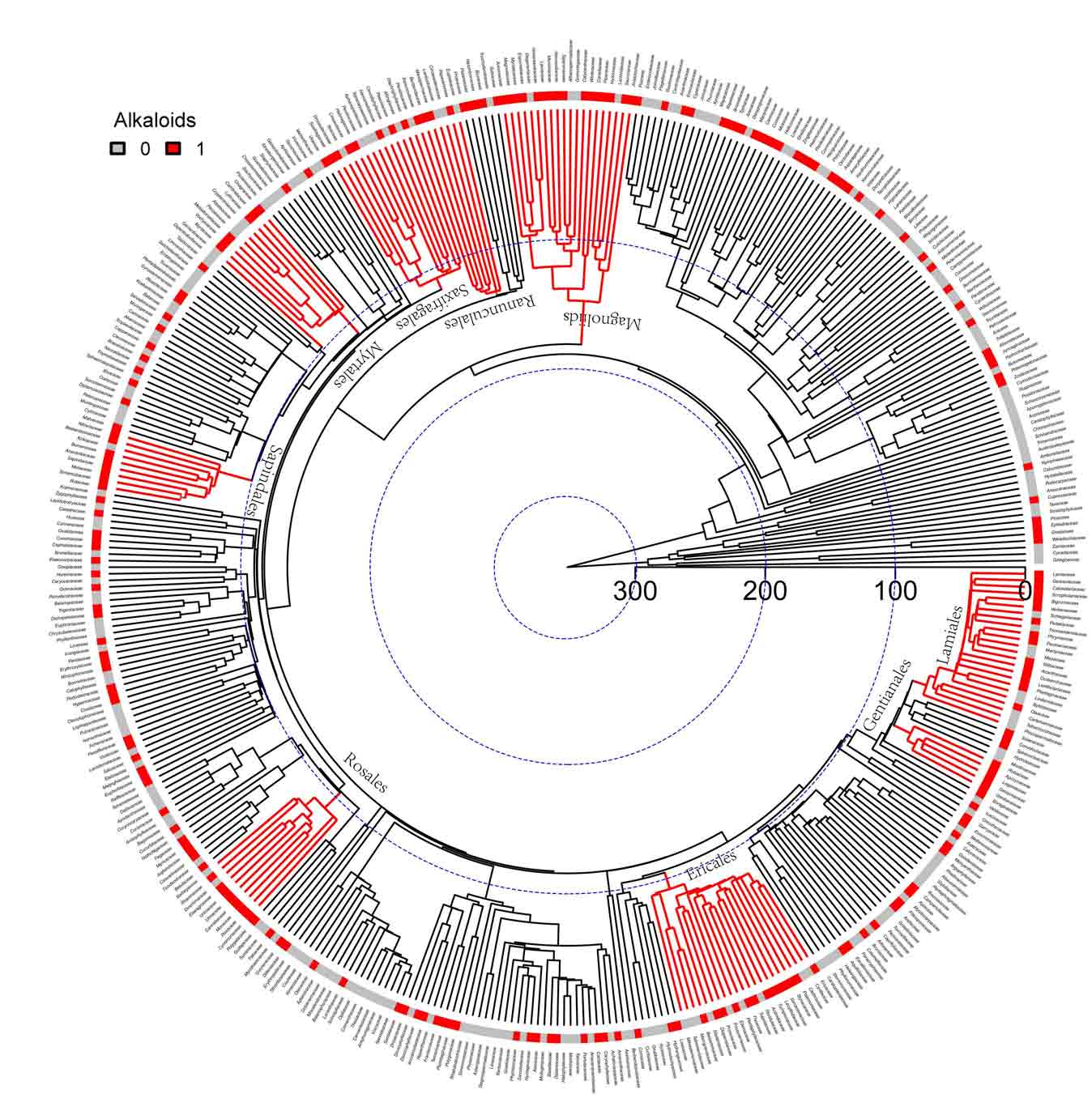


Fig. S1. Distribution of alkaloids across seed plant phylogeny. Clades in which alkaloids are determined to be present are colored and labeled.


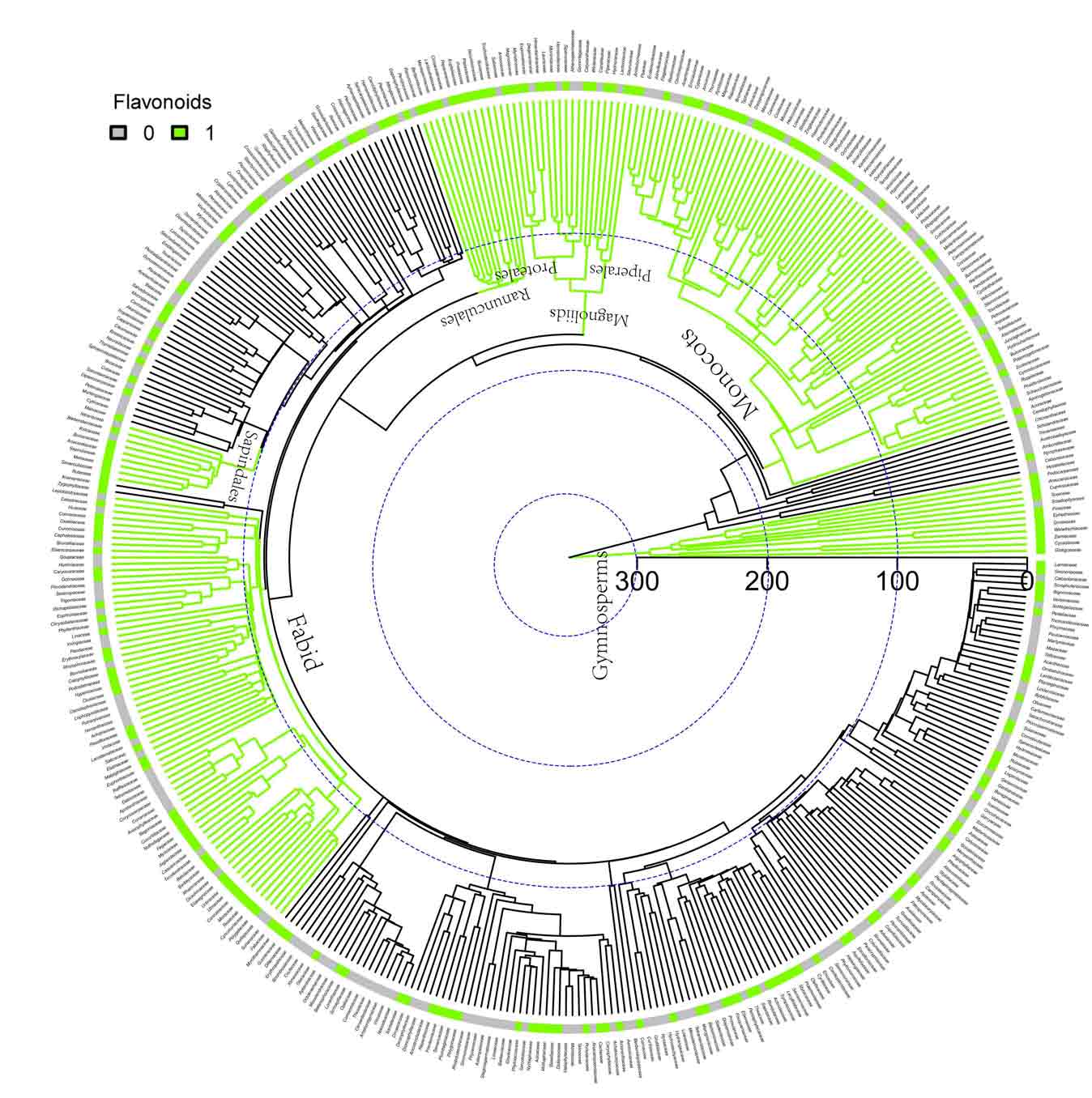


**Fig. S2. Distribution of flavonoids across seed plant phylogeny.** Clade in which flavonoids are determined to be present colored and labeled.


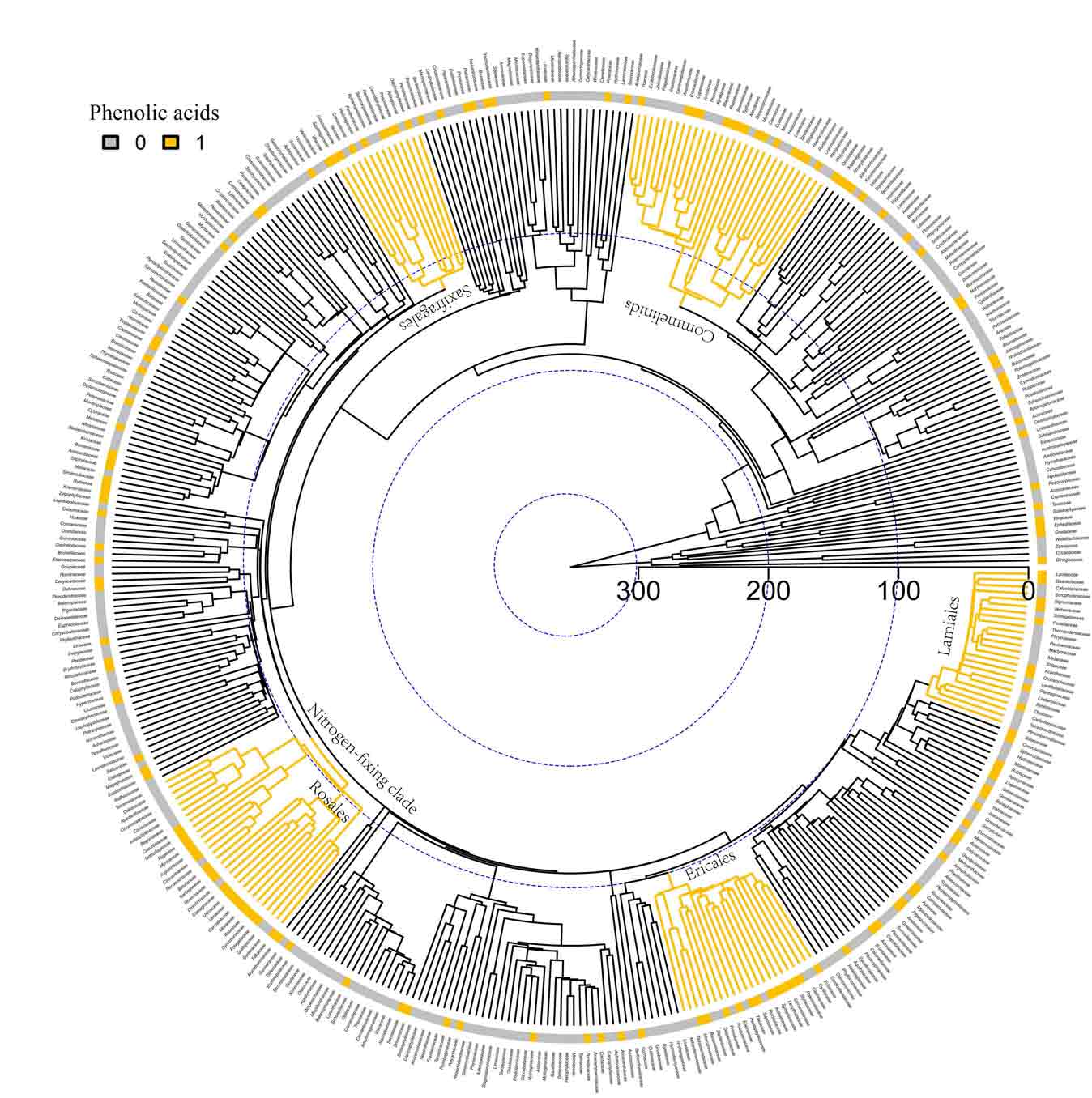


**Fig. S3. Distribution of phenolic acids across seed plant phylogeny.** Clades in which phenolic acids are determined to be present are colored and labeled.


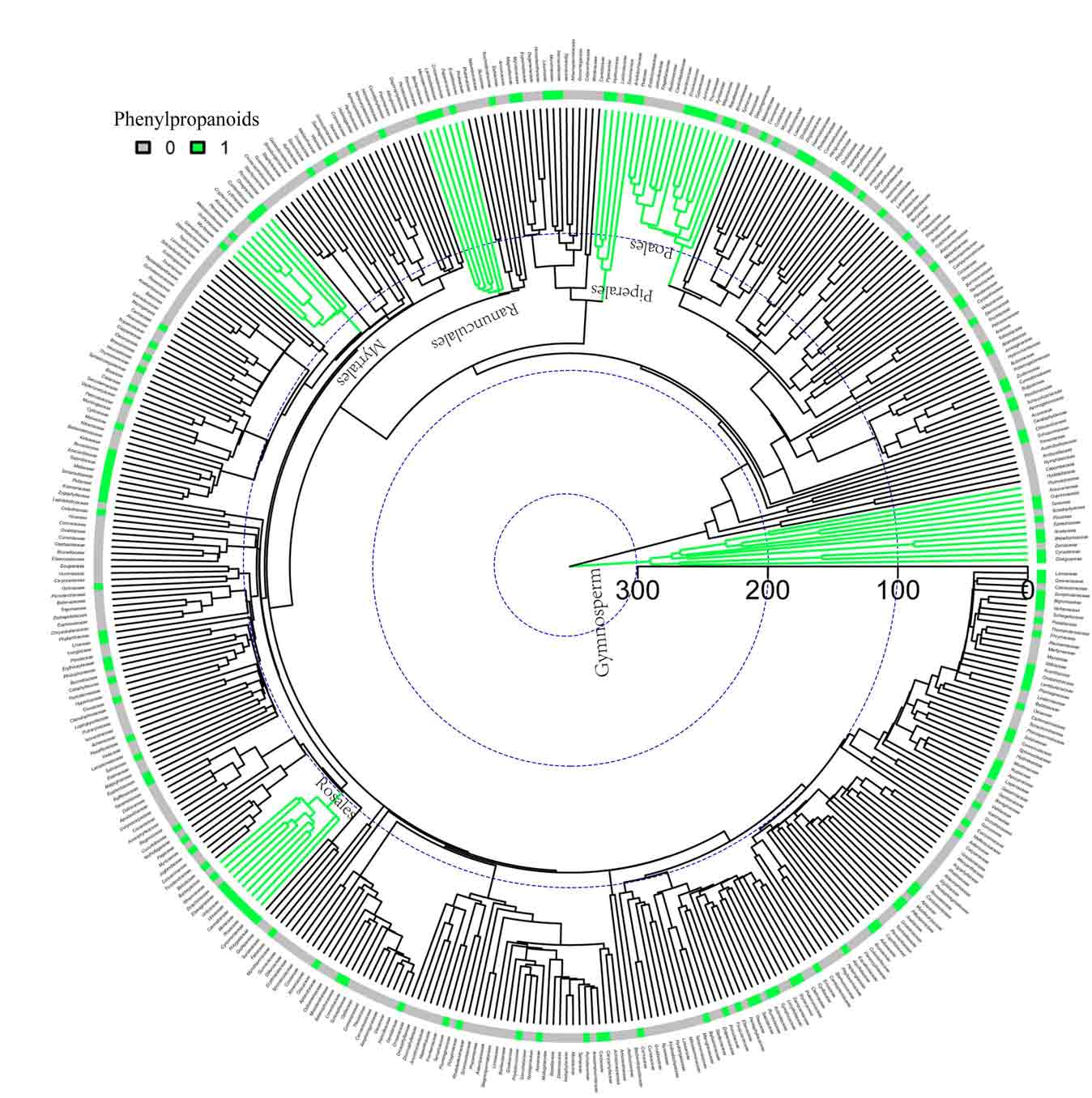


**Fig. S4. Distribution of phenylpropanoids** **across seed plant phylogeny.** Clades in which phenylpropanoids are determined to be present are colored and labeled.


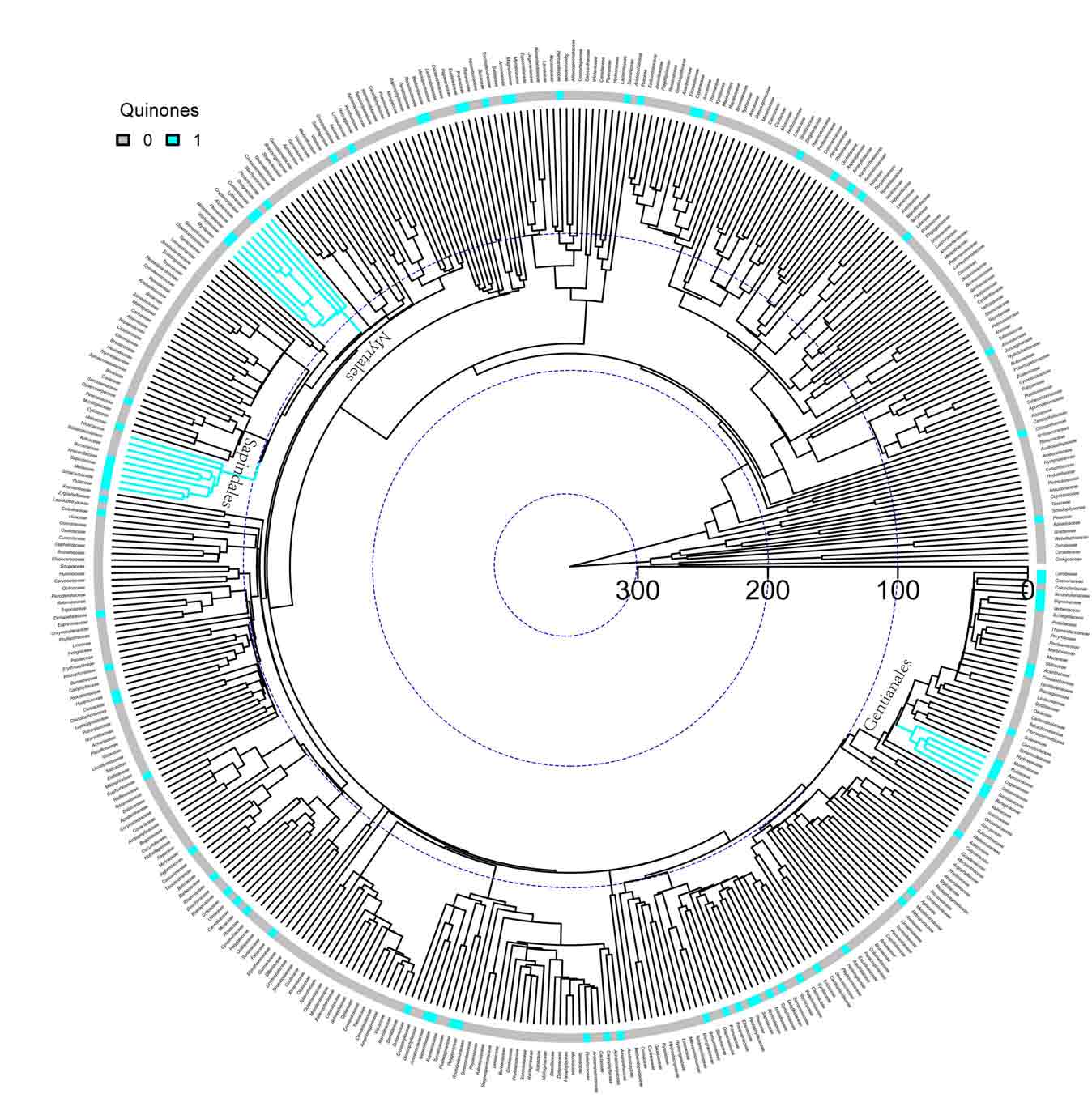


**Fig. S5. Distribution of quinones across seed plant phylogeny.** Clades in which quinones are determined to be present are colored and labeled.


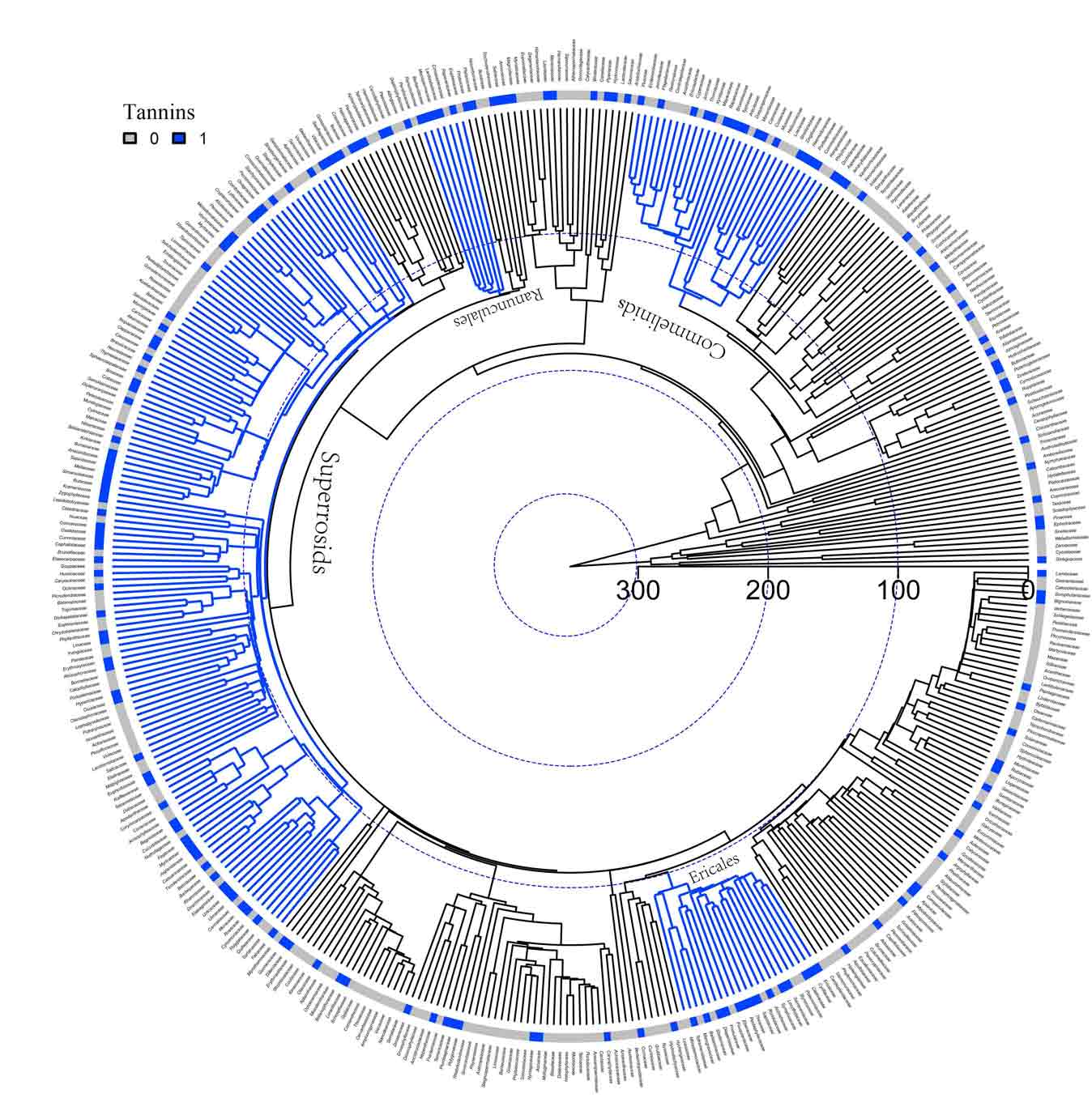


**Fig. S6. Distribution of tannins across seed plant phylogeny.** Clades in which tannins are determined to be present are colored and labeled.


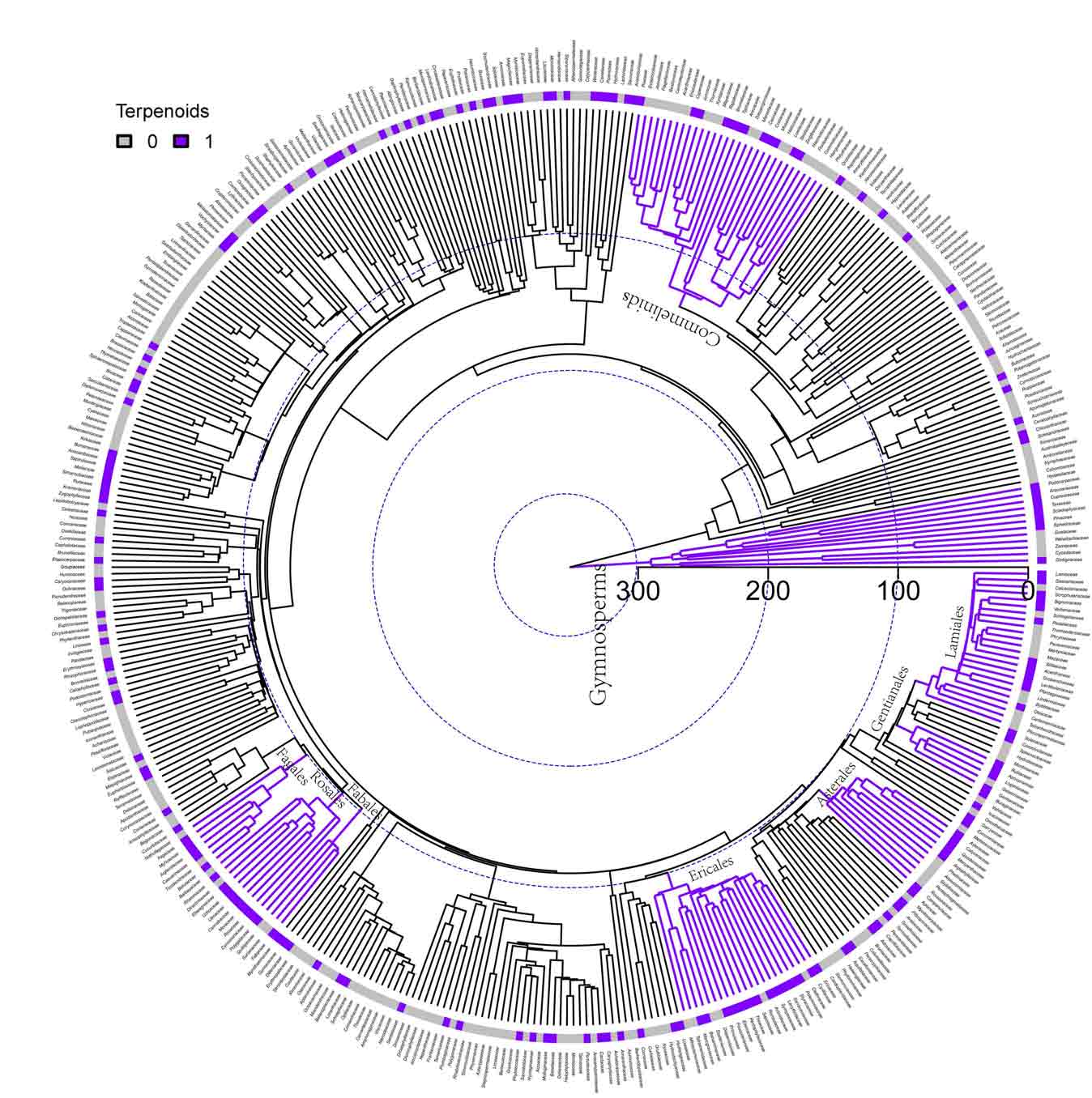


**Fig. S7. Distribution of terpenoids across seed plant phylogeny.** Clades in which terpenoids are determined to be present are colored and labeled.


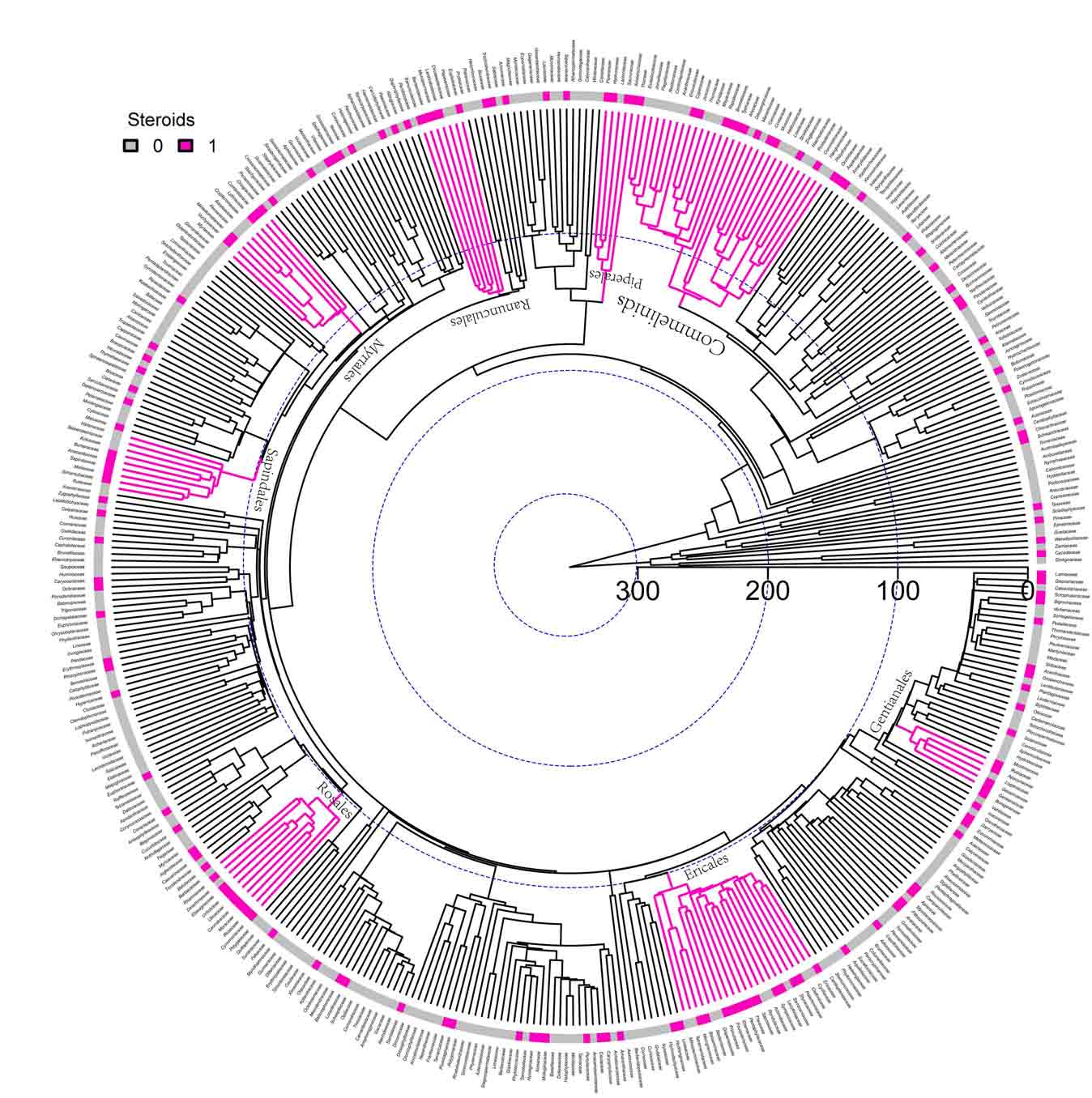


**Fig. S8. Distribution of steroids across seed plant phylogeny.** Clades in which steroids are determined to be present are colored and labeled.

Table S1. Classification of eight class of PSM.

| Alkaloids | Derive from phenylalanine and tyrosine | Phenylalanine group |  |
| --- | --- | --- | --- |
|  |  | Tetrahydrosioquinoline group |  |
|  |  | Benzyltetrahydroisoquinoline | Benzyltetrahydroisoquinoline group |
|  |  |  | Bisbenzyltetrahydroisoquinolines group |
|  |  |  | Proaporphine group |
|  |  |  | Aporphine group |
|  |  |  | Cularine group |
|  |  |  | Pavine group |
|  |  |  | Morphane group |
|  |  |  | Hasubnanane group |
|  |  |  | Erythraline group |
|  |  |  | Protoberberine group |
|  |  |  | Protopine group |
|  |  |  | Phthalideiisoquininoline group |
|  |  |  | Benzophenanthridine group |
|  |  |  | Rhoeadine group |
|  |  |  | Corydaline group |
|  |  | Phenylethyl Tetrahydroisoquinoline group | Phenylethyl Tetrahydroisoquinoline group |
|  |  |  | Bibenylthylisoguinole group |
|  |  |  | Homoproaporphine group |
|  |  |  | Homoaporphine group |
|  |  |  | Homomorphideineone group |
|  |  |  | Colchine group |
|  |  |  | Homoerythrine group |
|  |  |  | Dibens[d,f]azecine group |
|  |  |  | Cephalotane group |
|  |  | Benzylphenylethylamine group | Galarhamine group |
|  |  |  | Lycorine group |
|  |  |  | Haemanthamines group |
|  |  |  | Tazettine group |
|  |  | Emetine group |  |
|  | Derive from ortho-anmino benzoic acid | Simple ortho-benzoic acid alkaloid group |  |
|  |  | Benzodiazepine groupQuiline group |  |
|  |  | Yadirone group |  |
|  | Derive from histidine | Imidazole group |  |
|  | Derive from ornithine | Pyrrolidine group |  |
|  |  | Pyrrolizidine group |  |
|  |  | Tropane group |  |
|  | Derive from lysine | Piperidine group |  |
|  |  | Indolizidine group |  |
|  |  | Quinazoline group |  |
|  | Derive from trytophan | Simple indole alkaloid group |  |
|  |  | Carboline group |  |
|  |  | Semiterpenoid indole group |  |
|  |  | Monoterppenoid indole group | Corynantheine group |
|  |  |  | Strychnine group |
|  |  |  | Aspidospermine group |
|  |  |  | Ibogaine |
|  |  |  | Apparicine group |
|  |  |  | Camptothecine group |
|  |  |  | Cinchonine group |
|  |  | Bisinole group |  |
|  | Terpenoidal alkaloids | monoterppenoid alkaloid group |  |
|  |  | Sesquiterpenoid alkaloid group |  |
|  |  | Diterpenoid alkaloid group |  |
|  |  | Triterpenoid alkaloid group |  |
|  | Steroidal alkaloids | Pregnane alkaloid group |  |
|  |  | Cycloprepnane alkaloid group |  |
|  |  | Cholestane alkaloid group |  |
|  |  | Isocholestane alkaloid group |  |
|  | Derive from nicotinic acid |  |  |
|  | Purines alkaloids |  |  |
|  | Betalains |  |  |
| Phenolic acid | Simple phenolic acid |  |  |
| Phenylpropanoids | Coumarins | Simple coumarin |  |
|  |  | Furo-coumarin |  |
|  |  | Pyrano-coumarin |  |
|  |  | Iso-comarin |  |
|  |  | Poly-coumarin |  |
|  |  | Hybrid Coumarin |  |
|  | Lignans | Simple Lignans | Simple Dibenzylbutane Lignans |
|  |  |  | Dibenzylbytyrolactones Lignans |
|  |  |  | Cyclobutanes Ligans |
|  |  |  | Tetrahyhyrofuranoid Lignans |
|  |  |  | Aryltetralin Lignans |
|  |  |  | Aryltetralin Lactone Lignans |
|  |  |  | Dibencyclooctadiene Lignans |
|  |  | Neolignans |  |
|  |  | Polylignans |  |
|  |  | Norlignans |  |
|  |  | Hybrid Lignans |  |
| Flavonoids | Flavones | Flavones |  |
|  |  | Flavonols |  |
|  | Flavanones | Flavanones |  |
|  |  | Flavanonols |  |
|  | Isoflavones | Isoflavones |  |
|  |  | Isoflavanones |  |
|  | Flavan | Flavanols |  |
|  |  | Isoflavans |  |
|  | Chalcones | Chalcones |  |
|  |  | Dihydrochalcones |  |
|  | Aurones | Aurones |  |
|  |  | Isoaurones |  |
|  | Xanthone |  |  |
|  | Biflavonoids |  |  |
|  | Neoflavanoids |  |  |
|  | Homoflavones |  |  |
|  | Homosioflavones |  |  |
|  | Phenylehtylchromones |  |  |
|  | Anthocyanidins |  |  |
|  | Rotenoids |  |  |
|  | Pterocarpins | flavanonols |  |
| Tannin | Hydrolysable Tannin | Gallotannin |  |
|  |  | Ellagitannin |  |
|  | Condensed Tannin |  |  |
| Quinones | Benzoquinones |  |  |
|  | Naphthoquinones |  |  |
|  | Phenanthraquinones |  |  |
|  | Anthraquinones |  |  |
| Terpenoids | Hemiterpenoids |  |  |
|  | Monoterpenoids |  |  |
|  | Sesquiterpenoids |  |  |
|  | Diterpenoids |  |  |
|  | Seterterpenoids |  |  |
|  | Triterpenoids |  |  |
|  | Tetraterpenoids |  |  |
|  | Polyterpenoids | Normal Carotenoids |  |
|  |  | Norcarotenoids |  |
|  |  | Homocarotenoids |  |
|  | Meroterpenoids |  |  |
| Steroids | Estranes |  |  |
|  | Androstanes |  |  |
|  | Pregnanes |  |  |
|  | Pholanes |  |  |
|  | Cholestanes |  |  |
|  | Stigmastanes |  |  |
|  | Cardenoids | Cardenolides |  |
|  |  | Bufadienolides |  |
|  | Spirostanes | Spirostanes |  |
|  |  | Furo-spirotane |  |
|  | Moulting Hormones |  |  |

Table S2. Original data for PSMs in seed plant.

| Clades of seed plant | Orders of seed plant | Families of seed  plant | Alkaloids | Flavonoids | Phenolic acids | Phenylpropanoids | Quinones | Tannins | Terpenoids | Steroids | No. of PSMs |  |
| --- | --- | --- | --- | --- | --- | --- | --- | --- | --- | --- | --- | --- |
| Gymnosperm |  | Ginkgoaceae |  | √ | √ | √ |  | √ | √ |  | 5 |  |
| Gymnosperm |  | Cycadaceae |  | √ |  | √ |  |  |  | √ | 3 |  |
| Gymnosperm |  | Zamiaceae |  | √ |  |  |  |  |  |  | 1 |  |
| Gymnosperm |  | Welwitschiaceae | √ | √ |  | √ |  |  |  | √ | 4 |  |
| Gymnosperm |  | Gnetaceae | √ | √ | √ | √ |  |  |  |  | 4 |  |
| Gymnosperm |  | Ephedraceae | √ | √ | √ |  |  | √ | √ |  | 5 |  |
| Gymnosperm |  | Pinaceae | √ | √ | √ | √ | √ | √ | √ | √ | 8 |  |
| Gymnosperm |  | Sciadopityaceae |  |  |  |  |  |  | √ |  | 1 |  |
| Gymnosperm |  | Taxaceae |  | √ | √ | √ |  |  | √ | √ | 5 |  |
| Gymnosperm |  | Cupressaceae | √ | √ |  | √ |  | √ | √ |  | 5 |  |
| Gymnosperm |  | Araucariaceae |  | √ |  |  |  |  | √ |  | 2 |  |
| Gymnosperm |  | Podocarpaceae |  | √ | √ |  |  |  | √ |  | 3 |  |
| Basal Angiosperms | Nymphaeales | Hydatellaceae |  |  |  |  |  |  |  |  | 0 |  |
| Basal Angiosperms | Nymphaeales | Cabombaceae |  |  |  |  |  |  |  |  | 0 |  |
| Basal Angiosperms | Nymphaeales | Nymphaeaceae | √ | √ |  |  |  | √ |  |  | 3 |  |
| Basal Angiosperms |  | Amborellaceae |  | √ |  |  |  |  |  |  | 1 |  |
| Basal Angiosperms |  | Austrobaileyaceae |  |  |  | √ |  |  |  |  | 1 |  |
| Basal Angiosperms |  | Trimeniaceae |  |  |  |  |  |  |  |  | 0 |  |
| Basal Angiosperms |  | Schisandraceae |  |  |  | √ |  | √ | √ | √ | 4 |  |
| Basal Angiosperms |  | Chloranthaceae |  | √ | √ | √ | √ |  | √ | √ | 6 |  |
| Basal Angiosperms |  | Ceratophyllaceae |  |  |  |  |  |  | √ |  | 1 |  |
|  |  | Acoraceae |  | √ | √ |  |  |  | √ | √ | 4 |  |
|  | Alismatales | Aponogetonaceae |  |  |  |  |  |  |  |  | 0 |  |
|  | Alismatales | Scheuchzeriaceae |  |  |  | √ |  |  |  |  | 1 |  |
|  | Alismatales | Posidoniaceae |  | √ | √ | √ |  | √ |  |  | 4 |  |
|  | Alismatales | Ruppiaceae |  | √ |  |  |  |  |  |  | 1 |  |
|  | Alismatales | Cymodoceaceae |  |  | √ | √ |  | √ | √ | √ | 5 |  |
|  | Alismatales | Zosteraceae | √ | √ | √ | √ |  | √ |  |  | 5 |  |
|  | Alismatales | Potamogetonaceae | √ |  | √ |  |  |  | √ |  | 3 |  |
|  | Alismatales | Butomaceae |  | √ |  |  |  | √ |  | √ | 3 |  |
|  | Alismatales | Hydrocharitaceae | √ | √ | √ |  |  | √ |  |  | 4 |  |
|  | Alismatales | Juncaginaceae | √ | √ | √ |  |  |  |  |  | 3 |  |
|  | Alismatales | Alismataceae | √ | √ |  | √ | √ | √ | √ | √ | 7 |  |
|  | Alismatales | Tofieldiaceae |  |  |  | √ |  |  |  |  | 1 |  |
|  | Alismatales | Araceae |  | √ |  |  |  | √ |  | √ | 3 |  |
|  |  | Petrosaviaceae |  | √ |  |  |  |  |  |  | 1 |  |
|  | Pandanales | Triuridaceae |  |  |  |  |  |  |  |  | 0 |  |
|  | Pandanales | Stemonaceae | √ | √ |  | √ |  |  |  |  | 3 |  |
|  | Pandanales | Velloziaceae |  | √ |  |  |  | √ |  |  | 2 |  |
|  | Pandanales | Cyclanthaceae |  |  |  |  |  |  |  |  | 0 |  |
|  | Pandanales | Pandanaceae | √ | √ | √ | √ |  | √ | √ | √ | 7 |  |
|  | Dioscoreales | Nartheciaceae |  | √ | √ | √ |  |  |  | √ | 4 |  |
|  | Dioscoreales | Burmanniaceae |  |  |  |  |  |  |  |  | 0 |  |
|  | Dioscoreales | Dioscoreaceae | √ | √ |  |  |  | √ | √ | √ | 5 |  |
|  | Liliales | Corsiaceae |  |  |  |  |  |  |  |  | 0 |  |
|  | Liliales | Campynemataceae |  |  |  |  |  |  |  |  | 0 |  |
|  | Liliales | Petermanniaceae |  |  |  |  |  |  |  |  | 0 |  |
|  | Liliales | Melanthiaceae | √ | √ |  | √ |  | √ |  | √ | 5 |  |
|  | Liliales | Alstroemeriaceae |  | √ |  |  |  |  |  |  | 1 |  |
|  | Liliales | Colchicaceae | √ |  |  |  |  |  |  |  | 1 |  |
| Clades of seed plant | Orders of seed plant | Families of seed  plant | Alkaloids | Flavonoids | Phenolic acids | Phenylpropanoids | Quinones | Tannins | Terpenoids | Steroids | No. of PSMs |  |
|  | Liliales | Smilacaceae |  | √ | √ | √ |  | √ |  | √ | 5 |  |
|  | Liliales | Rhipogonaceae |  |  |  |  |  |  |  |  | 0 |  |
|  | Liliales | Philesiaceae |  |  |  |  |  |  |  |  | 0 |  |
|  | Liliales | Liliaceae | √ | √ | √ | √ | √ | √ | √ | √ | 8 |  |
|  | Asparagales | Boryaceae |  |  |  |  |  |  |  |  | 0 |  |
|  | Asparagales | Blandfordiaceae |  |  |  |  |  |  |  |  | 0 |  |
|  | Asparagales | Asteliaceae |  |  |  |  |  |  |  |  | 0 |  |
|  | Asparagales | Lanariaceae |  |  |  |  |  |  |  |  | 0 |  |
|  | Asparagales | Hypoxidaceae | √ | √ | √ | √ |  |  | √ |  | 5 |  |
|  | Asparagales | Ixioliriaceae |  |  |  |  |  |  |  |  | 0 |  |
|  | Asparagales | Tecophilaeaceae |  |  |  |  |  |  |  |  | 0 |  |
|  | Asparagales | Doryanthaceae |  |  |  |  |  |  |  |  | 0 |  |
|  | Asparagales | Iridaceae | √ | √ | √ | √ | √ | √ | √ | √ | 8 |  |
|  | Asparagales | Xeronemataceae |  |  |  |  |  |  |  |  | 0 |  |
|  | Asparagales | Xanthorrhoeaceae | √ | √ | √ | √ | √ |  |  |  | 5 |  |
|  | Asparagales | Amaryllidaceae | √ | √ | √ | √ |  | √ |  | √ | 6 |  |
|  | Asparagales | Asparagaceae | √ | √ | √ | √ |  | √ | √ | √ | 7 |  |
|  | Asparagales | Orchidaceae | √ | √ | √ | √ | √ | √ |  | √ | 7 |  |
| Commelinids | Commelinales | Philydraceae |  |  | √ |  |  |  |  |  | 1 |  |
| Commelinids | Commelinales | Hanguanaceae |  |  |  |  |  |  |  |  | 0 |  |
| Commelinids | Commelinales | Commelinaceae | √ | √ | √ |  |  | √ |  | √ | 5 |  |
| Commelinids | Commelinales | Pontederiaceae | √ | √ |  | √ |  | √ |  |  | 4 |  |
| Commelinids | Commelinales | Haemodoraceae | √ | √ | √ | √ |  | √ |  |  | 5 |  |
| Commelinids | Zingiberales | Zingiberaceae | √ | √ | √ | √ | √ | √ | √ | √ | 8 |  |
| Commelinids | Zingiberales | Strelitziaceae |  | √ | √ |  |  |  | √ |  | 3 |  |
| Commelinids | Zingiberales | Lowiaceae |  |  |  |  |  |  |  |  | 0 |  |
| Commelinids | Zingiberales | Heliconiaceae | √ | √ |  |  |  |  |  |  | 2 |  |
| Commelinids | Zingiberales | Musaceae | √ | √ | √ | √ |  | √ | √ | √ | 7 |  |
| Commelinids | Zingiberales | Costaceae | √ | √ |  |  |  |  | √ | √ | 4 |  |
| Commelinids | Zingiberales | Cannaceae | √ | √ | √ | √ |  | √ | √ |  | 6 |  |
| Commelinids | Zingiberales | Marantaceae | √ | √ | √ |  |  | √ |  | √ | 5 |  |
| Commelinids |  | Dasypogonaceae |  |  |  |  |  |  |  |  | 0 |  |
| Commelinids |  | Arecaceae | √ | √ | √ | √ |  | √ | √ | √ | 7 |  |
| Commelinids | Poales | Typhaceae | √ | √ | √ |  |  | √ | √ | √ | 6 |  |
| Commelinids | Poales | Bromeliaceae | √ | √ | √ | √ |  | √ | √ | √ | 7 |  |
| Commelinids | Poales | Rapateaceae | √ | √ | √ | √ |  | √ | √ | √ | 7 |  |
| Commelinids | Poales | Mayacaceae |  | √ |  |  |  |  |  |  | 1 |  |
| Commelinids | Poales | Xyridaceae |  | √ |  | √ | √ | √ |  |  | 4 |  |
| Commelinids | Poales | Thurniaceae |  |  |  | √ |  | √ |  |  | 2 |  |
| Commelinids | Poales | Juncaceae |  | √ | √ | √ | √ |  | √ | √ | 6 |  |
| Commelinids | Poales | Cyperaceae | √ | √ | √ | √ | √ | √ | √ | √ | 8 |  |
| Commelinids | Poales | Eriocaulaceae | √ | √ | √ | √ |  |  |  |  | 4 |  |
| Commelinids | Poales | Anarthriaceae |  |  |  |  |  |  |  |  | 0 |  |
| Commelinids | Poales | Centrolepidaceae |  |  |  |  |  |  |  |  | 0 |  |
| Commelinids | Poales | Restionaceae | √ | √ |  |  |  |  |  |  | 2 |  |
| Commelinids | Poales | Flagellariaceae |  | √ | √ |  |  | √ |  |  | 3 |  |
| Commelinids | Poales | Joinvilleaceae |  |  |  |  |  |  |  |  | 0 |  |
| Commelinids | Poales | Ecdeiocoleaceae |  |  |  |  |  |  |  |  | 0 |  |
| Commelinids | Poales | Poaceae | √ | √ | √ | √ | √ | √ | √ | √ | 8 |  |
| Magnoliids | Piperales | Aristolochiaceae | √ | √ |  | √ |  |  | √ | √ | 5 |  |
| Magnoliids | Piperales | Saururaceae | √ | √ | √ | √ | √ | √ | √ | √ | 8 |  |
| Magnoliids | Piperales | Lactoridaceae |  | √ |  |  |  |  |  |  | 1 |  |
| Magnoliids | Piperales | Hydnoraceae | √ | √ |  | √ |  | √ | √ | √ | 6 |  |
| Magnoliids | Piperales | Piperaceae | √ | √ | √ | √ |  | √ | √ | √ | 7 |  |
| Magnoliids |  | Canellaceae | √ |  |  |  |  |  | √ |  | 2 |  |
| Magnoliids |  | Winteraceae | √ | √ |  |  |  | √ | √ |  | 4 |  |
| Magnoliids | Laurales | Calycanthaceae | √ | √ |  |  |  |  |  |  | 2 |  |
| Magnoliids | Laurales | Gomortegaceae |  |  |  | √ |  |  |  |  | 1 |  |
| Magnoliids | Laurales | Atherospermataceae | √ |  |  |  |  |  |  |  | 1 |  |
| Clades of seed plant | Orders of seed plant | Families of seed  plant | Alkaloids | Flavonoids | Phenolic acids | Phenylpropanoids | Quinones | Tannins | Terpenoids | Steroids | No. of PSMs |  |
| Magnoliids | Laurales | Siparunaceae | √ | √ |  |  |  |  | √ | √ | 4 |  |
| Magnoliids | Laurales | Hernandiaceae | √ |  |  | √ | √ |  |  |  | 3 |  |
| Magnoliids | Laurales | Monimiaceae | √ | √ |  | √ |  | √ | √ |  | 5 |  |
| Magnoliids | Laurales | Lauraceae | √ | √ | √ | √ |  | √ | √ | √ | 7 |  |
| Magnoliids | Magnoliales | Himantandraceae | √ | √ |  |  |  |  |  |  | 2 |  |
| Magnoliids | Magnoliales | Degeneriaceae |  |  |  |  |  |  |  |  | 0 |  |
| Magnoliids | Magnoliales | Eupomatiaceae | √ | √ |  |  |  |  |  |  | 2 |  |
| Magnoliids | Magnoliales | Myristicaceae | √ | √ |  | √ |  |  | √ |  | 4 |  |
| Magnoliids | Magnoliales | Magnoliaceae | √ | √ |  | √ | √ | √ | √ |  | 6 |  |
| Magnoliids | Magnoliales | Annonaceae | √ | √ |  |  | √ | √ | √ | √ | 6 |  |
|  | Proteales | Sabiaceae | √ |  |  |  |  | √ |  |  | 2 |  |
|  | Proteales | Trochodendraceae |  | √ | √ | √ |  | √ | √ | √ | 6 |  |
|  | Proteales | Buxaceae | √ | √ | √ |  | √ |  | √ | √ | 6 |  |
|  | Proteales | Nelumbonaceae | √ | √ |  |  |  |  |  |  | 2 |  |
|  | Proteales | Platanaceae | √ | √ | √ |  |  | √ | √ |  | 5 |  |
|  | Proteales | Proteaceae | √ | √ | √ |  | √ | √ |  |  | 5 |  |
|  | Ranunculales | Eupteleaceae |  | √ |  |  | √ |  | √ | √ | 4 |  |
|  | Ranunculales | Papaveraceae | √ | √ |  | √ |  | √ |  |  | 4 |  |
|  | Ranunculales | Circaeasteraceae |  |  |  |  |  |  |  |  | 0 |  |
|  | Ranunculales | Lardizabalaceae |  | √ | √ | √ |  | √ | √ | √ | 6 |  |
|  | Ranunculales | Menispermaceae | √ | √ |  | √ |  | √ | √ | √ | 6 |  |
|  | Ranunculales | Berberidaceae | √ | √ |  | √ | √ | √ |  | √ | 6 |  |
|  | Ranunculales | Ranunculaceae | √ | √ | √ | √ | √ | √ | √ | √ | 8 |  |
|  | Saxifragales | Peridiscaceae |  |  |  |  |  |  |  |  | 0 |  |
|  | Saxifragales | Daphniphyllaceae | √ | √ | √ |  |  | √ | √ | √ | 6 |  |
|  | Saxifragales | Altingiaceae |  |  |  |  |  |  |  |  | 0 |  |
|  | Saxifragales | Paeoniaceae | √ | √ | √ |  |  |  | √ | √ | 5 |  |
|  | Saxifragales | Cercidiphyllaceae |  | √ | √ |  |  | √ |  |  | 3 |  |
|  | Saxifragales | Hamamelidaceae | √ | √ | √ | √ |  | √ | √ | √ | 7 |  |
|  | Saxifragales | Tetracarpaeaceae |  |  |  |  |  |  |  |  | 0 |  |
|  | Saxifragales | Aphanopetalaceae |  |  |  |  |  |  |  |  | 0 |  |
|  | Saxifragales | Penthoraceae |  | √ | √ |  |  | √ |  |  | 3 |  |
|  | Saxifragales | Haloragaceae | √ | √ |  |  |  | √ |  |  | 3 |  |
|  | Saxifragales | Crassulaceae | √ | √ | √ | √ | √ | √ | √ | √ | 8 |  |
|  | Saxifragales | Iteaceae |  |  |  |  |  |  |  |  | 0 |  |
|  | Saxifragales | Grossulariaceae | √ | √ | √ |  |  | √ | √ | √ | 6 |  |
|  | Saxifragales | Saxifragaceae | √ | √ | √ | √ | √ | √ | √ | √ | 8 |  |
|  |  | Vitaceae | √ | √ | √ | √ |  | √ | √ | √ | 7 |  |
| Malvids | Geraniales | Melianthaceae |  | √ |  |  |  | √ |  |  | 2 |  |
| Malvids | Geraniales | Vivianiaceae |  |  |  |  |  |  |  |  | 0 |  |
| Malvids | Geraniales | Geraniaceae | √ | √ | √ | √ |  | √ | √ | √ | 7 |  |
| Malvids | Crossosomatales | Aphloiaceae |  |  |  |  |  |  |  |  | 0 |  |
| Malvids | Crossosomatales | Geissolomataceae |  |  |  |  |  |  |  |  | 0 |  |
| Malvids | Crossosomatales | Strasburgeriaceae |  |  |  |  |  |  |  |  | 0 |  |
| Malvids | Crossosomatales | Staphyleaceae | √ | √ |  |  |  | √ | √ |  | 4 |  |
| Malvids | Crossosomatales | Guamatelaceae |  |  |  |  |  |  |  |  | 0 |  |
| Malvids | Crossosomatales | Crossosomataceae | √ |  |  | √ |  |  |  |  | 2 |  |
| Malvids | Crossosomatales | Stachyuraceae |  |  |  |  |  | √ |  | √ | 2 |  |
| Malvids |  | Picramniaceae |  |  |  |  | √ |  |  |  | 1 |  |
| Malvids | Myrtales | Onagraceae | √ | √ | √ | √ |  | √ | √ | √ | 7 |  |
| Malvids | Myrtales | Combretaceae | √ | √ | √ | √ | √ | √ | √ | √ | 8 |  |
| Malvids | Myrtales | Lythraceae | √ | √ |  | √ | √ | √ | √ | √ | 7 |  |
| Malvids | Myrtales | Crypteroniaceae |  |  |  |  |  |  |  |  | 0 |  |
| Malvids | Myrtales | Alzateaceae |  |  |  |  |  |  |  |  | 0 |  |
| Malvids | Myrtales | Penaeaceae |  |  |  |  |  |  |  |  | 0 |  |
| Malvids | Myrtales | Melastomataceae | √ | √ | √ | √ | √ | √ | √ | √ | 8 |  |
| Malvids | Myrtales | Vochysiaceae | √ | √ |  |  | √ | √ | √ | √ | 6 |  |
| Malvids | Myrtales | Myrtaceae | √ | √ | √ | √ |  | √ | √ |  | 6 |  |
| Malvids |  | Gerrardinaceae |  |  |  |  |  |  |  |  | 0 |  |
| Clades of seed plant | Orders of seed plant | Families of seed  plant | Alkaloids | Flavonoids | Phenolic acids | Phenylpropanoids | Quinones | Tannins | Terpenoids | Steroids | No. of PSMs |  |
| Malvids |  | Dipentodontaceae |  |  |  |  |  |  |  |  | 0 |  |
| Malvids |  | Tapisciaceae |  |  |  |  |  |  |  |  | 0 |  |
| Malvids | Brassicales | Limnanthaceae | √ |  |  |  |  | √ |  |  | 2 |  |
| Malvids | Brassicales | Setchellanthaceae |  |  |  |  |  |  |  |  | 0 |  |
| Malvids | Brassicales | Emblingiaceae |  |  |  |  |  |  |  |  | 0 |  |
| Malvids | Brassicales | Tovariaceae | √ |  |  |  |  |  |  |  | 1 |  |
| Malvids | Brassicales | Pentadiplandraceae | √ |  |  |  |  |  |  |  | 1 |  |
| Malvids | Brassicales | Gyrostemonaceae | √ | √ |  |  |  |  |  |  | 2 |  |
| Malvids | Brassicales | Resedaceae | √ | √ | √ |  |  |  |  | √ | 4 |  |
| Malvids | Brassicales | Koeberliniaceae |  |  |  |  |  |  |  |  | 0 |  |
| Malvids | Brassicales | Bataceae |  |  |  |  |  |  |  |  | 0 |  |
| Malvids | Brassicales | Salvadoraceae | √ | √ |  |  |  | √ |  |  | 3 |  |
| Malvids | Brassicales | Moringaceae |  | √ |  |  |  |  | √ |  | 2 |  |
| Malvids | Brassicales | Caricaceae | √ | √ | √ | √ |  | √ |  |  | 5 |  |
| Malvids | Brassicales | Akaniaceae |  |  |  |  |  |  |  |  | 0 |  |
| Malvids | Brassicales | Tropaeolaceae |  | √ | √ |  |  | √ |  |  | 3 |  |
| Malvids | Brassicales | Capparaceae | √ | √ | √ | √ |  | √ | √ | √ | 7 |  |
| Malvids | Brassicales | Cleomaceae | √ |  |  |  |  |  | √ |  | 2 |  |
| Malvids | Brassicales | Brassicaceae | √ | √ | √ | √ |  | √ | √ | √ | 7 |  |
| Malvids | Malvales | Neuradaceae |  | √ |  |  |  |  |  |  | 1 |  |
| Malvids | Malvales | Thymelaeaceae | √ | √ | √ | √ |  | √ | √ | √ | 7 |  |
| Malvids | Malvales | Sphaerosepalaceae |  |  |  |  |  |  |  |  | 0 |  |
| Malvids | Malvales | Bixaceae | √ |  |  |  |  | √ | √ |  | 3 |  |
| Malvids | Malvales | Cistaceae |  | √ | √ | √ |  | √ | √ | √ | 6 |  |
| Malvids | Malvales | Sarcolaenaceae |  |  |  |  |  |  |  |  | 0 |  |
| Malvids | Malvales | Dipterocarpaceae | √ | √ | √ | √ | √ | √ | √ | √ | 8 |  |
| Malvids | Malvales | Petenaeaceae |  |  |  |  |  |  |  |  | 0 |  |
| Malvids | Malvales | Muntingiaceae |  |  |  |  |  |  |  |  | 0 |  |
| Malvids | Malvales | Cytinaceae |  |  |  |  |  |  |  |  | 0 |  |
| Malvids | Malvales | Malvaceae | √ | √ | √ | √ | √ | √ |  | √ | 7 |  |
| Malvids | Sapindales | Nitrariaceae | √ | √ |  |  |  |  |  |  | 2 |  |
| Malvids | Sapindales | Biebersteiniaceae | √ | √ |  |  |  | √ |  |  | 3 |  |
| Malvids | Sapindales | Kirkiaceae |  |  |  |  |  |  |  |  | 0 |  |
| Malvids | Sapindales | Burseraceae | √ | √ | √ | √ |  | √ | √ | √ | 7 |  |
| Malvids | Sapindales | Anacardiaceae | √ | √ | √ | √ | √ | √ | √ | √ | 8 |  |
| Malvids | Sapindales | Sapindaceae | √ | √ | √ | √ | √ | √ | √ | √ | 8 |  |
| Malvids | Sapindales | Meliaceae | √ | √ |  | √ | √ | √ | √ | √ | 7 |  |
| Malvids | Sapindales | Simaroubaceae | √ | √ | √ | √ | √ | √ | √ | √ | 8 |  |
| Malvids | Sapindales | Rutaceae | √ | √ | √ | √ | √ | √ | √ |  | 7 |  |
|  |  | Krameriaceae |  | √ | √ | √ |  | √ | √ |  | 5 |  |
|  |  | Zygophyllaceae | √ | √ | √ | √ | √ | √ | √ | √ | 8 |  |
| Fabids |  | Lepidobotryaceae |  |  |  |  |  |  | √ |  | 1 |  |
| Fabids |  | Celastraceae | √ | √ | √ | √ | √ | √ | √ | √ | 8 |  |
| Fabids | Oxalidales | Huaceae |  |  |  |  |  |  |  |  | 0 |  |
| Fabids | Oxalidales | Connaraceae |  | √ |  |  |  | √ |  |  | 2 |  |
| Fabids | Oxalidales | Oxalidaceae | √ | √ |  |  |  | √ |  |  | 3 |  |
| Fabids | Oxalidales | Cunoniaceae | √ | √ |  |  |  | √ | √ | √ | 5 |  |
| Fabids | Oxalidales | Cephalotaceae | √ | √ | √ |  |  | √ |  |  | 4 |  |
| Fabids | Oxalidales | Brunelliaceae |  |  |  |  |  |  | √ |  | 1 |  |
| Fabids | Oxalidales | Elaeocarpaceae | √ | √ | √ |  |  | √ | √ |  | 5 |  |
| Fabids | Malpighiales | Goupiaceae |  |  |  |  |  |  |  |  | 0 |  |
| Fabids | Malpighiales | Humiriaceae | √ |  |  |  |  | √ |  |  | 2 |  |
| Fabids | Malpighiales | Caryocaraceae |  | √ | √ |  |  |  | √ | √ | 4 |  |
| Fabids | Malpighiales | Ochnaceae | √ | √ | √ | √ |  | √ | √ | √ | 7 |  |
| Fabids | Malpighiales | Picrodendraceae |  |  |  |  |  |  |  |  | 0 |  |
| Fabids | Malpighiales | Balanopaceae |  |  |  |  |  |  | √ |  | 1 |  |
| Fabids | Malpighiales | Trigoniaceae | √ |  |  |  |  |  |  |  | 1 |  |
| Fabids | Malpighiales | Dichapetalaceae | √ | √ |  |  | √ | √ | √ | √ | 6 |  |
| Fabids | Malpighiales | Euphroniaceae |  |  |  |  |  |  |  |  | 0 |  |
| Clades of seed plant | Orders of seed plant | Families of seed  plant | Alkaloids | Flavonoids | Phenolic acids | Phenylpropanoids | Quinones | Tannins | Terpenoids | Steroids | No. of PSMs |  |
| Fabids | Malpighiales | Chrysobalanaceae |  | √ |  |  |  |  | √ |  | 2 |  |
| Fabids | Malpighiales | Phyllanthaceae |  |  |  | √ |  | √ |  |  | 2 |  |
| Fabids | Malpighiales | Linaceae | √ | √ | √ | √ |  | √ | √ |  | 6 |  |
| Fabids | Malpighiales | Irvingiaceae |  |  | √ |  |  |  |  |  | 1 |  |
| Fabids | Malpighiales | Pandaceae | √ |  |  |  |  |  |  |  | 1 |  |
| Fabids | Malpighiales | Erythroxylaceae | √ | √ | √ | √ |  | √ | √ | √ | 7 |  |
| Fabids | Malpighiales | Rhizophoraceae | √ | √ | √ | √ | √ | √ | √ | √ | 8 |  |
| Fabids | Malpighiales | Bonnetiaceae |  |  |  |  |  |  |  |  | 0 |  |
| Fabids | Malpighiales | Calophyllaceae |  | √ |  | √ |  |  | √ |  | 3 |  |
| Fabids | Malpighiales | Podostemaceae | √ | √ |  |  |  |  |  |  | 2 |  |
| Fabids | Malpighiales | Hypericaceae | √ | √ | √ |  | √ | √ | √ | √ | 7 |  |
| Fabids | Malpighiales | Clusiaceae | √ | √ | √ | √ | √ | √ | √ |  | 7 |  |
| Fabids | Malpighiales | Ctenolophonaceae |  |  |  |  |  |  |  |  | 0 |  |
| Fabids | Malpighiales | Lophopyxidaceae |  |  |  |  |  |  |  |  | 0 |  |
| Fabids | Malpighiales | Putranjivaceae |  |  |  | √ |  |  | √ | √ | 3 |  |
| Fabids | Malpighiales | Ixonanthaceae |  |  |  |  |  |  | √ |  | 1 |  |
| Fabids | Malpighiales | Achariaceae |  |  |  |  |  |  |  |  | 0 |  |
| Fabids | Malpighiales | Passifloraceae | √ | √ |  | √ |  |  |  |  | 3 |  |
| Fabids | Malpighiales | Violaceae | √ | √ |  |  |  |  |  |  | 2 |  |
| Fabids | Malpighiales | Lacistemataceae |  |  |  |  |  |  |  |  | 0 |  |
| Fabids | Malpighiales | Salicaceae | √ | √ | √ | √ |  | √ | √ |  | 6 |  |
| Fabids | Malpighiales | Elatinaceae |  |  |  |  |  |  | √ |  | 1 |  |
| Fabids | Malpighiales | Malpighiaceae | √ | √ | √ |  |  |  | √ |  | 4 |  |
| Fabids | Malpighiales | Euphorbiaceae | √ | √ | √ | √ | √ | √ | √ | √ | 8 |  |
| Fabids | Malpighiales | Rafflesiaceae | √ |  |  | √ |  | √ |  |  | 3 |  |
| Fabids | Cucurbitales | Tetramelaceae |  |  |  |  |  |  | √ |  | 1 |  |
| Fabids | Cucurbitales | Datiscaceae |  | √ |  |  |  |  |  |  | 1 |  |
| Fabids | Cucurbitales | Apodanthaceae |  |  |  |  |  |  |  |  | 0 |  |
| Fabids | Cucurbitales | Corynocarpaceae |  |  |  |  |  | √ |  |  | 1 |  |
| Fabids | Cucurbitales | Coriariaceae | √ |  |  |  |  |  | √ | √ | 3 |  |
| Fabids | Cucurbitales | Anisophylleaceae |  |  | √ |  |  |  | √ |  | 2 |  |
| Fabids | Cucurbitales | Begoniaceae |  | √ |  |  |  | √ |  |  | 2 |  |
| Fabids | Cucurbitales | Cucurbitaceae | √ | √ | √ | √ |  | √ | √ | √ | 7 |  |
| Fabids | Fagales | Nothofagaceae |  | √ | √ |  |  |  |  |  | 2 |  |
| Fabids | Fagales | Fagaceae | √ | √ | √ | √ |  | √ | √ |  | 6 |  |
| Fabids | Fagales | Myricaceae | √ | √ | √ |  |  | √ | √ |  | 5 |  |
| Fabids | Fagales | Juglandaceae | √ | √ | √ | √ | √ | √ | √ | √ | 8 |  |
| Fabids | Fagales | Casuarinaceae | √ | √ | √ |  |  | √ | √ | √ | 6 |  |
| Fabids | Fagales | Ticodendraceae |  |  |  |  |  |  |  |  | 0 |  |
| Fabids | Fagales | Betulaceae | √ | √ | √ | √ |  | √ | √ | √ | 7 |  |
| Fabids | Rosales | Barbeyaceae |  | √ | √ |  |  |  |  |  | 2 |  |
| Fabids | Rosales | Rhamnaceae | √ | √ | √ | √ | √ | √ | √ | √ | 8 |  |
| Fabids | Rosales | Dirachmaceae |  |  |  |  |  |  |  |  | 0 |  |
| Fabids | Rosales | Elaeagnaceae | √ | √ | √ | √ |  | √ | √ | √ | 7 |  |
| Fabids | Rosales | Urticaceae | √ | √ | √ | √ | √ | √ | √ | √ | 8 |  |
| Fabids | Rosales | Ulmaceae | √ | √ | √ | √ |  | √ | √ | √ | 7 |  |
| Fabids | Rosales | Cannabaceae | √ | √ | √ | √ | √ |  | √ | √ | 7 |  |
| Fabids | Rosales | Moraceae | √ | √ | √ | √ |  | √ | √ | √ | 7 |  |
| Fabids | Rosales | Rosaceae | √ | √ | √ | √ | √ | √ | √ | √ | 8 |  |
| Fabids | Rosales | Cynomoriaceae | √ | √ | √ | √ |  |  | √ | √ | 6 |  |
| Fabids | Fabales | Polygalaceae | √ | √ | √ | √ |  | √ | √ |  | 6 |  |
| Fabids | Fabales | Quillajaceae |  |  |  |  |  |  | √ |  | 1 |  |
| Fabids | Fabales | Surianaceae |  |  |  |  |  |  | √ |  | 1 |  |
| Fabids | Fabales | Fabaceae | √ | √ | √ | √ | √ | √ | √ | √ | 8 |  |
|  |  | Myrothamnaceae |  | √ | √ |  |  |  | √ |  | 3 |  |
|  |  | Gunneraceae | √ | √ |  |  |  | √ | √ |  | 4 |  |
|  |  | Dilleniaceae | √ | √ | √ |  |  | √ | √ |  | 5 |  |
|  | Santalales | Erythropalaceae |  |  |  |  |  |  |  |  | 0 |  |
|  | Santalales | Strombosiaceae |  |  |  |  |  |  |  |  | 0 |  |
|  | Santalales | Coulaceae |  |  |  |  |  |  |  |  | 0 |  |
| Clades of seed plant | Orders of seed plant | Families of seed  plant | Alkaloids | Flavonoids | Phenolic acids | Phenylpropanoids | Quinones | Tannins | Terpenoids | Steroids | No. of PSMs |  |
|  | Santalales | Ximeniaceae |  |  |  |  |  |  |  |  | 0 |  |
|  | Santalales | Olacaceae | √ | √ |  | √ |  | √ | √ | √ | 6 |  |
|  | Santalales | Aptandraceae |  |  |  |  |  |  |  |  | 0 |  |
|  | Santalales | Octoknemaceae |  |  |  |  |  |  |  |  | 0 |  |
|  | Santalales | Misodendraceae |  |  |  |  |  |  |  |  | 0 |  |
|  | Santalales | Balanophoraceae |  | √ |  | √ |  | √ | √ | √ | 5 |  |
|  | Santalales | Loranthaceae | √ | √ | √ | √ |  | √ | √ | √ | 7 |  |
|  | Santalales | Schoepfiaceae |  |  |  |  |  |  |  |  | 0 |  |
|  | Santalales | Opiliaceae |  |  |  | √ |  |  |  |  | 1 |  |
|  | Santalales | Comandraceae |  |  |  |  |  |  |  |  | 0 |  |
|  | Santalales | Thesiaceae |  |  |  |  |  |  |  |  | 0 |  |
|  | Santalales | Cervantesiaceae |  |  |  |  |  |  |  |  | 0 |  |
|  | Santalales | Amphorogynaceae |  |  |  |  |  |  |  |  | 0 |  |
|  | Santalales | Viscaceae |  |  |  |  |  |  |  |  | 0 |  |
|  | Santalales | Nanodeaceae |  |  |  |  |  |  |  |  | 0 |  |
|  | Santalales | Santalaceae | √ | √ | √ | √ |  |  | √ | √ | 6 |  |
|  | Caryophyllales | Droseraceae | √ | √ | √ |  | √ | √ |  |  | 5 |  |
|  | Caryophyllales | Drosophyllaceae |  |  |  |  |  |  |  |  | 0 |  |
|  | Caryophyllales | Dioncophyllaceae | √ |  |  |  |  |  |  |  | 1 |  |
|  | Caryophyllales | Ancistrocladaceae | √ |  |  |  | √ |  |  |  | 2 |  |
|  | Caryophyllales | Nepenthaceae |  | √ |  |  | √ | √ |  |  | 3 |  |
|  | Caryophyllales | Frankeniaceae | √ | √ |  |  |  |  |  |  | 2 |  |
|  | Caryophyllales | Tamaricaceae | √ | √ | √ | √ |  | √ | √ | √ | 7 |  |
|  | Caryophyllales | Plumbaginaceae | √ | √ |  |  | √ | √ |  | √ | 5 |  |
|  | Caryophyllales | Polygonaceae | √ | √ | √ | √ | √ | √ | √ |  | 7 |  |
|  | Caryophyllales | Rhabdodendraceae |  |  |  |  |  |  | √ |  | 1 |  |
|  | Caryophyllales | Simmondsiaceae |  | √ |  |  |  |  |  |  | 1 |  |
|  | Caryophyllales | Physenaceae |  | √ |  |  |  |  | √ |  | 2 |  |
|  | Caryophyllales | Asteropeiaceae |  |  |  |  |  |  |  |  | 0 |  |
|  | Caryophyllales | Stegnospermataceae |  |  |  |  |  |  |  | √ | 1 |  |
|  | Caryophyllales | Limeaceae |  |  |  |  |  |  |  |  | 0 |  |
|  | Caryophyllales | Barbeuiaceae |  |  |  |  |  |  |  |  | 0 |  |
|  | Caryophyllales | Gisekiaceae |  |  |  |  |  |  |  |  | 0 |  |
|  | Caryophyllales | Phytolaccaceae | √ | √ |  | √ |  |  | √ | √ | 5 |  |
|  | Caryophyllales | Sarcobataceae |  |  |  |  |  |  |  |  | 0 |  |
|  | Caryophyllales | Nyctaginaceae | √ | √ | √ |  |  | √ | √ | √ | 6 |  |
|  | Caryophyllales | Aizoaceae | √ | √ |  | √ |  | √ |  | √ | 5 |  |
|  | Caryophyllales | Molluginaceae |  | √ |  |  |  |  | √ | √ | 3 |  |
|  | Caryophyllales | Basellaceae | √ | √ |  |  |  |  | √ |  | 3 |  |
|  | Caryophyllales | Didiereaceae | √ | √ |  |  |  |  |  |  | 2 |  |
|  | Caryophyllales | Halophytaceae |  |  |  |  |  |  |  |  | 0 |  |
|  | Caryophyllales | Montiaceae |  |  |  |  |  |  |  |  | 0 |  |
|  | Caryophyllales | Talinaceae |  |  |  |  |  |  |  |  | 0 |  |
|  | Caryophyllales | Portulacaceae | √ | √ | √ | √ | √ |  | √ | √ | 7 |  |
|  | Caryophyllales | Anacampserotaceae |  |  |  |  |  |  |  |  | 0 |  |
|  | Caryophyllales | Cactaceae | √ | √ | √ | √ |  |  | √ | √ | 6 |  |
|  | Caryophyllales | Caryophyllaceae | √ | √ |  | √ | √ | √ | √ | √ | 7 |  |
|  | Caryophyllales | Achatocarpaceae |  |  |  |  |  |  |  |  | 0 |  |
|  | Caryophyllales | Amaranthaceae | √ | √ | √ |  | √ |  | √ | √ | 6 |  |
|  |  | Aextoxicaceae |  |  |  |  |  |  |  |  | 0 |  |
|  |  | Berberidopsidaceae |  |  |  |  |  |  |  |  | 0 |  |
|  | Cornales | Cornaceae | √ | √ | √ | √ |  | √ | √ |  | 6 |  |
|  | Cornales | Curtisiaceae |  |  |  |  |  |  |  |  | 0 |  |
|  | Cornales | Grubbiaceae |  |  |  |  |  |  |  |  | 0 |  |
|  | Cornales | Nyssaceae | √ |  |  |  |  |  | √ |  | 2 |  |
|  | Cornales | Hydrostachyaceae |  |  |  |  |  |  |  |  | 0 |  |
|  | Cornales | Hydrangeaceae | √ | √ |  |  |  | √ | √ | √ | 5 |  |
| Clades of seed plant | Orders of seed plant | Families of seed  plant | Alkaloids | Flavonoids | Phenolic acids | Phenylpropanoids | Quinones | Tannins | Terpenoids | Steroids | No. of PSMs |  |
|  | Cornales | Loasaceae | √ |  |  |  |  |  | √ | √ | 3 |  |
|  | Ericales | Mitrastemonaceae |  |  |  |  |  |  |  |  | 0 |  |
|  | Ericales | Tetrameristaceae |  |  |  |  |  | √ |  |  | 1 |  |
|  | Ericales | Marcgraviaceae |  | √ | √ |  |  |  | √ | √ | 4 |  |
|  | Ericales | Balsaminaceae | √ | √ | √ | √ | √ | √ | √ | √ | 8 |  |
|  | Ericales | Sladeniaceae |  |  |  |  |  |  |  |  | 0 |  |
|  | Ericales | Diapensiaceae | √ |  |  |  |  | √ |  |  | 2 |  |
|  | Ericales | Primulaceae | √ | √ | √ | √ | √ | √ | √ | √ | 8 |  |
|  | Ericales | Fouquieriaceae |  | √ |  |  |  |  | √ | √ | 3 |  |
|  | Ericales | Ebenaceae | √ | √ | √ | √ | √ | √ | √ | √ | 8 |  |
|  | Ericales | Pentaphylacaceae |  |  |  |  |  | √ | √ | √ | 3 |  |
|  | Ericales | Theaceae | √ | √ | √ | √ | √ | √ | √ | √ | 8 |  |
|  | Ericales | Sapotaceae | √ | √ | √ | √ | √ | √ | √ | √ | 8 |  |
|  | Ericales | Roridulaceae |  |  |  |  |  |  |  |  | 0 |  |
|  | Ericales | Actinidiaceae | √ | √ | √ | √ | √ | √ | √ |  | 7 |  |
|  | Ericales | Symplocaceae | √ | √ | √ | √ |  |  | √ | √ | 6 |  |
|  | Ericales | Lecythidaceae | √ | √ | √ |  | √ | √ | √ | √ | 7 |  |
|  | Ericales | Sarraceniaceae | √ | √ | √ |  |  | √ | √ |  | 5 |  |
|  | Ericales | Styracaceae | √ | √ | √ | √ |  |  | √ | √ | 6 |  |
|  | Ericales | Polemoniaceae | √ | √ | √ | √ | √ |  | √ | √ | 7 |  |
|  | Ericales | Clethraceae |  |  |  |  |  | √ | √ |  | 2 |  |
|  | Ericales | Cyrillaceae |  |  |  |  |  | √ | √ |  | 2 |  |
|  | Ericales | Ericaceae | √ | √ | √ | √ | √ | √ | √ | √ | 8 |  |
| Campanulids | Aquifoliales | Cardiopteridaceae |  |  |  |  |  |  | √ |  | 1 |  |
| Campanulids | Aquifoliales | Stemonuraceae |  |  |  |  |  |  |  |  | 0 |  |
| Campanulids | Aquifoliales | Phyllonomaceae |  |  |  |  |  |  |  |  | 0 |  |
| Campanulids | Aquifoliales | Helwingiaceae |  |  |  |  |  |  |  |  | 0 |  |
| Campanulids | Aquifoliales | Aquifoliaceae | √ | √ | √ | √ | √ | √ | √ | √ | 8 |  |
| Campanulids |  | Escalloniaceae | √ | √ |  |  |  |  | √ |  | 3 |  |
| Campanulids |  | Paracryphiaceae |  |  |  |  |  |  |  |  | 0 |  |
| Campanulids |  | Columelliaceae |  |  |  |  |  |  |  |  | 0 |  |
| Campanulids |  | Bruniaceae | √ |  |  |  |  |  |  |  | 1 |  |
| Campanulids |  | Adoxaceae | √ | √ | √ | √ |  |  | √ |  | 4 |  |
| Campanulids |  | Caprifoliaceae | √ | √ | √ | √ |  | √ | √ | √ | 7 |  |
| Campanulids | Apiales | Pennantiaceae |  |  |  |  |  |  |  |  | 0 |  |
| Campanulids | Apiales | Torricelliaceae |  |  |  |  |  |  | √ |  | 1 |  |
| Campanulids | Apiales | Griseliniaceae |  |  |  |  |  |  |  |  | 0 |  |
| Campanulids | Apiales | Araliaceae | √ | √ | √ | √ |  |  | √ | √ | 6 |  |
| Campanulids | Apiales | Pittosporaceae | √ | √ | √ | √ | √ | √ | √ | √ | 8 |  |
| Campanulids | Apiales | Myodocarpaceae |  |  |  |  |  |  |  |  | 0 |  |
| Campanulids | Apiales | Apiaceae | √ | √ | √ | √ | √ | √ | √ | √ | 8 |  |
| Campanulids | Asterales | Campanulaceae | √ | √ | √ | √ |  | √ | √ | √ | 7 |  |
| Campanulids | Asterales | Rousseaceae |  |  |  |  |  |  |  |  | 0 |  |
| Campanulids | Asterales | Pentaphragmataceae |  |  |  |  |  |  |  |  | 0 |  |
| Campanulids | Asterales | Stylidiaceae |  |  |  |  |  |  |  |  | 0 |  |
| Campanulids | Asterales | Alseuosmiaceae |  |  |  |  |  |  |  |  | 0 |  |
| Campanulids | Asterales | Phellinaceae |  |  |  |  |  |  |  |  | 0 |  |
| Campanulids | Asterales | Argophyllaceae |  | √ | √ |  |  | √ | √ |  | 4 |  |
| Campanulids | Asterales | Menyanthaceae | √ | √ |  |  |  |  | √ |  | 3 |  |
| Campanulids | Asterales | Goodeniaceae | √ |  |  |  |  |  | √ |  | 2 |  |
| Campanulids | Asterales | Calyceraceae |  | √ | √ |  |  |  | √ |  | 3 |  |
| Campanulids | Asterales | Asteraceae | √ | √ | √ | √ | √ | √ | √ | √ | 8 |  |
|  |  | Metteniusaceae |  |  |  |  |  |  |  |  | 0 |  |
| Lamiids |  | Eucommiaceae | √ | √ | √ | √ |  |  | √ | √ | 6 |  |
| Lamiids |  | Garryaceae | √ |  |  |  |  |  | √ | √ | 3 |  |
| Lamiids |  | Oncothecaceae |  |  |  |  |  |  |  |  | 0 |  |
| Lamiids |  | Icacinaceae | √ | √ | √ | √ |  | √ | √ | √ | 7 |  |
| Lamiids |  | Vahliaceae |  |  |  |  |  |  | √ |  | 1 |  |
| Lamiids |  | Boraginaceae | √ | √ | √ |  | √ |  | √ | √ | 6 |  |
| Clades of seed plant | Orders of seed plant | Families of seed  plant | Alkaloids | Flavonoids | Phenolic acids | Phenylpropanoids | Quinones | Tannins | Terpenoids | Steroids | No. of PSMs |  |
| Lamiids | Gentianales | Gentianaceae | √ | √ | √ | √ | √ | √ | √ | √ | 8 |  |
| Lamiids | Gentianales | Gelsemiaceae | √ |  |  |  |  |  |  | √ | 2 |  |
| Lamiids | Gentianales | Loganiaceae | √ | √ | √ | √ | √ |  | √ |  | 6 |  |
| Lamiids | Gentianales | Apocynaceae | √ | √ | √ | √ | √ | √ | √ | √ | 8 |  |
| Lamiids | Gentianales | Rubiaceae | √ | √ | √ | √ | √ | √ | √ | √ | 8 |  |
| Lamiids | Solanales | Montiniaceae |  |  |  |  |  |  |  |  | 0 |  |
| Lamiids | Solanales | Hydroleaceae |  |  |  |  |  |  |  |  | 0 |  |
| Lamiids | Solanales | Sphenocleaceae | √ |  |  |  |  |  |  |  | 1 |  |
| Lamiids | Solanales | Convolvulaceae | √ | √ | √ | √ |  |  | √ |  | 5 |  |
| Lamiids | Solanales | Solanaceae | √ | √ | √ | √ | √ | √ | √ | √ | 8 |  |
| Lamiids | Lamiales | Plocospermataceae |  |  |  |  |  |  |  |  | 0 |  |
| Lamiids | Lamiales | Tetrachondraceae |  |  |  |  |  |  |  |  | 0 |  |
| Lamiids | Lamiales | Carlemanniaceae |  |  |  |  |  |  |  |  | 0 |  |
| Lamiids | Lamiales | Oleaceae | √ | √ | √ | √ |  | √ | √ | √ | 7 |  |
| Lamiids | Lamiales | Byblidaceae |  |  |  |  |  |  |  |  | 0 |  |
| Lamiids | Lamiales | Linderniaceae |  |  |  |  |  |  |  |  | 0 |  |
| Lamiids | Lamiales | Plantaginaceae | √ | √ | √ | √ |  | √ | √ | √ | 7 |  |
| Lamiids | Lamiales | Lentibulariaceae | √ | √ |  | √ |  |  | √ |  | 4 |  |
| Lamiids | Lamiales | Orobanchaceae | √ | √ | √ | √ | √ |  | √ | √ | 7 |  |
| Lamiids | Lamiales | Acanthaceae | √ | √ | √ | √ | √ |  | √ | √ | 7 |  |
| Lamiids | Lamiales | Stilbaceae | √ |  |  |  |  |  | √ |  | 2 |  |
| Lamiids | Lamiales | Mazaceae |  |  |  |  |  |  |  |  | 0 |  |
| Lamiids | Lamiales | Martyniaceae |  |  |  |  |  |  |  |  | 0 |  |
| Lamiids | Lamiales | Paulowniaceae | √ | √ | √ |  |  |  | √ |  | 4 |  |
| Lamiids | Lamiales | Phrymaceae | √ |  |  | √ |  |  |  |  | 2 |  |
| Lamiids | Lamiales | Thomandersiaceae |  |  |  |  |  |  |  |  | 0 |  |
| Lamiids | Lamiales | Pedaliaceae | √ | √ | √ | √ |  |  | √ | √ | 6 |  |
| Lamiids | Lamiales | Schlegeliaceae |  |  |  |  |  |  |  |  | 0 |  |
| Lamiids | Lamiales | Verbenaceae | √ | √ | √ | √ | √ |  | √ |  | 6 |  |
| Lamiids | Lamiales | Bignoniaceae | √ | √ | √ | √ | √ | √ | √ | √ | 8 |  |
| Lamiids | Lamiales | Scrophulariaceae | √ | √ |  | √ | √ | √ | √ | √ | 7 |  |
| Lamiids | Lamiales | Calceolariaceae | √ | √ |  |  |  |  | √ |  | 3 |  |
| Lamiids | Lamiales | Gesneriaceae | √ | √ | √ | √ | √ |  | √ | √ | 7 |  |
| Lamiids | Lamiales | Lamiaceae | √ | √ | √ | √ | √ | √ | √ | √ | 8 |  |

**‘√’means certain PSM present in the family.**

Table S3. Original results for D values in each major seed plant clade tested.

| Clade of seed plant | PSMs | D-value | *p* value (Random shuffle) | *p* value (Brownian motion) | Number of permutations |
| --- | --- | --- | --- | --- | --- |
| Asterids | Alkaloids | 0.751 | 0.068 | 0.021** | 1000 |
|  | Phenolic acids | 0.872 | 0.252 | 0.009** | 1000 |
|  | Flavonoids | 0.959 | 0.406 | 0.006** | 1000 |
|  | Phenylpropanoids | 1.024 | 0.536 | <0.001** | 1000 |
|  | Quinones | 0.574 | 0.038* | 0.125 | 1000 |
|  | Tannins | 0.796 | 0.159 | 0.018** | 1000 |
|  | Terpenoids | 0.641 | 0.034* | 0.058 | 1000 |
|  | Steroids | 0.943 | 0.367 | 0.002** | 1000 |
|  |  |  |  |  |  |
| Basal eudicots | Alkaloids | 0.891 | 0.441 | 0.272 | 1000 |
|  | Phenolic acids | 0.408 | 0.230 | 0.418 | 1000 |
|  | Flavonoids | 1.221 | 0.536 | 0.195 | 1000 |
|  | Phenylpropanoids | 0.372 | 0.188 | 0.402 | 1000 |
|  | Quinones | 0.893 | 0.435 | 0.247 | 1000 |
|  | Tannins | 0.608 | 0.270 | 0.325 | 1000 |
|  | Terpenoids | 1.865 | 0.908 | 0.024** | 1000 |
|  | Steroids | 0.026 | 0.100 | 0.528 | 1000 |
|  |  |  |  |  |  |
| Campanulids | Alkaloids | 1.664 | 0.668 | 0.178 | 1000 |
|  | Phenolic acids | 1.016 | 0.466 | 0.295 | 1000 |
|  | Flavonoids | 1.102 | 0.483 | 0.299 | 1000 |
|  | Phenylpropanoids | 0.446 | 0.342 | 0.459 | 1000 |
|  | Quinones | 6.446 | 0.925 | 0.034** | 1000 |
|  | Tannins | 4.453 | 0.970 | 0.013** | 1000 |
|  | Terpenoids | 0.459 | 0.300 | 0.426 | 1000 |
|  | Steroids | 2.035 | 0.718 | 0.168 | 1000 |
|  |  |  |  |  |  |
| Caryophyllales | Alkaloids | 0.850 | 0.368 | 0.195 | 1000 |
|  | Phenolic acids | 2.127 | 0.916 | 0.028** | 1000 |
|  | Flavonoids | 1.203 | 0.596 | 0.104 | 1000 |
|  | Phenylpropanoids | 1.511 | 0.735 | 0.087 | 1000 |
|  | Quinones | -0.389 | 0.044* | 0.677 | 1000 |
|  | Tannins | -0.313 | 0.056 | 0.642 | 1000 |
|  | Terpenoids | 1.666 | 0.829 | 0.028** | 1000 |
|  | Steroids | 1.242 | 0.615 | 0.104 | 1000 |
|  |  |  |  |  |  |
| Commelinids | Alkaloids | 1.034 | 0.486 | 0.172 | 1000 |
|  | Phenolic acids | 1.223 | 0.608 | 0.083 | 1000 |
|  | Flavonoids | 1.844 | 0.858 | 0.030** | 1000 |
|  | Phenylpropanoids | 0.426 | 0.177 | 0.385 | 1000 |
|  | Quinones | 1.089 | 0.510 | 0.213 | 1000 |
|  | Tannins | 1.000 | 0.474 | 0.164 | 1000 |
|  | Terpenoids | 0.576 | 0.245 | 0.328 | 1000 |
|  | Steroids | 0.773 | 0.339 | 0.252 | 1000 |
|  |  |  |  |  |  |
| Fabids | Alkaloids | 1.037 | 0.526 | 0.059 | 1000 |
|  | Phenolic acids | -0.081 | 0.015* | 0.566 | 1000 |
|  | Flavonoids | 0.479 | 0.144 | 0.262 | 1000 |
|  | Phenylpropanoids | 0.218 | 0.055 | 0.405 | 1000 |
|  | Quinones | 1.327 | 0.666 | 0.073 | 1000 |
|  | Tannins | 1.160 | 0.616 | 0.035** | 1000 |
|  | Terpenoids | 1.083 | 0.542 | 0.047** | 1000 |
|  | Steroids | 0.690 | 0.269 | 0.193 | 1000 |
| Clade of seed plant | PSMs | D-value | *p* value (Random shuffle) | *p* value (Brownian motion) | Number of permutations |
| Lamiids | Alkaloids | 0.425 | 0.210 | 0.392 | 1000 |
|  | Phenolic acids | 1.314 | 0.642 | 0.108 | 1000 |
|  | Flavonoids | 0.655 | 0.295 | 0.298 | 1000 |
|  | Phenylpropanoids | 1.354 | 0.663 | 0.094 | 1000 |
|  | Quinones | 0.034 | 0.116 | 0.548 | 1000 |
|  | Tannins | 1.734 | 0.788 | 0.060 | 1000 |
|  | Terpenoids | 0.873 | 0.411 | 0.248 | 1000 |
|  | Steroids | 1.210 | 0.600 | 0.120 | 1000 |
|  |  |  |  |  |  |
| Malpighiales | Alkaloids | -0.106 | 0.242 | 0.544 | 1000 |
|  | Phenolic acids | -1.638 | 0.044* | 0.831 | 1000 |
|  | Flavonoids | -1.112 | 0.120 | 0.737 | 1000 |
|  | Phenylpropanoids | 0.107 | 0.244 | 0.512 | 1000 |
|  | Quinones | 1.585 | 0.556 | 0.330 | 1000 |
|  | Tannins | -0.107 | 0.250 | 0.557 | 1000 |
|  | Terpenoids | 1.340 | 0.549 | 0.218 | 1000 |
|  | Steroids | 0.427 | 0.352 | 0.452 | 1000 |
| Malvids | Alkaloids | 0.150 | 0.032* | 0.438 | 1000 |
|  | Phenolic acids | 0.856 | 0.345 | 0.106 | 1000 |
|  | Flavonoids | 0.079 | 0.016* | 0.451 | 1000 |
|  | Phenylpropanoids | 0.336 | 0.078 | 0.330 | 1000 |
|  | Quinones | -0.775 | 0.002* | 0.815 | 1000 |
|  | Tannins | 0.367 | 0.078 | 0.303 | 1000 |
|  | Terpenoids | -0.407 | 0.008* | 0.728 | 1000 |
|  | Steroids | 0.832 | 0.334 | 0.092 | 1000 |
|  |  |  |  |  |  |
| Monocots | Alkaloids | 1.091 | 0.598 | 0.012** | 1000 |
|  | Phenolic acids | 0.910 | 0.354 | 0.024** | 1000 |
|  | Flavonoids | 1.114 | 0.637 | 0.010** | 1000 |
|  | Phenylpropanoids | 0.955 | 0.423 | 0.033** | 1000 |
|  | Quinones | 0.814 | 0.348 | 0.148 | 1000 |
|  | Tannins | 0.977 | 0.455 | 0.017** | 1000 |
|  | Terpenoids | 1.318 | 0.809 | 0.002** | 1000 |
|  | Steroids | 1.142 | 0.649 | 0.008** | 1000 |
|  |  |  |  |  |  |
| Nitrogen-fixing clade | Alkaloids | 0.953 | 0.441 | 0.147 | 1000 |
|  | Phenolic acids | 0.227 | 0.133 | 0.441 | 1000 |
|  | Flavonoids | 0.824 | 0.360 | 0.194 | 1000 |
|  | Phenylpropanoids | 0.385 | 0.155 | 0.372 | 1000 |
|  | Quinones | 1.269 | 0.606 | 0.116 | 1000 |
|  | Tannins | 1.785 | 0.927 | 0.010** | 1000 |
|  | Terpenoids | 1.418 | 0.736 | 0.049** | 1000 |
|  | Steroids | 1.101 | 0.545 | 0.104 | 1000 |
|  |  |  |  |  |  |
| Rosids | Alkaloids | 0.715 | 0.141 | 0.037** | 1000 |
|  | Phenolic acids | 0.324 | 0.015* | 0.256 | 1000 |
|  | Flavonoids | 0.362 | 0.016* | 0.197 | 1000 |
|  | Phenylpropanoids | 0.352 | 0.021* | 0.211 | 1000 |
|  | Quinones | 0.432 | 0.076 | 0.243 | 1000 |
|  | Tannins | 0.785 | 0.211 | 0.024** | 1000 |
|  | Terpenoids | 0.292 | 0.002* | 0.258 | 1000 |
|  | Steroids | 0.808 | 0.258 | 0.024** | 1000 |
|  |  |  |  |  |  |
| Clade of seed plant | PSMs | D-value | *p* value (Random shuffle) | *p* value (Brownian motion) | Number of permutations |
| Superrosids | Alkaloids | 0.793 | 0.206 | 0.024** | 1000 |
|  | Phenolic acids | 0.413 | 0.022* | 0.170 | 1000 |
|  | Flavonoids | 0.418 | 0.021* | 0.154 | 1000 |
|  | Phenylpropanoids | 0.374 | 0.015* | 0.202 | 1000 |
|  | Quinones | 0.424 | 0.069* | 0.223 | 1000 |
|  | Tannins | 0.702 | 0.130 | 0.034** | 1000 |
|  | Terpenoids | 0.297 | 0.006* | 0.242 | 1000 |
|  | Steroids | 0.811 | 0.257 | 0.029** | 1000 |
|  |  |  |  |  |  |
| Superasterids | Alkaloids | 0.733 | 0.083 | 0.031** | 1000 |
|  | Phenolic acids | 0.851 | 0.211 | 0.008** | 1000 |
|  | Flavonoids | 0.971 | 0.430 | 0.006** | 1000 |
|  | Phenylpropanoids | 1.038 | 0.571 | 0.002** | 1000 |
|  | Quinones | 0.582 | 0.044* | 0.106 | 1000 |
|  | Tannins | 0.781 | 0.137 | 0.036** | 1000 |
|  | Terpenoids | 0.649 | 0.027* | 0.057 | 1000 |
|  | Steroids | 0.951 | 0.372 | 0.003** | 1000 |
|  |  |  |  |  |  |
| Eudicots | Alkaloids | 0.713 | 0.017* | 0.002** | 1000 |
|  | Phenolic acids | 0.688 | 0.015* | 0.004** | 1000 |
|  | Flavonoids | 0.691 | 0.017* | 0.004** | 1000 |
|  | Phenylpropanoids | 0.760 | 0.045* | 0.001** | 1000 |
|  | Quinones | 0.646 | 0.016* | 0.016** | 1000 |
|  | Tannins | 0.656 | 0.006* | 0.009** | 1000 |
|  | Terpenoids | 0.657 | 0.006* | 0.004** | 1000 |
|  | Steroids | 0.870 | 0.184 | <0.001** | 1000 |
|  |  |  |  |  |  |
|  | Alkaloids | 0.800 | 0.094 | 0.002** | 1000 |
| Core eudicots | Phenolic acids | 0.708 | 0.026* | 0.005** | 1000 |
|  | Flavonoids | 0.749 | 0.038* | 0.001** | 1000 |
|  | Phenylpropanoids | 0.786 | 0.075 | <0.001** | 1000 |
|  | Quinones | 0.591 | 0.011* | 0.032** | 1000 |
|  | Tannins | 0.643 | 0.015* | 0.007** | 1000 |
|  | Terpenoids | 0.555 | 0.001* | 0.017** | 1000 |
|  | Steroids | 0.916 | 0.276 | <0.001** | 1000 |
|  |  |  |  |  |  |
| Angiosperm | Alkaloids | 0.785 | 0.025* | <0.001** | 1000 |
|  | Phenolic acids | 0.756 | 0.015* | <0.001** | 1000 |
|  | Flavonoids | 0.788 | 0.024* | <0.001** | 1000 |
|  | Phenylpropanoids | 0.836 | 0.068 | 0.001** | 1000 |
|  | Quinones | 0.703 | 0.015* | 0.004** | 1000 |
|  | Tannins | 0.787 | 0.027* | 0.001** | 1000 |
|  | Terpenoids | 0.696 | 0.003* | <0.001** | 1000 |
|  | Steroids | 0.941 | 0.291 | 0.001** | 1000 |
|  |  |  |  |  |  |
| Seed plant | Alkaloids | 0.785 | 0.016* | 0.001** | 1000 |
|  | Phenolic acids | 0.789 | 0.028* | <0.001** | 1000 |
|  | Flavonoids | 0.763 | 0.014* | <0.001** | 1000 |
|  | Phenylpropanoids | 0.826 | 0.063 | <0.001** | 1000 |
|  | Quinones | 0.694 | 0.005* | <0.001** | 1000 |
|  | Tannins | 0.798 | 0.036* | <0.001** | 1000 |
|  | Terpenoids | 0.672 | 0.002* | 0.001** | 1000 |
|  | Steroids | 0.976 | 0.400 | <0.001** | 1000 |

* *p__Rand_*_om_ < 0.05 means corresponding D value is statistically significant. ** D value is statistically significant under Brownian motion model (*p*__Brownian motion_ < 0.05).Table S4. Original sources for PSM data

| **Families and PSMs acquired from publisher** | **Article or book source** |
| --- | --- |
| Acanthaceae | (1-8) |
| Achariaceae | (9-11) |
| Adoxaceae | (12-15) |
| Aizoaceae | (16, 17) |
| Akaniaceae | (18) |
| Alismataceae | (19) |
| Alstroemeriaceae | (20, 21) |
| Amaranthaceae | (22-30) |
| Amaryllidaceae | (31-33) |
| Anacardiaceae | (34-37) |
| Ancistrocladaceae | (38-40) |
| Annonaceae | (41-51) |
| Apiaceae | (52-54) |
| Apocynaceae | (55-57) |
| Aquifoliaceae | (58-60) |
| Araceae | (61-63) |
| Araliaceae | (64-68) |
| Araucariaceae | (69, 70) |
| Arecaceae | (71-80) |
| Argophyllaceae | (81) |
| Aristolochiaceae | (82-84) |
| Asparagaceae | (85-88) |
| Asteraceae | (89-92) |
| Balanophoraceae | (93-97) |
| Basellaceae | (98-101) |
| Betulaceae | (102-106) |
| Biebersteiniaceae | (107, 108) |
| Bignoniaceae | (109-113) |
| Boraginaceae | (114-118) |
| Brassicaceae | (119-124) |
| Bromeliaceae | (125-129) |
| Burseraceae | (130-132) |
| Buxaceae | (133-136) |
| Cactaceae | (137-141) |
| Calophyllaceae | (142-144) |
| Calyceraceae | (145, 146) |
| Campanulaceae | (147-149) |
| Cannabaceae | (150-153) |
| Capparaceae | (154-158) |
| Caprifoliaceae | (159-162) |
| Caricaceae | (163-165) |
| Caryocaraceae | (166-168) |
| Celastraceae | (169-173) |
| Cephalotaceae | (174) |
| Cercidiphyllaceae | (175, 176) |
| Chloranthaceae | (177-185) |
| Chrysobalanaceae | (186) |
| Cistaceae | (187-190) |
| Guttiferae | (191-195) |
| Colchicaceae | (196-199) |
| Combretaceae | (200) |
| Commelinaceae | (127, 201, 202) |
| Convolvulaceae | (203-210) |
| Coriariaceae | (211-213) |
| Cornaceae | (214-216) |
| Costaceae | (217) |
| Crassulaceae | (218-221) |
| Cucurbitaceae | (222) |
| Cupressaceae | (223-226) |
| Cycadaceae | (227-229) |
| Cymodoceaceae | (230, 231) |
| Cynomoriaceae | (232, 233) |
| Cyperaceae | (234) |
| Cyrillaceae | (235) |
| Daphniphyllaceae | (236-241) |
| Dichapetalaceae | (242, 243) |
| Didiereaceae | (244, 245) |
| Dilleniaceae | (246, 247) |
| Dioncophyllaceae | (39, 248) |
| Dioscoreaceae | (249-257) |
| Dipterocarpaceae | (258-266) |
| Droseraceae | (267-269) |
| Ebenaceae | (270) |
| Elaeagnaceae | (271-276) |
| Elaeocarpaceae | (277-281) |
| Ephedraceae | (282-284) |
| Ericaceae | (285, 286) |
| Ericales | (286) |
| Eriocaulaceae | (287-291) |
| Erythroxylaceae | (292-302) |
| Escalloniaceae | (303) |
| Eucommiaceae | (304-306) |
| Euphorbiaceae | (307-311) |
| Eupomatiaceae | (312) |
| Eupteleaceae | (313, 314) |
| Fabaceae | (315-318) |
| Fagaceae | (272, 319-322) |
| Gelsemiaceae | (323) |
| Gentianaceae | (324-333) |
| Geraniaceae | (334-338) |
| Gesneriaceae | (339-342) |
| Ginkgoaceae | (343-345) |
| Gnetaceae | (346-352) |
| Gramineae | (353) |
| Grossulariaceae | (354-357) |
| Gunneraceae | (358) |
| gymnosperm | (70, 359) |
| Haemodoraceae | (360-365) |
| Haloragaceae | (358) |
| Hamamelidaceae | (366) |
| Hernandiaceae | (367) |
| Humiriaceae | (368) |
| Hydrangeaceae | (369) |
| Hydrocharitaceae | (32, 318, 370) |
| Hydrostachyaceae | (371) |
| Hypericaceae | (372-377) |
| Hypoxidaceae | (378-382) |
| Icacinaceae | (383-386) |
| Iridaceae | (387) |
| Juglandaceae | (388-394) |
| Juncaginaceae | (395) |
| Krameriaceae | (396, 397) |
| Lactoridaceae | (398) |
| Lamiaceae | (399-403) |
| Lardizabalaceae | (404-408) |
| Lauraceae | (409-414) |
| Lecythidaceae | (415-418) |
| Lentibulariaceae | (419) |
| Liliaceae | (127, 420, 421) |
| Linaceae | (422-428) |
| Loasaceae | (429-431) |
| Loganiaceae | (432-434) |
| Loranthaceae | (435) |
| Lythraceae | (436, 437) |
| Magnoliaceae | (438-440) |
| Malpighiaceae | (441-445) |
| Malvaceae | (446-451) |
| Marantaceae | (452) |
| Marcgraviaceae | (453) |
| Melanthiaceae | (454-456) |
| Melastomataceae | (457-463) |
| Melianthaceae | (464) |
| Menispermaceae | (465-469) |
| Menyanthaceae | (470) |
| Molluginaceae | (471, 472) |
| Monimiaceae | (473) |
| Moraceae | (217, 474) |
| Musaceae | (475-477) |
| Myricaceae | (478-484) |
| Myristicaceae | (485-489) |
| Myrothamnaceae | (176) |
| Myrtaceae | (490-496) |
| Nartheciaceae | (497, 498) |
| Nelumbonaceae | (499) |
| Nepenthaceae | (500) |
| Neuradaceae | (501) |
| Nitrariaceae | (502, 503) |
| Nothofagaceae | (504, 505) |
| Nyctaginaceae | (506-508) |
| Nymphaeaceae | (509, 510) |
| Ochnaceae | (511-523) |
| Olacaceae | (524-528) |
| Oleaceae | (529-533) |
| Onagraceae | (534) |
| Orchidaceae | (535-537) |
| Orobanchaceae | (538-544) |
| Oxalidaceae | (545, 546) |
| Pandanaceae | (547-551) |
| Paeoniaceae | (552) |
| Papaveraceae | (553-560) |
| Passifloraceae | (561-564) |
| Pentaphylacaceae | (565) |
| Penthoraceae | (566) |
| Petrosaviaceae | (567) |
| Phrymaceae | (568) |
| Phyllanthaceae | (569) (570, 571) |
| Phytolaccaceae | (572-577) |
| Pinaceae | (578-580) |
| Piperaceae | (581-584) |
| Pittosporaceae | (585, 586) |
| Plantaginaceae | (587-591) |
| Platanaceae | (592, 593) |
| Plumbaginaceae | (594-596) |
| Podostemaceae | (597) |
| Polemoniaceae | (598, 599) |
| Polygalaceae | (600-607) |
| Polypodiaceae | (608) |
| Pontederiaceae | (127, 350) |
| Portulacaceae | (609-615) |
| Posidoniaceae | (230, 231, 616-619) |
| Potamogetonaceae | (620-622) |
| Primulaceae | (623, 624) |
| Proteaceae | (625-628) |
| Rafflesiaceae | (629) |
| Ranunculaceae | (55, 630-633) |
| Resedaceae | (634-637) |
| Rhamnaceae | (638-640) |
| Rhizophoraceae | (641-647) |
| Rosaceae | (272, 648-666) |
| Rubiaceae | (667-670) |
| Rutaceae | (671-683) |
| Sabiaceae | (684) |
| Salicaceae | (685-691) |
| Salvadoraceae | (692) |
| Santalaceae | (693-698) |
| Sapindaceae | (699-709) |
| Sarraceniaceae | (57, 710) |
| Saururaceae | (711-713) |
| Schisandraceae | (714, 715) |
| Scrophulariaceae | (541, 716-721) |
| Simaroubaceae | (722-725) |
| Siparunaceae | (726-728) |
| Smilacaceae | (729-735) |
| Solanaceae | (88, 736-741) |
| Staphyleaceae | (742) |
| Stemonaceae | (743-750) |
| Strelitziaceae | (751) |
| Styracaceae | (752-755) |
| Symplocaceae | (756-758) |
| Tamaricaceae | (759-761) |
| Taxaceae | (762-768) |
| Thymelaeaceae | (769-773) |
| Tropaeolaceae | (774) |
| Typhaceae | (127, 775) |
| Ulmaceae | (776) |
| Urticaceae | (777-781) |
| Verbenaceae | (782-785) |
| Vitaceae | (350, 786) |
| Vochysiaceae | (787-789) |
| Welwitschiaceae | (350) |
| Winteraceae | (790, 791) |
| Xanthorrhoeaceae | (792-796) |
| Xyridaceae | (797-799) |
| Zingiberaceae | (800, 801) |
| Zosteraceae | (231, 802) |
| Zygophyllaceae | (803-810) |
| Alkaloids | (39, 233, 253, 811-888) |
| Flavonoids | (32, 34, 51, 57, 69, 74, 75, 82, 105, 128, 216, 261, 262, 267, 296, 298, 330, 364, 366, 412, 413, 415, 437, 443, 448, 463, 498, 501, 505, 512-515, 532, 567, 597, 611, 624, 634, 635, 656, 678, 695, 702, 732, 745, 791, 805, 823, 836, 888-921) |
| Phenolic acid | (11, 30, 35, 72, 73, 104, 164, 167, 291, 322, 354, 387, 388, 461, 495, 520, 610, 619, 651, 658, 659, 686, 688-690, 698, 752, 796, 836, 915, 922-936) |
| L&C | (207, 397, 422-425, 571, 671, 674-676, 682, 808, 888, 898, 937-961) |
| Quinones | (649, 888, 898, 962-974) |
| Steroids | (249-252, 256, 454, 456, 733-735, 741, 804, 836, 888, 898, 975-980) |
| Tannin | (392, 629, 759, 760, 836, 888, 898, 981-993) |
| Terpenoids | (151, 152, 223, 330, 386, 580, 836, 888, 994-1008) |
|  |  |

**Selected reference:**

1. Huo C-H, Wang B, Lin W-H *et al.* Benzoxazinones from *Acanthus ilicifolius*. *Biochem Syst Ecol* 2005; **33**(6):643-645. doi: 10.1016/j.bse.2004.11.002.

2. Wang Y, Luo SH, Hua J *et al.* Capitate glandular trichomes of *Paragutzlaffia henryi* harbor new phytotoxic labdane diterpenoids. *J Agric Food Chem* 2015; **63**(45):10004-12. doi: 10.1021/acs.jafc.5b04113.

3. Kanchanapoom T, Noiarsa P, Otsuka H *et al.* Chemical constituents of *Acanthus volubilis* Wall. *Biochem Syst Ecol*2006; **34**(5):442-445. doi: 10.1016/j.bse.2005.12.006.

4. Sophie Susplugas NVH, Jerome Bignon, Odile Thoison *et al.* Cytotoxic arylnaphthalene lignans from a Vietnamese Acanthaceae, *Justicia patentiflora*. *J. Nat. Prod.* 2005; **68**:734-738.

5. Tripetch Kanchanapoom MSK, Ryoji Kasai, Kazuo Yamasaki CP *et al.* Lignan glucosides from *Acanthus ilicifolius*. *Phytochemistry* 2001; **56**:369±372.

6. Wu J, Zhang S, Xiao Q *et al.* Phenylethanoid and aliphatic alcohol glycosides from *Acanthus ilicifolius*. *Phytochemistry* 2003; **63**(4):491-495. doi: 10.1016/s0031-9422(03)00100-6.

7. Ghasemzadeh A, Jaafar HZ, Rahmat A*.* Phytochemical constituents and biological activities of different extracts of *Strobilanthes crispus* (L.) Bremek leaves grown in different locations of Malaysia. *BMC Complement Altern Med* 2015*.* **15**(1):422. doi: 10.1186/s12906-015-0873-3.

8. Anna Sendl JLC, S. D. Jolad, Cheryl Stoddart *et al.* Two new naphthoquinones with antiviral activity from *Rhinacanthus nasutus*. *J. Nat. Prod.* 1996; **59**:808-811.

9. Webber BL, Miller RE, Woodrow IE*.* Constitutive polymorphic cyanogenesis in the Australian rainforest tree, *Ryparosa kurrangii* (Achariaceae). *Phytochemistry* 2007; **68**(15):2068-74. doi: 10.1016/j.phytochem.2007.04.038.

10. Webber BL, Miller RE*.*  Gynocardin from *Baileyoxylon lanceolatum* and a revision of cyanogenic glycosides in Achariaceae. *Biochem Syst Ecol*2008; **36**(7):545-553. doi: 10.1016/j.bse.2008.03.011.

11. Liu L, Guo Z, Chai X *et al.* Phenolic glycosides from the stems of *Homalium ceylanicum* (Gardner) Bentham (Flacourtiaceae/Salicaceae sensu lato). *Biochem Syst Ecol*2013; **46**:55-58. doi: 10.1016/j.bse.2012.09.006.

12. Annelise Lobstein GH-A, Jurgen Englert, Jean-Georges Kuhry *et al.* Chemotaxonomical investigation in the genus *Viburnum*. *Phytochemistry* 1999; **50**:1175-1180.

13. Pieri V, Schwaiger S, Ellmerer EP *et al.* Iridoid glycosides from the leaves of *Sambucus ebulus*. *J Nat Prod* 2009; **72**(10):1798-803. doi: 10.1021/np900373u.

14. Mohamed MA, Marzouk MS, Moharram FA *et al.* Phytochemical constituents and hepatoprotective activity of *Viburnum tinus*. *Phytochemistry* 2005; **66**(23):2780-6. doi: 10.1016/j.phytochem.2005.07.019.

15. Atay İ, Kirmizibekmez H, GÖRen AC *et al.* Secondary metabolites from *Sambucus ebulus*. *Turkish Journal of Chemistry* 2015; **39**:34-41. doi: 10.3906/kim-1403-47.

16. Satyajit D. Sarker TS, Pensri Whiting *et al.* Ecdysteroids from the seeds of *Trianthema turgidifolia* and *T. pilosa* (Aizoaceae). *Biochem Syst Ecol*1998; **26**:691-693.

17. Satyajit D. Sarker VS, Laurence Dinan. Isoamericanin A: a neolignan from *Trianthema turgidifolia*. *Biochem Syst Ecol* 1998; **26**:681-683.

18. Montaut S, Zhang WD, Nuzillard JM *et al.* Glucosinolate Diversity in Bretschneidera sinensis of Chinese Origin. *J Nat Prod* 2015; **78**(8):2001-6. doi: 10.1021/acs.jnatprod.5b00338.

19. Zheng X-W. Chemical analysis of diterpenoids in *Caldesia grandis* and its chemotaxonomic implication. *Acta Phytotaxonomica Sinica* 2007; **45**(04):570. doi: 10.1360/aps06086.

20. Tatsuzawa F, Saito N, Murata N *et al.* 6-Hydroxypelargonidin glycosides in the orange–red flowers of Alstroemeria. *Phytochemistry* 2003; **62**(8):1239-1242. doi: 10.1016/s0031-9422(02)00683-0.

21. Rikke Norbaek LPC, Gustav Bojesen Karsten Brandt. Anthocyanins in chilean species of *Alstroemeria* (Alstroemeriaceae). *Phytochemistry* 1996; **42**(1):97-100.

22. Gerald Blunden M-hY, Gabor Janicsak, Imre Mathe *et al.* Betaine distribution in the Amaranthaceae. *Biochem Syst Ecol* 1999; **27**:87-92.

23. Tamara Savchenko PW, Satyajit D. Sarker, Laurence Dinan. Distribution and identity of phytoecdysteroids in *Gomphrena* spp. (Amaranthaceae). *Biochem Syst Ecol* 1998; **26**:337-346.

24. Salvador MJ, Dias DA. Flavone C-glycosides from *Alternanthera maritima* (Mart.) St. Hil. (Amaranthaceae). *Biochem Syst Ecol* 2004; **32**(1):107-110. doi: 10.1016/s0305-1978(03)00180-7.

25. Ferreira EdO, Dias DA. Phytochemical investigation of *Gomphrena claussenii* Moq. *Biochem Syst Ecol* 2004; **32**(9):823-827. doi: 10.1016/j.bse.2004.02.001.

26. E.de O. Ferreira DAD. Phytochemical investigation of *Gomphrena claussenii* Moq. *Biochem Syst Ecol* 2004; **32**:823-827. doi: 10.5923/j.ijmb.20150501.02.

27. Mroczek A. Phytochemistry and bioactivity of triterpene saponins from Amaranthaceae family. *Phytochemistry Reviews* 2015; **14**(4):577-605. doi: 10.1007/s11101-015-9394-4.

28. Hirayama Y, Okuzumi K, Masubuti H *et al.* Stereochemical assignment of C-24 and C-25 of amarasterone A, a putative biosynthetic intermediate of cyasterone. *J Org Chem* 2014; **79**(12):5471-7. doi: 10.1021/jo5005108.

29. Mroczek A. Studies on the constituents of *Amaranthus caudatus* Leaves. *Phytochem Rev* 2015; **14**:577-605.

30. Pedersen HA, Steffensen SK, Christophersen C *et al.* Synthesis and quantitation of six phenolic amides in *Amaranthus* spp. *J Agric Food Chem* 2010; **58**(10):6306-11. doi: 10.1021/jf100002v.

31. Sobolewska D, Podolak I, Makowska-Was J. *Allium ursinum*: botanical, phytochemical and pharmacological overview. *Phytochem Rev* 2015; **14**(1):81-97. doi: 10.1007/s11101-013-9334-0.

32. El Shabrawy MOA, Hosni HA, El Garf IA *et al.* Flavonoids from *Allium myrianthum* Boiss. *Biochem Syst Ecol* 2014; **56**:125-128. doi: 10.1016/j.bse.2014.05.015.

33. Sobolewska D, Michalska K, Podolak I *et al.* Steroidal saponins from the genus *Allium*. *Phytochem Rev* 2016; **15**:1-35. doi: 10.1007/s11101-014-9381-1.

34. Okoth DA, Chenia HY, Koorbanally NA. Antibacterial and antioxidant activities of flavonoids from *Lannea alata* (Engl.) Engl. (Anacardiaceae). Phytoche*mistry Letters* 2013; **6**(3):476-481. doi: 10.1016/j.phytol.2013.06.003.

35. Feuereisen MM, Hoppe J, Zimmermann BF *et al.* Characterization of phenolic compounds in Brazilian pepper (*Schinus terebinthifolius* Raddi) exocarp. *J Agric Food Chem* 2014; **62**(26):6219-26. doi: 10.1021/jf500977d.

36. Yun XJ, Shu HM, Chen GY *et al.* Chemical Constituents from Barks of *Lannea coromandelica*. *Chinese Herbal Medicines* 2014; **6**(1):65-69. doi: 10.1016/s1674-6384(14)60009-5.

37. I Umadevi MD, S D Sabnis. Chemotaxonomic studies on some members of Anacardiaceae. *Proc. Indian Acad. Sci. (Plant Sci.)* 1998; **98**(3):205-108.

38. Turini FG, Steinert C, Heubl G *et al.* Microsatellites facilitate species delimitation in Congolese *Ancistrocladus* (Ancistrocladaceae), a genus with pharmacologically potent naphthylisoquinoline alkaloids. *Taxon* 2014; **63**(2):329-341. doi: 10.12705/632.36.

39. Ibrahim SR, Mohamed GA. Naphthylisoquinoline alkaloids potential drug leads. *Fitoterapia* 2015; **106**:194-225. doi: 10.1016/j.fitote.2015.09.014.

40. Chun-Ping Tang Y-PY, Yi Zhong *et al.* Four new naphthylisoquinoline alkaloids from *Ancistrocladus tectorius*. *J Nat Prod* 2000; **63**:1384-1387.

41. de Oliveira Teles MN, Dutra LM, Barison A *et al.* Alkaloids from leaves of *Annona salzmannii* and *Annona vepretorum* (Annonaceae). *Biochem Syst Ecol* 2015; **61**:465-469. doi: 10.1016/j.bse.2015.07.016.

42. Aminimoghadamfarouj N, Nematollahi A, Wiart C. Annonaceae: bio-resource for tomorrow's drug discovery. *J Asian Nat Prod Res* 2011; **13**(5):465-76. doi: 10.1080/10286020.2011.570265.

43. de Fátima Costa Santos M, Dutra LM, Regina de Souza Moraes V *et al.* Aporphine alkaloids from the stem bark of *Guatteria pogonopus* (Annonaceae). *Biochem Syst Ecol* 2015; **60**:106-109. doi: 10.1016/j.bse.2015.04.011.

44. da Cruz PEO, Costa EV, Moraes VRdS *et al.* Chemical constituents from the bark of *Annona salzmannii* (Annonaceae). *Biochem Syst Ecol* 2011; **39**(4-6):872-875. doi: 10.1016/j.bse.2011.06.008.

45. Dutra LM, Costa EV, Moraes VRdS *et al.* Chemical constituents from the leaves of *Annona pickelii* (Annonaceae). *Biochem Syst Ecol* 2012; **41**:115-118. doi: 10.1016/j.bse.2011.12.011.

46. Vendramin ME, Costa EV, Pereira dos Santos É *et al.* Chemical constituents from the leaves of *Annona rugulosa* (Annonaceae). *Biochem Syst Ecol* 2013; **49**:152-155. doi: 10.1016/j.bse.2013.03.005.

47. Couvreur TL, Richardson JE, Sosef MS *et al.* Evolution of syncarpy and other morphological characters in African Annonaceae: a posterior mapping approach. *Mol Phylogenet Evol* 2008; **47**(1):302-18. doi: 10.1016/j.ympev.2008.01.018.

48. Campos FR, Batista RL, Batista CL *et al.* Isoquinoline alkaloids from leaves of *Annona sericea* (Annonaceae). *Biochem Syst Ecol* 2008; **36**(10):804-806. doi: 10.1016/j.bse.2008.07.005.

49. Costa EV, Dutra LM, Nepel A *et al.* Isoquinoline alkaloids from the leaves of *Xylopia laevigata* (Annonaceae). *Biochem Syst Ecol* 2013; **51**:331-334. doi: 10.1016/j.bse.2013.10.005.

50. Nik Abdullah Zawawi NK, Ahmat N, Ahmad R *et al.* Oxoaporphine alkaloids and flavonols from *Xylopia ferruginea* (Annonaceae). *Biochem Syst Ecol* 2012; **43**:7-9. doi: 10.1016/j.bse.2012.02.019.

51. Galle J-B, Leti M, Kim S *et al.* Unusual benzylated flavonoids from *Mitrella mesnyi* (Pierre) Bân. *Biochem Syst Ecol* 2013; **48**:9-11. doi: 10.1016/j.bse.2012.11.023.

52. Shi Mr, Pe D, Liu Jx *et al.* Chemical constituents from *Sphallerocarpus gracilis*. *Biochem Syst Ecol* 2012; **40**:1-3. doi: 10.1016/j.bse.2011.09.008.

53. Qin N, Su Y-F, Wang Y-D *et al.* Chemical constituents from *Tongoloa silaifolia*. *Biochem Syst Ecol* 2012; **44**:380-382. doi: 10.1016/j.bse.2012.06.022.

54. Iranshahy M, Iranshahi M. Traditional uses, phytochemistry and pharmacology of asafoetida (Ferula assa-foetida oleo-gum-resin)-a review. *J Ethnopharmacol* 2011; **134**(1):1-10. doi: 10.1016/j.jep.2010.11.067.

55. Su Z. Phytochemistry of Ranunculaceae and Apocynacea. *Journal of International Pharmaceutical Research* 1978; **3**:129-133.

56. JP. Zhu AG, M. Kalt-Hadamowsky, M. Hesse. Chemotaxonomic study of the genus *Tabernaemontana*. *Plant Systematics and Evolution* 1990; **172**:13-34.

57. Şöhretoğlu D, Masullo M, Piacente S *et al.* Iridoids, monoterpenoid glucoindole alkaloids and flavonoids from *Vinca major*. *Biochem Syst Ecol* 2013; **49**:69-72. doi: 10.1016/j.bse.2013.03.028.

58. Wang D-Q, Wu C-N, Song Y *et al.* Chemical constituents from *Ilex urceolatus*. *Biochem Syst Ecol* 2016; **64**:70-73. doi: 10.1016/j.bse.2015.11.018.

59. Wu Y, Zhang X-C, Sun J-B *et al.* Chemical constituents from the leaves of *Ilex urceolatus*. *Biochem Syst Ecol* 2014; **54**:92-95. doi: 10.1016/j.bse.2014.01.002.

60. Hao D, Gu X, Xiao P *et al.* Research progress in the phytochemistry and biology of *Ilex* pharmaceutical resources. *Acta Pharmaceutica Sinica B* 2013; **3**(1):8-19. doi: 10.1016/j.apsb.2012.12.008.

61. Franke K, Hoffmann M, Schmidt J *et al.* 200-O-Glucosylvitexin, a chemotaxonomic marker for the genus *Cryptocoryne* (Araceae). *Biochem Syst Ecol* 2006; **34**(6):546-548. doi: 10.1016/j.bse.2005.10.017.

62. Wu Y-Y, Huang X-X, Zhang M-Y *et al.* Chemical constituents from the tubers of *Pinellia ternata* (Araceae) and their chemotaxonomic interest. *Biochem Syst Ecol* 2015; **62**:236-240. doi: 10.1016/j.bse.2015.09.002.

63. Hong Van NT, Van Minh C, De Leo M *et al.* Secondary metabolites from *Lasia spinosa* (L.) Thw. (Araceae). *Biochem Syst Ecol* 2006; **34**(12):882-884. doi: 10.1016/j.bse.2006.04.011.

64. Wang D, Koh HL, Hong Y *et al.* Chemical and morphological variations of *Panax notoginseng* and their relationship. *Phytochemistry* 2013; **93**:88-95. doi: 10.1016/j.phytochem.2013.03.007.

65. Hu H-B, Fan J. Chemical constituents from *Acanthopanax brachypus*. *Biochem Syst Ecol* 2012; **43**:67-72. doi: 10.1016/j.bse.2012.02.004.

66. Cao K-y, Qiao C-f, Chen X-q *et al.* Chemical constituents from leaves of *Oplopanax horridus*. *Chinese Herbal Medicines* 2014; **6**(4):328-331. doi: 10.1016/s1674-6384(14)60050-2.

67. Zhu GY, Li YW, Hau DK *et al.* Protopanaxatriol-type ginsenosides from the root of *Panax ginseng*. *J Agric Food Chem* 2011; **59**(1):200-5. doi: 10.1021/jf1037932.

68. Huang HC, Liaw CC, Zhang LJ *et al.* Triterpenoidal saponins from *Hydrocotyle sibthorpioides*. *Phytochemistry* 2008; **69**(7):1597-603. doi: 10.1016/j.phytochem.2008.01.005.

69. DJ Ofman, KR Markham, C Vilain *et al.* Flavonoid profiles of New Zealand kauri and other species of *Agathis*. *Phytochemistry Letters* 1995; **38**(5):1223-1228.

70. Cox RE, Yamamoto S, Otto A *et al.* Oxygenated di- and tricyclic diterpenoids of southern hemisphere conifers. *Biochem Syst Ecol* 2007; **35**(6):342-362. doi: 10.1016/j.bse.2006.09.013.

71. Heinrich M, Dhanji T, Casselman I. Açai (*Euterpe oleracea* Mart.)—A phytochemical and pharmacological assessment of the species’ health claims. *Phytochemistry Letters* 2011; **4**(1):10-21. doi: 10.1016/j.phytol.2010.11.005.

72. Abadio Finco FD, Kammerer DR, Carle R *et al.* Antioxidant activity and characterization of phenolic compounds from bacaba (*Oenocarpus bacaba* Mart.) fruit by HPLC-DAD-MS(n). *J Agric Food Chem* 2012; **60**(31):7665-73. doi: 10.1021/jf3007689.

73. Koolen HHF, da Silva FMA, Gozzo FC *et al.* Antioxidant, antimicrobial activities and characterization of phenolic compounds from buriti (*Mauritia flexuosa* L. f.) by UPLC–ESI-MS/MS. *Food Research International* 2013; **51**(2):467-473. doi: 10.1016/j.foodres.2013.01.039.

74. Kang J, Xie C, Li Z *et al.* Flavonoids from acai (*Euterpe oleracea* Mart.) pulp and their antioxidant and anti-inflammatory activities. *Food Chem* 2011; **128**(1):152-7. doi: 10.1016/j.foodchem.2011.03.011.

75. de Oliveira DM, Siqueira EP, Nunes YRF *et al.* Flavonoids from leaves of *Mauritia flexuosa*. *Revista Brasileira de Farmacognosia* 2013; **23**(4):614-620. doi: 10.1590/s0102-695x2013005000061.

76. Del Nero Rodrigues C, Salatino A. Hydrocarbons from epicuticular waxes of *Allagoptera* (Arecaceae). *Biochem Syst Ecol* 2006; **34**(3):265-266. doi: 10.1016/j.bse.2005.10.001.

77. El-Dib R, Kaloga M, Mahmoud I *et al.* Sablacaurin A and B, two 19-nor-3,4-seco-lanostane-type triterpenoids from *Sabal causiarum* and *Sabal blackburniana*, respectively. *Phytochemistry* 2004; **65**(8):1153-7. doi: 10.1016/j.phytochem.2004.02.026.

78. Rencoret J, Ralph J, Marques G *et al.* Structural characterization of lignin isolated from coconut (*Cocos nucifera*) coir fibers. *J Agric Food Chem* 2013; **61**(10):2434-45. doi: 10.1021/jf304686x.

79. Simas FF, Gorin PA, Guerrini M *et al.* Structure of a heteroxylan of gum exudate of the palm *Scheelea phalerata* (uricuri). *Phytochemistry* 2004; **65**(16):2347-55. doi: 10.1016/j.phytochem.2004.06.004.

80. Simas FF, Maurer-Menestrina J, Reis RA *et al.* Structure of the fucose-containing acidic heteroxylan from the gum exudate of *Syagrus romanzoffiana* (Queen palm). *Carbohydrate Polymers* 2006; **63**(1):30-39. doi: 10.1016/j.carbpol.2005.06.015.

81. Kårehed J. Argophyllaceae. *The Families and Genera of Vascular Plants* 2007; **8**:13-18.

82. Iwashina T, Kitajima J, Shiuchi T *et al.* Chalcones and other flavonoids from *Asarum* sensu lato (Aristolochiaceae). *Biochem Syst Ecol* 2005; **33**(6):571-584. doi: 10.1016/j.bse.2004.12.005.

83. Silva-Brandão KL, Solferini VN, Trigo JR. Chemical and phylogenetic relationships among *Aristolochia* L. (Aristolochiaceae) from southeastern Brazil. *Biochem Syst Ecol* 2006; **34**(4):291-302. doi: 10.1016/j.bse.2005.10.011.

84. Francisco CS, Messiano GB, Lopes LM *et al.* Classification of *Aristolochia* species based on GC-MS and chemometric analyses of essential oils. *Phytochemistry* 2008; **69**(1):168-75. doi: 10.1016/j.phytochem.2007.07.007.

85. Dai Y, Harinantenaina L, Brodie PJ *et al.* Antiproliferative homoisoflavonoids and bufatrienolides from *Urginea depressa*. *J Nat Prod* 2013; **76**(5):865-72. doi: 10.1021/np300900a.

86. Kumeta Y, Maruyama T, Wakana D *et al.* Chemical analysis reveals the botanical origin of shatavari products and confirms the absence of alkaloid asparagamine A in *Asparagus racemosus*. *J Nat Med* 2013; **67**(1):168-73. doi: 10.1007/s11418-012-0669-4.

87. Alali F, El-Elimat T, Albataineh H *et al.* Cytotoxic homoisoflavones from the bulbs of *Bellevalia eigii*. *J Nat Prod* 2015; **78**(7):1708-15. doi: 10.1021/acs.jnatprod.5b00357.

88. Chou C-H, Hsu Y-M, Huang T-J *et al.* Sterodial sapogenins from *Solanum torvum*. *Biochem Syst Ecol* 2012; **45**:108-110. doi: 10.1016/j.bse.2012.07.021.

89. Triana J, Eiroa JL, Ortega JJ *et al.* Chemotaxonomy of *Gonospermum* and related genera. *Phytochemistry* 2010; **71**(5-6):627-34. doi: 10.1016/j.phytochem.2009.12.013.

90. Herout V. Chemotaxonomy of the family Compositae (Asteraceae). *Pharmacognosy and Phytochemistry* 1971:93-110.

91. Yu xinyuan Yx, Zhou Xiaoping. Progress research on chemical composition and pharmacological action of Compositae. *Journal of Jilin University (Medicine Edition)* 2005; **31**(1):159-162.

92. Fan J-Y, Chen H-B, Zhu L *et al.* Saussurea medusa, source of the medicinal herb snow lotus: a review of its botany, phytochemistry, pharmacology and toxicology. *Phytochemistry Reviews* 2015; **14**(3):353-366. doi: 10.1007/s11101-015-9408-2.

93. Zhi-Hong Jiang, Xiao-Yun Wen, Takashi Tanaka *et al.* Cytotoxic hydrolyzable tannins from *Balanophora japonica*. *J. Nat. Prod.* 2008; **71**:719-723.

94. Zhi-Hong, Jiang TT, Hiromi Iwata, *et al.* Ellagitannins and lignan glycosides from *Balanophora japonica* (Balanophoraceae). *Chem. Pharm. Bull.* 2005; **53**(3):339-341.

95. Abiodun Ogundaini MF, Premila Perera, Gunnar Samuelsson *et al.* Isolation of two new antiinflammatory biflavanoids from *Sarcophyte piriei*. *J. Nat. Prod.* 1996; **59**:587-590.

96. B. Yadagiri KR, G.S.R. Subba Rao. Triterpenoids from *Balanophora abbreviata* and *Balanophora indica*. *J Nat Prod* 1984; **47**(1):182-191.

97. Luo B. Study on the chemical constitute of *Balanophora involucrate*. *Lishizhen Med Res* 2007; **18**(8):1929-1930.

98. Johan Svenson BMS, Nigel I. Joyce, Catherine E. Sansom *et al.* Betalains in red and yellow varieties of the andean tuber crop ulluco (*Ullucus tuberosus*). *J. Agric. Food Chem.* 2008; **56**(7730-7737).

99. Alfonso Espada RR. Boussingoside E, a new triterpenoid saponin from the tubers of *Boussingaultia baselloides*. *J. Nat. Prod.* 1997; **60**: 17-19.

100. Huey-Yi Lin S-CK, Pei-Dawn Lee Chao, Tzong-Dann Lin. A new sapogenin from *Boussingaultia gracilis*. *Joumal of Natural Prodcts* 1988; **51**(4):797-798.

101. lwashin T. The structure and distribution of the flavonoids in plants. *J. Plant Res.* 2000; **113**:287-299.

102. Riitta Julkunen-Tiitto MR, John Bryant, Sinikka Sorsa *et al.* Chemical diversity of several Betulaceae species: comparison of phenolics and terpenoids in northern birch stems. *Trees* 1996; **11**: 16-22.

103. A. Lavola RJ-T. The effect of elevated carbon dioxide and fertilization on primary and secondary metabolites in birch, betula. *Oecologia* 1994; **99**:315-321.

104. Millet A, Stintzing F, Merfort I. Flavonol quantification and stability of phenolics in fermented extracts from fresh *Betula pendula* leaves. *J Pharm Biomed Anal* 2010; **53**(2):137-44. doi: 10.1016/j.jpba.2010.02.001.

105. Lahtinen M, Lempa K, Salminen J-P *et al.* HPLC analysis of leaf surface flavonoids for the preliminary classification of birch species. *Phytochemical Analysis* 2006; **17**(3):197-203. doi: 10.1002/pca.906.

106. Markku Keinanen RJ-T, Matti Rousi, Tahvanainen J. Taxonomic implications of phenolic variation in leaves of birch (*Betula* L.) species. *Biochem Syst Ecol* 1999; **27**:243-254.

107. Muellner AN. Biebersteiniaceae. *The Families and Genera of Vascular Plants* 2010; **10**:72-75.

108. Olga Tzakoua AY, Dionyssios Vassiliades. Investigation of the C16_3_C18_3 fatty acid balance in leaf tissues of *Biebersteinia orphanidis* Boiss. (Biebersteiniaceae). *Biochem Syst Ecol* 2001; **29**:765-767.

109. Stefan Gafner J-LW, Malo Nianga, Helen Stoeckli-Evans *et al.* Antifungal and antibacterial naphthoquinones from *Newbouldia laevis* roots. *Phytochemistry* 1996; **42**(5):1315-1320.

110. Frederic Martin A-EH, Valentin R. Quinteros Condoretty, Delphine Cressend *et al.* Antioxidant phenylethanoid glycosides and a neolignan from *Jacaranda caucana*. *J. Nat. Prod.* 2009; **72**:852-856.

111. Gilsane Lino von Poser JS, Amelia T. Henriques, Soren Rosendal Jensen. The distribution of iridoids in Bignoniaceae. *Biochem Syst Ecol* 2000; **28**:351-366.

112. Blatt C, Santos M, Salatino A. Flavonoids of Bignoniaceae from the"cerrado"and their possible taxonomic significance. *Plant Syst Evol* 1998; **210**:289-292.

113. S. Satyavathi MR, Narayan LL. Numerical chemotaxonomy of some Bignoniaceae *Feddes Repertorium* 1987; **98**(7-8):391-397.

114. Okusa PN, Beuerle T, Stévigny C *et al.* Absence of pyrrolizidine alkaloids in *Cordia gilletii* de wild (Boraginaceae). *Biochem Syst Ecol* 2012; **41**:1-2. doi: 10.1016/j.bse.2011.12.002.

115. Colegate SM, Welsh SL, Gardner DR *et al.* Profiling of dehydropyrrolizidine alkaloids and their N-oxides in herbarium-preserved specimens of *Amsinckia* species using HPLC-esi(+)MS. *J Agric Food Chem* 2014; **62**(30):7382-92. doi: 10.1021/jf500425v.

116. Damianakos H, Sotiroudis G, Chinou I. Pyrrolizidine alkaloids from *Onosma erecta*. *J Nat Prod* 2013; **76**(10):1829-35. doi: 10.1021/np300785g.

117. Cairns E, Hashmi MA, Singh AJ *et al.* Structure of echivulgarine, a pyrrolizidine alkaloid isolated from the pollen of *Echium vulgare*. *J Agric Food Chem* 2015; **63**(33):7421-7. doi: 10.1021/acs.jafc.5b02402.

118. Huang Z. Chemical and pharmacological activity of Boraginaceae. *Natural product and development* 2000; **12**(1):73-81.

119. Baenas N, Garcia-Viguera C, Moreno DA. Biotic elicitors effectively increase the glucosinolates content in Brassicaceae sprouts. *J Agric Food Chem* 2014; **62**(8):1881-9. doi: 10.1021/jf404876z.

120. Brock A, Herzfeld T, Paschke R *et al.* Brassicaceae contain nortropane alkaloids. *Phytochemistry* 2006; **67**(18):2050-7. doi: 10.1016/j.phytochem.2006.06.024.

121. Marzouk MM, Al-Nowaihi A-SM, Kawashty SA *et al.* Chemosystematic studies on certain species of the family Brassicaceae (Cruciferae) in Egypt. *Biochem Syst Ecol* 2010; **38**(4):680-685. doi: 10.1016/j.bse.2010.04.004.

122. Radulović N, Zlatković B, Skropeta D *et al.* Chemotaxonomy of the peppergrass *Lepidium coronopus* (L.) Al-Shehbaz (syn. *Coronopus squamatus*) based on its volatile glucosinolate autolysis products. *Biochem Syst Ecol* 2008; **36**(10):807-811. doi: 10.1016/j.bse.2008.07.006.

123. Felice Senatore MDA, Irene Dini. Flavonoid Glycosides of *Barbarea vulgaris* L. (Brassicaceae). *J. Agric. Food Chem.* 2000; **48**:2659-2662.

124. Agerbirk N, Ørgaard M, Nielsen JK. Glucosinolates, flea beetle resistance, and leaf pubescence as taxonomic characters in the genus *Barbarea* (Brassicaceae). *Phytochemistry* 2003; **63**(1):69-80. doi: 10.1016/s0031-9422(02)00750-1.

125. Robert F. Raffauf MDM, Philip W. Le Quesne. Antitumor plants. 11. Diterpenoid and flavonoid constituents of *Bromelia pinguin* L. *J.Org. Chem.* 1981; **46**:1094-1098.

126. Maricela Adrian-Romero GB. Betaine distribution in the Bromeliaceae. *Biochem Syst Ecol* 2001; **29**:305-311.

127. S. Ankanna DS, N. Savithramma. Chemotaxonomical studies of some important Monocotyledons. *Botany Research International* 2012; **5**(4):90-96. doi: 10.5829/idosi.bri.2012.5.4.501.

128. Raimundo GdO-J, Ana PdO, Amanda LG *et al.* The first flavonoid isolated from *Bromelia laciniosa* (Bromeliaceae). *J Med Plants Res* 2014; **8**(14):558-563. doi: 10.5897/jmpr2014.5375.

129. Raimundo Gonçalves de Oliveira-Junior GRS, Amanda Leite Guimarães, Ana Paula de Oliveira *et al.* Photoprotective, antibacterial activity and determination of phenolic compounds of *Neoglaziovia variegata* (Bromeliaceae) by high performance liquid chromatography-diode array detector (HPLC-DAD) analysis. *African Journal of Pharmacy and Pharmacology* 2015; **9**(22):556-584. doi: 10.5897/ajpp2015.

130. Rajagopal MS. *Canarium patentinervium* Miq. (Burseraceae kunth.): a phytochemical and pharmacological study. *Thesis (University of Nottingham only) (PhD)* 2014.

131. Jane B.G. Siqueira MDGBZ, Jose A. Cabral, and Wilson W. Filho; Lignans from *Protium tenuifolium*. *J Nat Prod* 1995; **58**(5):730-732.

132. Al-Harrasi A, Ali L, Rehman NU *et al.* Nine triterpenes from *Boswellia sacra* Flückiger and their chemotaxonomic importance. *Biochem Syst Ecol* 2013; **51**:113-116. doi: 10.1016/j.bse.2013.08.026.

133. Atta-UR-Rahman DA, M. Iqbal Choudhar. Alkaloids from the leaves of *Buxus sempervirens*. *J Nat Prod* 1998; **51**(4):783-786.

134. Atta-ur-Rahman MIC, M. Riaz Khan *et al.* New steroidal alkaloids from *Sarcococca saligna*. *J Nat Prod* 2000; **63**: 1364-1368.

135. M. Iqbal Choudhary SS, Shehnaz Parveen *et al.* New triterpenoid alkaloid cholinesterase inhibitors from *Buxus hyrcana*. *J Nat Prod* 2003; **66**:739-742.

136. Qiu minghua Ld; Study on chemotoxanomy of Buxaceae. *Chinese Journal of Applied and Environmental Biology* 2002; **8**(4):387-391.

137. Flores Ortiz CM, Dávila P, Portilla LBH. Alkaloids from *Neobuxbaumia* species (Cactaceae). *Biochem Syst Ecol* 2003; **31**(6):581-585. doi: 10.1016/s0305-1978(02)00220-x.

138. Starha R. Alkaloids from the cactus genus *Gymnocalycium* (Cactaceae). *BiochemicalSystematicsandEcology* 1996; **24**(1):85-86.

139. Romans Starha KU, Jaroslav Kuchyna. Alkaloids from the genus *Gymnocalycium* (Cactaceae)-II. *BiochemicalSysternaticsandEcology* 1997; **25**(4):363-364.

140. Ewell EE. The Chemistry of the Cactaceae. *J. Am. Chem. Soc.* 1896; **18**(7):624-643.

141. Okazaki S, Kinoshita K, Ito S *et al.* Triterpenoid saponins from *Echinopsis macrogona* (Cactaceae). *Phytochemistry* 2011; **72**(1):136-46. doi: 10.1016/j.phytochem.2010.10.004.

142. Shu-Geng Ccao K-YS, Joan Pereira, Swee-Hockgoh. Coumarins from *Calophyllum teysmannii* (Calophyllaceae). *Phytochemisto* 1998; **47**(6):1051-1055.

143. Munekazu Inuma HT, Naeko Toriyama, Toshiyuki Tanaka *et al.* Six xanthones from *Calophyllum austroindicum* (Calophyllaceae). *Phytochemistry* 1996; **43**(3):681-685.

144. Anake Kijjoa MJG, Madalena M.M. Pinto *et al.* Xanthones from *Calophyllum teysmannii* var. *inophylloide* (Calophyllaceae). *Phytochemistry* 2000; **55**:833-836.

145. Hellwig FH. Calyceraceae. *The Families and Genera of Vascular Plants* 2007; **8**:19-25.

146. Bruce A. Bohm AR, Melanie DeVore, Tod F. Stuessy. Flavonoid chemistry of Calyceraceae. *Canadian Journal of Botany* 1995; **73**(12):1962-1965.

147. Wang Z-T, Ma G-Y, Tu P-F *et al.* Chemotaxonomic study of *Codonopsis* (family Campanulaceae) and its related genera. *Biochem Syst Ecol* 1996; **23**(7):809-812.

148. He JY, Ma N, Zhu S *et al.* The genus *Codonopsis* (Campanulaceae): a review of phytochemistry, bioactivity and quality control. *Journal of Natural Medicines* 2015; **69**(1):1-21. doi: 10.1007/s11418-014-0861-9.

149. Julie R. Kesting I-LT, Anders F. Pedersen *et al.* Piperidine and tetrahydropyridine alkaloids from *Lobelia siphilitica* and *Hippobroma longiflora*. *J Nat Prod* 2009; **72**(2):312-315.

150. Karl W. Hillig PGM. A chemotaxonomic analysis of cannabinoid variation in *Cannabis* (Cannabaceae). *Am J Bot*2004; **91**(6):966-975.

151. Hillig KW. A chemotaxonomic analysis of terpenoid variation in *Cannabis*. *Biochem Syst Ecol* 2004; **32**(10):875-891. doi: 10.1016/j.bse.2004.04.004.

152. Fischedick JT, Hazekamp A, Erkelens T *et al.* Metabolic fingerprinting of *Cannabis sativa* L., cannabinoids and terpenoids for chemotaxonomic and drug standardization purposes. *Phytochemistry* 2010; **71**(17-18):2058-73. doi: 10.1016/j.phytochem.2010.10.001.

153. Radwan MM, Elsohly MA, Slade D *et al.* Non-cannabinoid constituents from a high potency *Cannabis sativa* variety. *Phytochemistry* 2008; **69**(14):2627-33. doi: 10.1016/j.phytochem.2008.07.010.

154. Kers LE. Capparaceae. *The Families and Genera of Vascular Plants* 2003; **5**:36-56.

155. Pelotto JP, Del MaA, Martınez P; Flavonoid aglycones from Argentinian *Capparis* Species (Capparaceae). *Biochem Syst Ecol* 1998; **26**:577-580.

156. Romeo V, Ziino M, Giuffrida D *et al.* Flavour profile of capers (*Capparis spinosa* L.) from the Eolian Archipelago by HS-SPME/GC–MS. *Food Chemistry* 2007; **101**(3):1272-1278. doi: 10.1016/j.foodchem.2005.12.029.

157. Matthaus B, Ozcan M. Glucosinolates and fatty acid, sterol, and tocopherol composition of seed oils from *Capparis*. *J. Agric. Food Chem.* 2005; **53**(7136-7141).

158. William F.H. McLean, Gerald Blunden, Kenneth Jewers. Quaternary ammonium compounds in the Capparaceae. *Biochem Syst Ecol* 1996; **24**(5):427-434.

159. Jordheim M, Giske NH, Andersen ØM. Anthocyanins in Caprifoliaceae. *Biochem Syst Ecol* 2007; **35**(3):153-159. doi: 10.1016/j.bse.2006.09.010.

160. Rezgui A, Mitaine-Offer AC, Miyamoto T *et al.* Oleanolic acid and hederagenin glycosides from *Weigela stelzneri*. *Phytochemistry* 2016; **123**:40-7. doi: 10.1016/j.phytochem.2015.12.016.

161. Guido Flamini AB, Pier Luigi Cioni, Ivano Morelli. Three new flavonoids and other constituents from *Lonicera implexa*. *J. Nat. Prod.* 1997; **60**:449-452.

162. Zeng L. Preliminary study on chemotaxonomy of Caprifoliaceae. *China J Chi Materia Med* 2000; **25**(3):184-187.

163. Bennett R, Kiddl G, Wallsgrove RM. Biosynthesis of benzylglucosinolate, cyanogenic glucosides and phenylpropanoids in *Carica papaya*. *Phytochemistry* 1997; **45**(1):58-66.

164. Canini A, Alesiani D, D’Arcangelo G *et al.* Gas chromatography–mass spectrometry analysis of phenolic compounds from *Carica papaya* L. leaf. *Journal of Food Composition and Analysis* 2007; **20**(7):584-590. doi: 10.1016/j.jfca.2007.03.009.

165. Gayosso-García Sancho LE, Yahia EM, González-Aguilar GA. Identification and quantification of phenols, carotenoids, and vitamin C from papaya (*Carica papaya* L., cv. Maradol) fruit determined by HPLC-DAD-MS/MS-ESI. *Food Research International* 2011; **44**(5):1284-1291. doi: 10.1016/j.foodres.2010.12.001.

166. F. Marx EHAA, J.G. Maia. Chemical composition of the fruit pulp of *Caryocar villosum*. *Z Lebensm Unters Forsch A* 1997; **204**:442-444.

167. Chiste RC, Mercadante AZ. Identification and quantification, by HPLC-DAD-MS/MS, of carotenoids and phenolic compounds from the Amazonian fruit *Caryocar villosum*. *J Agric Food Chem* 2012; **60**(23):5884-92. doi: 10.1021/jf301904f.

168. Abdulmagid Alabdul Magid LV, Christian Moretti, Christophe Long *et al.* Triterpenoid saponins from the fruits of *Caryocar glabrum*. *J. Nat. Prod.* 2006; **69**:196-205.

169. Luisa Pistelli RV, Antonio Marsili, Ivano Morelli. Alkaloids and coumarins from *Gymnosporia senegalensis* var. *spinosa* (Celastraceae). *Biochem Syst Ecol* 1998; **26**:677-679.

170. Alarcon J, Cespedes CL, Munoz E *et al.* Dihydroagarofuranoid sesquiterpenes as acetylcholinesterase inhibitors from Celastraceae plants: Maytenus disticha and Euonymus japonicus. *J Agric Food Chem* 2015; **63**(47):10250-6. doi: 10.1021/acs.jafc.5b04168.

171. Torres-Romero D, King-Diaz B, Strasser RJ *et al.* Friedelane triterpenes from *Celastrus vulcanicola* as photosynthetic inhibitors. *J Agric Food Chem* 2010; **58**(20):10847-54. doi: 10.1021/jf1022115.

172. Wagner Vilegas MS, Luca Rastrelli, and Cosimo Pizza. Isolation and structure elucidation of two new flavonoid glycosides from the infusion of *Maytenus aquifolium* Leaves. Evaluation of the Antiulcer Activity of the Infusion. *J. Agric. Food Chem.* 1999; **47**:403-406.

173. Ye G, Peng H, Fan M *et al.* A new hopane triterpene from *Dipentodon sinicus* (Celastraceae). *Biochem Syst Ecol* 2007; **35**(12):905-908. doi: 10.1016/j.bse.2007.05.004.

174. Conran JG. Cephalotaceae. *The Families and Genera of Vascular Plants* 2004; **6**:65-68.

175. Satyajit D. Sarker PW, Rene Lafont, Jean-Pierre Girault *et al.* Cucurbitacin D from *Cercidiphyllum japonicum*. *Biochem Syst Ecol* 1997; **25**(1):29-80.

176. Giannasi DE. Phytochemical Aspects of Phylogeny in Hamamelidae. *Annals of the Missouri Botanical Garden* 1986; **73**(2):417-437.

177. Liu C, Li G, Huang R *et al.* Chemotaxonomic significance of sesquiterpenes and amide derivatives from *Chloranthus angustifolius* Oliv. *Biochem Syst Ecol* 2015; **58**:30-33. doi: 10.1016/j.bse.2014.10.011.

178. Wang F, Zhou DS, Wei GZ *et al.* Chlorantholides A-F, eudesmane-type sesquiterpene lactones from *Chloranthus elatior*. *Phytochemistry* 2012; **77**:312-7. doi: 10.1016/j.phytochem.2012.02.008.

179. Lorenzo D, Loayza I, Dellacassa E. Composition of the essential oils from leaves of two *Hedyosmum* spp. from Bolivia. *Flavour and Fragrance Journal* 2003; **18**(1):32-35. doi: 10.1002/ffj.1146.

180. Tolardo R, Zetterman L, Bitencourtt DR *et al.* Evaluation of behavioral and pharmacological effects of *Hedyosmum brasiliense* and isolated sesquiterpene lactones in rodents. *J Ethnopharmacol* 2010; **128**(1):63-70. doi: 10.1016/j.jep.2009.12.026.

181. Haifeng Wu XH, Xiaopo Zhang, Shilin Chen *et al.* Isolation and chemotaxonomic significance of megastigmane-type sesquiterpenoids from *Sarcandra glabra*. *J Med Plants Res* 2012; **6**(28):4501-4504. doi: 10.5897/jmpr12.523.

182. Zhang M, Wang J, Luo J *et al.* Labdane diterpenes from *Chloranthus serratus*. *Fitoterapia* 2013; **91**:95-9. doi: 10.1016/j.fitote.2013.08.015.

183. Amoah SK, de Oliveira FL, da Cruz AC *et al.* Sesquiterpene lactones from the leaves of *Hedyosmum brasiliense* (Chloranthaceae). *Phytochemistry* 2013; **87**:126-32. doi: 10.1016/j.phytochem.2012.11.018.

184. Xiong J, Liu S-T, Tang Y *et al.* Sesquiterpenoids from the aerial parts of *Chloranthus elatior*. *Phytochemistry Letters* 2013; **6**(4):586-589. doi: 10.1016/j.phytol.2013.07.015.

185. Jun Kawabata YF, Satoshi Tahara, Junya Mizutani. Structures of novel sesquiterpene ketones from *Chloranthus serratus* (Chloranthaceae). *Agricultural and Biological Chemistry* 1985; **49**(5):1479-1485. doi: 10.1080/00021369.1985.10866891.

186. Lidio Coradin DEG, Ghillean T. Prance. Chemosystematic studies in the Chrysobalanaceae. I. Flavonoids in *Parinari*. *Brittonia* 1985; **37**(2):169-178.

187. Robles C, Bousquet-Mélou A, Garzino S *et al.* Comparison of essential oil composition of two varieties of *Cistus ladanifer*. *Biochem Syst Ecol* 2003; **31**(3):339-343. doi: 10.1016/s0305-1978(02)00161-8.

188. Jesus M. L. Roldilla Dimdm, J. G. Uroness, R. F. Moros. Hydroxylated diterpenoids from *Halimium viscosum*. *Phyrochemrstry,* 1998; **49**(3):817-822.

189. Perihan Gürbüz LÖD, Zühal Güvenalp, Ayşe Kuruüzüm-Uz CK. Isolation and structure elucidation of secondary metabolites from *Cistus salviifolius* L. *Rec. Nat. Prod.* 2015; **9**(2):175-183.

190. Venditti A, Bianco A, Bruno M *et al.* Phytochemical study of *Cistus libanotis* L. *Nat Prod Res* 2015; **29**(2):189-92. doi: 10.1080/14786419.2014.968569.

191. Claude Spino J, Subramaniam Sotheeswaran, William Albersber. Three prenylated phenolic benzophenones from *Garcinia myrtifolia*. *Phytochemistry* 1996; **38**(1):233-236.

192. Nguyen L-HD, Vo HT, Pham HD *et al.* Xanthones from the bark of *Garcinia merguensis*. *Phytochemistry* 2003; **63**(4):467-470. doi: 10.1016/s0031-9422(02)00433-8.

193. Zhong FF, Chen Y, Mei ZN *et al.* Xanthones from the bark of *Garcinia Xanthochymus*. *Chinese Chemical Letters* 2007; **18**(7):849-851. doi: 10.1016/j.cclet.2007.05.045.

194. Rukachaisirikul V, Ritthiwigrom T, Pinsa A *et al.* Xanthones from the stem bark of *Garcinia nigrolineata*. *Phytochemistry* 2003; **64**(6):1149-1156. doi: 10.1016/s0031-9422(03)00502-8.

195. Nguyen HD, Trinh BTD, Nguyen NK *et al.* Xanthones from the twigs of *Cratoxylum cochinchinense*. *Phytochemistry Letters* 2011; **4**(1):48-51. doi: 10.1016/j.phytol.2010.11.006.

196. Adams M, Chammartin M, Hamburger M *et al.* Case study of the Swiss flora for prior phytochemical and biological investigations. *J Nat Prod* 2013; **76**(2):209-15. doi: 10.1021/np300682p.

197. Maroyi A. *Gloriosa superba* L. (family Colchicaceae): Remedy or poison? *J Med Plants Res* 2011; **5**(26). doi: 10.5897/jmpr11.913.

198. Feras Q. Alali TE-E, Chen Li *et al.* New Colchicinoids from a native jordanian meadow saffron, *Colchicum brachyphyllum*: isolation of the first naturally occurring dextrorotatory colchicinoid. *J. Nat. Prod.* 2005; **68**:173-178.

199. Sonny Larsson NR. Reviewing Colchicaceae alkaloids – perspectives of evolution on medicinal chemistry. *Current Topics in Medicinal Chemistry* 2014; **24**:274-289.

200. Cock IE. The medicinal properties and phytochemistry of plants of the genus *Terminalia* (Combretaceae). *Inflammopharmacology* 2015; **23**(5):203-29. doi: 10.1007/s10787-015-0246-z.

201. Crouzet S, Maria A, Dinan L *et al.* Ecdysteroids from *Cyanotis longifolia* Benth. (Commelinaceae). *Arch Insect Biochem Physiol* 2009; **72**(4):194-209. doi: 10.1002/arch.20329.

202. Calderón AI, Chung KS, Gupta MP. Ecdysteroids from *Dichorisandra hexandra* (Commelinaceae). *Biochem Syst Ecol* 2009; **37**(5):693-695. doi: 10.1016/j.bse.2009.10.008.

203. Schimming T, Jenett-Siems K, Mann P *et al.* Calystegines as chemotaxonomic markers in the Convolvulaceae. *Phytochemistry* 2005; **66**(4):469-80. doi: 10.1016/j.phytochem.2004.12.024.

204. Jenett-Siems K, Weigl R, Bohm A *et al.* Chemotaxonomy of the pantropical genus *Merremia* (Convolvulaceae) based on the distribution of tropane alkaloids. *Phytochemistry* 2005; **66**(12):1448-64. doi: 10.1016/j.phytochem.2005.04.027.

205. Acharjya SK, Bhattamisra SK, Muddana BR *et al.* Development of a high-performance liquid chromatographic method for determination of letrozole in wistar rat serum and its application in pharmacokinetic studies. *Sci Pharm* 2012; **80**(4):941-53. doi: 10.3797/scipharm.1206-06.

206. Petra Mann BT, Macki Kaloga, Eckart Eich. Flavonoid sulfates from the Convolvulaceae. *Phytochemistry* 1999; **50**:267-271.

207. Kim KH, Woo KW, Moon E *et al.* Identification of antitumor lignans from the seeds of morning glory (*Pharbitis nil*). *J Agric Food Chem* 2014; **62**(31):7746-52. doi: 10.1021/jf501470k.

208. M. Leela SSR. Phenolic compounds in the taxonomy of *Ipomoea* L. (Convolvulaceae). *Feddes Repertorium* 1994; **105**(7-8):445-448.

209. Cook D, Beaulieu WT, Mott IW *et al.* Production of the alkaloid swainsonine by a fungal endosymbiont of the Ascomycete order Chaetothyriales in the host *Ipomoea carnea*. *J Agric Food Chem* 2013; **61**(16):3797-803. doi: 10.1021/jf4008423.

210. Pischel I. Solanaceae and Convolvulaceae: secondary metabolites, biosynthesis, chemotaxonomy, biological and economic significance. *Journal of Ethnopharmacology* 2009; **123**(1):194. doi: 10.1016/j.jep.2009.02.021.

211. Francesc Viladomat CC, Jaume Bastida, Merce Galobardes *et al.* alkaloid screening of Catalonia (Spain) plants, I. *J Nat Prod* 1984; **47**(1):64-69.

212. Yun-Heng Shen S-HL, Rong-Tao Li, Quan-Bin Han *et al.* Coriatone and corianlactone, two novel sesquiterpenes from *Coriaria nepalensis*. *Org Lett* 2004; **6**(10):1593-1595.

213. L. Dinan TS, P. Whiting. On the distribution of phytoecdysteroids in plants. *Cell. Mol. Life Sci.* 2001; **58**:1121-1132.

214. Navindra P. Seeram RS, Amtabh Chandra, Muraleedharan G. Nair. Characterization, quantification, and bioactivities of anthocyanins in *Cornus* species. *J. Agric. Food Chem.* 2002; **50**:2519-2523.

215. Frank R. Stermitz REK. Iridoid glycosides of *Cornus canadensis*: a comparison with some other *Cornus* species. *Biochem Syst Ecol* 1998; **26**:845-849.

216. Pawlowska AM, Camangi F, Braca A. Quali-quantitative analysis of flavonoids of *Cornus mas* L. (Cornaceae) fruits. *Food Chemistry* 2010; **119**(3):1257-1261. doi: 10.1016/j.foodchem.2009.07.063.

217. Ren G, Hu Z-C, Xiang H-Y *et al.* Chemical constituents from the fruiting branches of *Artocarpus nanchuanensis* endemic to China. *Biochem Syst Ecol* 2013; **51**:98-100. doi: 10.1016/j.bse.2013.08.019.

218. Jeong Hee Kim HTH, Jan F. Stevensa. Alkaloids of some Asian *Sedum* species. *Phytochemistry* 1996; **41**(5):1319-1324.

219. Jan F. Stevens HTH, Roeland C.H.J. Van Ham, Elizabeth T. Elema *et al.* Distribution of alkaloids and tannins in the Crassulaceae. *Biochem Syst Ecol* 1995; **23**(2):157-165.

220. Jan F. Stevens HH, Elizabetht. Elema, Annabel Bolck. Flavonoid variation in eurasian *Sedum* and *Sempervivum*. *Phytochemistry* 1996; **41**(2):503-512.

221. JF Stevens HH, E Wollenweber. The systematic and evolutionary significance of exudate flavonoids in *Aeonium*. *Phytochemistry* 1995; **39**(4):805-813.

222. Qiu M. Chemotaxonomy of Cucurbitaceae. *Chin J Appl Environ BIol* 2005; **11**(6):673-685.

223. Gordien AY, Gray AI, Franzblau SG *et al.* Antimycobacterial terpenoids from *Juniperus communis* L. (Cuppressaceae). *J Ethnopharmacol* 2009; **126**(3):500-5. doi: 10.1016/j.jep.2009.09.007.

224. Yu-Mei Zhang N-HT, Yang Lu, Ying Chang *et al.* Chamobtusin A, a novel skeleton diterpenoid alkaloid from *Chamaecyparis obtusa* cv. *tetragon*. *Org Lett* 2007; **9**(22):4579-4581.

225. Sally Cowan BB, Alison A. Watson, Colin Bright *et al.* Lignans from *Cupressus lusitanica* (Cupressaceae). *Biochem Syst Ecol* 2001; **29**:109-111.

226. Hazel Sharp ZL, Barbara Bartholomew, Colin Bright *et al.* Totarol, totaradiol and ferruginol three diterpenes from Thujaplicata (Cupressaceae). *Biochem Syst Ecol* 2001; **29**:215-217.

227. Ragasa CY, Ng VAS, Agoo EMG *et al.* Chemical constituents of *Cycas vespertilio*. *Revista Brasileira de Farmacognosia* 2015; **25**(5):526-528. doi: 10.1016/j.bjp.2015.06.002.

228. Vincent Antonio S. Ng EMGA, Chien-Chang Shen, Consolacion Y. Ragasa. Secondary metabolites from *Cycas edentata*. *J. Pharm. Sci. & Res.* 2015; **7**(9):643-646.

229. Moawad A, Hetta M, Zjawiony JK *et al.* Two new dihydroamentoflavone glycosides from *Cycas revoluta*. *Nat Prod Res* 2014; **28**(1):41-7. doi: 10.1080/14786419.2013.832675.

230. Subhashini P, Dilipan E, Thangaradjou T *et al.* Bioactive natural products from marine angiosperms: abundance and functions. *Natural Products and Bioprospecting* 2013; **3**(4):129-136. doi: 10.1007/s13659-013-0043-6.

231. Papenbrock J. Highlights in seagrasses’ phylogeny, physiology, and metabolism: what makes them special? *ISRN Botany* 2012; **2012**:1-15. doi: 10.5402/2012/103892.

232. Qingcui Chu XT, Miao Lin, and Jiannong Ye. Electromigration profiles of *Cynomorium songaricum* based on capillary electrophoresis with amperometric detection. *J. Agric. Food Chem.* 2006; **54**(21):7979-7983.

233. Cordell GA, Quinn-Beattie ML, Farnsworth NR. The potential of alkaloids in drug discovery. *Phytother Res* 2001; **15**(3):183-205.

234. Manhart JR. Chemotaxonomy of the genus *Carex* (Cyperaceae). *Canadian Journal of Botany* 1990; **68**(7):1457-1461.

235. Ren Y, VanSchoiack A, Chai HB *et al.* Cytotoxic barrigenol-like triterpenoids from an extract of *Cyrilla racemiflora* housed in a repository. *J Nat Prod* 2015; **78**(10):2440-6. doi: 10.1021/acs.jnatprod.5b00532.

236. Lu Y-Y, Wang X-Y, Gao K *et al.* Chemical constituents from *Daphniphyllum macropodum*. *Biochem Syst Ecol* 2014; **57**:458-460. doi: 10.1016/j.bse.2014.10.007.

237. Tseng-chieng Huang T. Daphniphyllaceae. *Flora Malesiana* 1997; **13**:145-168.

238. Zhang H, Shyaula SL, Li JY *et al.* Hydroxylated Daphniphyllum Alkaloids from *Daphniphyllum himalense*. *J Nat Prod* 2015; **78**(11):2761-7. doi: 10.1021/acs.jnatprod.5b00741.

239. Xu JB, Zhang H, Gan LS *et al.* Logeracemin A, an anti-HIV Daphniphyllum alkaloid dimer with a new carbon skeleton from *Daphniphyllum longeracemosum*. *J Am Chem Soc* 2014; **136**(21):7631-3. doi: 10.1021/ja503995b.

240. El Bitar H, Nguyen VH, Gramain A *et al.* New alkaloids from *Daphniphyllum calycinum*. *J Nat Prod* 2004; **67**(7):1094-9. doi: 10.1021/np040038f.

241. Haiyun Bai LH. Study on the chemical constituents of *Daphniphyllum angustifolium*. *Helvetica chimica acta* 2006; **89**:884-894.

242. Liqiong Fang AI, Hee-Byung Chai, Qiuwen Mi *et al.* cytotoxic constituents from the stem bark of *Dichapetalum gelonioides* Collected in the Philippines. *J. Nat. Prod.* 2006; **69**:332-337.

243. Appiah AM. Phytochemical constituents and biological activity studies of the stem of *Dichapetalum Madagascariense*. *University of Ghana* 2013.

244. Waterman PG. The chemical systematics of alkaloids: A review emphasising the contribution of Robert Hegnauer. *Biochem Syst Ecol* 1999; **27**:395-406.

245. lwashina T. The structure and distribution of the flavonoids in plants. *J. Plant Res.* 2000; **113**:287-299.

246. Soares GLG. Analysis of flavone-flavonolratio in Dicotyledoneae. *Botanical Journal of the Linnean Society* 2001; **135**:61-66. doi: 10.1006/boj1.2001.0357.

247. Jalil J, Sabandar CW, Ahmat N *et al.* Inhibitory effect of triterpenoids from *Dillenia serrata* (Dilleniaceae) on prostaglandin E2 production and quantitative HPLC analysis of its koetjapic acid and betulinic acid contents. *Molecules* 2015; **20**(2):3206-20. doi: 10.3390/molecules20023206.

248. Gerhard Bringmann MD, Helene Kopff, Heiko Rischer *et al.* ent-Dioncophylleine A and related dehydrogenated naphthylisoquinoline alkaloids, the first asian Dioncophyllaceae-Type alkaloids, from the “new” plant species *Ancistrocladus benomensis*. *J. Nat. Prod.* 2005; **68**:686-690.

249. Ali Z, Smillie TJ, Khan IA. Cholestane steroid glycosides from the rhizomes of *Dioscorea villosa* (wild yam). *Carbohydr Res* 2013; **370**:86-91. doi: 10.1016/j.carres.2012.12.022.

250. Lin JT, Yang DJ. Determination of steroidal saponins in different organs of yam (*Dioscorea pseudojaponica* Yamamoto). *Food Chem* 2008; **108**(3):1068-74. doi: 10.1016/j.foodchem.2007.11.041.

251. Yang D-J, Lu T-J, Hwang LS. Effect of endogenous glycosidase on stability of steroidal saponins in Taiwanese yam (*Dioscorea pseudojaponica* Yamamoto) during drying processes. *Food Chemistry* 2009; **113**(1):155-159. doi: 10.1016/j.foodchem.2008.07.060.

252. Yang D-J, Lin J-T. Effects of different storage conditions on steroidal saponinsin yam (*Dioscorea pseudojaponica* Yamamoto) tubers. *Food Chemistry* 2008; **110**(3):670-677. doi: 10.1016/j.foodchem.2008.02.061.

253. Liscombe DK, Macleod BP, Loukanina N *et al.* Evidence for the monophyletic evolution of benzylisoquinoline alkaloid biosynthesis in angiosperms. *Phytochemistry* 2005; **66**(11):1374-93. doi: 10.1016/j.phytochem.2005.04.029.

254. Sautour M, Canon F, Miyamoto T *et al.* A new ecdysteroid and other constituents from two *Dioscorea* species. *Biochem Syst Ecol* 2008; **36**(7):559-563. doi: 10.1016/j.bse.2008.03.002.

255. Shriram V, Jahagirdar S, Latha C *et al.* A potential plasmid-curing agent, 8-epidiosbulbin E acetate, from *Dioscorea bulbifera* L. against multidrug-resistant bacteria. *Int J Antimicrob Agents* 2008; **32**(5):405-10. doi: 10.1016/j.ijantimicag.2008.05.013.

256. Sautour M, Miyamoto T, Lacaille-Dubois MA. Steroidal saponins and flavan-3-ol glycosides from *Dioscorea villosa*. *Biochem Syst Ecol* 2006; **34**(1):60-63. doi: 10.1016/j.bse.2005.07.007.

257. Remy Bertrand Teponno ALT, Hyun Ju-Jungb, Jung-Hwan Namb *et al.* Three new clerodane diterpenoids from the bulbils of *Dioscorea* *bulbifera* L. var. *sativa*. *Helvetica Chimica Acta* 2007; **90**:1599-1605.

258. Joshi K. Chemotaxonomic investigation of *Cotylelobium* species (Dipterocarpaceae) using flavonoid analysis. *Scientific World* 2008; **6**(6):24-26.

259. Wibowo A, Ahmat N. Chemotaxonomic significance of oligostilbenoids isolated from *Dryobalanops* in the taxonomic of Dipterocarpaceae. *Biochem Syst Ecol* 2015; **59**:31-35. doi: 10.1016/j.bse.2014.12.024.

260. Wibowo A, Ahmat N, Hamzah AS *et al.* Identification and biological activity of secondary metabolites from *Dryobalanops beccarii*. *Phytochemistry Letters* 2014; **9**:117-122. doi: 10.1016/j.phytol.2014.05.001.

261. Joshi K, Seneviratne GI, Senanayake SP. Leaf flavonoid aglycone patterns in the species of Dipterocarpaceae in Sri Lanka. *Biochem Syst Ecol* 2004; **32**(3):329-336. doi: 10.1016/j.bse.2003.06.001.

262. Joshi K. Leaf flavonoid patterns in *Dipterocarpus* and *Hopea* (Dipterocarpaceae). *Botanical Journal of the Linnean Society* 2003; **143**:43-46.

263. Atun S, Achmad SA, Niwa M *et al.* Oligostilbenoids from *Hopea mengarawan* (Dipterocarpaceae). *Biochem Syst Ecol* 2006; **34**(8):642-644. doi: 10.1016/j.bse.2006.02.008.

264. Tukiran, Achmad SA, Hakim EH *et al.* Oligostilbenoids from *Shorea balangeran*. *Biochem Syst Ecol* 2005; **33**(6):631-634. doi: 10.1016/j.bse.2004.10.016.

265. Atun S, Achmad SA, Ghisalberti EL *et al.* Oligostilbenoids from *Vatica umbonata* (Dipterocarpaceae). *Biochem Syst Ecol* 2004; **32**(11):1051-1053. doi: 10.1016/j.bse.2004.04.001.

266. Wibowo A, Ahmat N, Hamzah AS *et al.* Resveratrol oligomers from the stem bark of *Dryobalanops aromatica*. *Biochem Syst Ecol* 2012; **40**:62-64. doi: 10.1016/j.bse.2011.09.013.

267. Braunberger C, Zehl M, Conrad J *et al.* Flavonoids as chemotaxonomic markers in the genus *Drosera*. *Phytochemistry* 2015; **118**:74-82. doi: 10.1016/j.phytochem.2015.08.017.

268. Jaromir Budzianowskia AB, Krystyna Kromer. Naphthalene glucoside and other phenolics from the shoot and callus cultures of *Drosophyllum lusitanicum*. *Phytochemistry* 2002; **61**:421-425.

269. Paul A. Egana FvdK. Phytochemistry of the carnivorous sundew genus *Drosera* (Droseraceae). *Chemistry & Biodiversity* 2013; **10**:1774-1790.

270. U.V. Mallavadhani AKP, Y.R. Rao. Pharmacology and chemotaxonomy of *Diospyros*. *Phytochemistry* 1998; **49**(4):901-951.

271. Jani M. Koponen AMH, Pirjo H. Mattila, A. Riitta Törrönen. Contents of anthocyanins and ellagitannins in selected foods consumed in Finland. *J. Agric. Food Chem.* 2007; **55**:1612-1619.

272. Moilanen J, Koskinen P, Salminen JP. Distribution and content of ellagitannins in Finnish plant species. *Phytochemistry* 2015; **116**:188-97. doi: 10.1016/j.phytochem.2015.03.002.

273. Kaisu R. Määttä-Riihinen AK-E, Pirjo H. Mattila, Ana M. González-Paramás *et al.* Distribution and contents of phenolic compounds in eighteen scandinavian berry species. *J. Agric. Food Chem.* 2004; **52**:4477-4486.

274. Fang R, Veitch NC, Kite GC *et al.* Enhanced profiling of flavonol glycosides in the fruits of sea buckthorn (*Hippophae rhamnoides*). *J Agric Food Chem* 2013; **61**(16):3868-75. doi: 10.1021/jf304604v.

275. Cao SG, Tanaka T, Mizuno M *et al.* Flavonol glycosides from *Elaeagnus lanceollata* (Elaeagnaceae). *Nat Prod Lett* 2001; **15**(4):211-6. doi: 10.1080/10575630108041283.

276. Leskinen HM, Suomela JP, Yang B *et al.* Regioisomer compositions of vaccenic and oleic acid containing triacylglycerols in sea buckthorn (*Hippophae rhamnoides*) pulp oils: influence of origin and weather conditions. *J Agric Food Chem* 2010; **58**(1):537-45. doi: 10.1021/jf902679v.

277. Elkhateeb A, Subeki, Takahashi K *et al.* Anti-babesial ellagic acid rhamnosides from the bark of *Elaeocarpus parvifolius*. *Phytochemistry* 2005; **66**(21):2577-80. doi: 10.1016/j.phytochem.2005.08.020.

278. Hasegawa T, Takano F, Takata T *et al.* Bioactive monoterpene glycosides conjugated with gallic acid from the leaves of *Eucalyptus globulus*. *Phytochemistry* 2008; **69**(3):747-53. doi: 10.1016/j.phytochem.2007.08.030.

279. Miller RE, Stewart M, Capon RJ *et al.* A galloylated cyanogenic glycoside from the Australian endemic rainforest tree *Elaeocarpus sericopetalus* (Elaeocarpaceae). *Phytochemistry* 2006; **67**(13):1365-71. doi: 10.1016/j.phytochem.2006.03.022.

280. Peter L. Katavic DAV, Topul Rali, Anthony R. Carroll. Habbemines A and B, pyrrolidine alkaloids with human δ-opioid receptor binding affinity from the leaves of *Elaeocarpus habbemensis*. *J. Nat. Prod.* 2007; **70**:866-868.

281. Peter L. Katavic DAV, Topul Rali, Anthony R. Carroll. Indolizidine alkaloids with δ-opioid receptor binding affinity from the leaves of *Elaeocarpus fuscoides*. *J Nat Prod* 2007; **70**(5):872-875.

282. Ibragic S, Sofic E. Chemical composition of various *Ephedra* species. *Bosn J Basic Med Sci* 2015; **15**(3):21-7. doi: 10.17305/bjbms.2015.539.

283. Zhang D, Deng A-J, Ma L *et al.* N-Substituted acetamide glycosides from the stems of *Ephedra sinica*. *Phytochemistry Letters* 2015; **12**:320-327. doi: 10.1016/j.phytol.2015.04.014.

284. Al-Khalil S; Transtorine, a new quinoline alkaloid from *Ephedra transitoria*. *J. Nat. Prod.* 1998; **61**:262-263.

285. Monschein M, Iglesias Neira J, Kunert O *et al.* Phytochemistry of heather (*Calluna vulgaris* (L.) Hull) and its altitudinal alteration. *Phytochemistry Reviews* 2009; **9**(2):205-215. doi: 10.1007/s11101-009-9153-5.

286. Nascimento Rocha MEd, Figueiredo MR, Coelho Kaplan MA *et al.* Chemotaxonomy of the Ericales. *Biochem Syst Ecol* 2015; **61**:441-449. doi: 10.1016/j.bse.2015.07.019.

287. Qiao X, Ye G, Liu CF *et al.* Chemical analysis of *Eriocaulon buergerianum* and adulterating species by high-performance liquid chromatography with diode array detection and electrospray ionization tandem mass spectrometry. *J Pharm Biomed Anal* 2012; **57**:133-42. doi: 10.1016/j.jpba.2011.08.033.

288. Anne Lı´gia Dokkedala LCdS, Paulo Takeo Sanoc, Wagner Vilegas. Chemistry in Eriocaulaceae. *Zeitschrift für Naturforschung C.* 2008; **63**(3-4):169-175.

289. Dokkedal AL, Sano PT, Vilegas W. Chemistry in *Paepalanthus* and taxonomic implications. *Biochem Syst Ecol* 2004; **32**(5):503-504. doi: 10.1016/j.bse.2003.11.004.

290. Fabio Donisete Pezzuto de Andrade LR, Cosimo Pizza, Paulo T. Sano *et al.* Flavonol glycosides and a naphthopyranone glycoside from *Paepalanthus macropodus* (Eriocaulaceae). *Biochem Syst Ecol* 2002; **30**:275-277.

291. do Amaral FP, Napolitano A, Masullo M *et al.* HPLC-ESIMS(n) profiling, isolation, structural elucidation, and evaluation of the antioxidant potential of phenolics from *Paepalanthus geniculatus*. *J Nat Prod* 2012; **75**(4):547-56. doi: 10.1021/np200604k.

292. Zuanazzi JAS, Vale´ria Tremea, Limberger RP *et al.* Alkaloids of *Erythroxylum* (Erythroxylaceae) species from Southern Brazil. *Biochem Syst Ecol* 2001; **29**:819-825.

293. Brock A, Bieri S, Christen P *et al.* Calystegines in wild and cultivated *Erythroxylum* species. *Phytochemistry* 2005; **66**(11):1231-40. doi: 10.1016/j.phytochem.2005.04.017.

294. Bieri S, Brachet A, Veuthey JL *et al.* Cocaine distribution in wild *Erythroxylum* species. *J Ethnopharmacol* 2006; **103**(3):439-47. doi: 10.1016/j.jep.2005.08.021.

295. Bittrich V. Erythroxylaceae. doi: 10.1007/978-3-642-39417-1_9, *Springer-Verlag*.

296. González-Guevara JL, Vélez-Castro H, González-García KL *et al.* Flavonoid glycosides from Cuban *Erythroxylum* species. *Biochem Syst Ecol* 2006; **34**(6):539-542. doi: 10.1016/j.bse.2006.01.003.

297. JP Chávez IDS, FG Cruz, JM David. Flavonoids and triterpene ester derivatives from *Erythroxylum leal* costae. *Phytocheraistry* 1996; **41**(3):941-943.

298. Barreiros ML, David JM, de Queiroz LP *et al.* Flavonoids and triterpenes from leaves of *Erythroxylum nummularia*. *Biochem Syst Ecol* 2005; **33**(5):537-540. doi: 10.1016/j.bse.2004.10.007.

299. Costa dos Santos C, Lima MAS, Silveira ER. Micromolecular secondary metabolites of *Erythroxylum barbatum*. *Biochem Syst Ecol* 2003; **31**(6):661-664. doi: 10.1016/s0305-1978(02)00249-1.

300. Zanolari B, Wolfender JL, Guilet D *et al.* On-line identification of tropane alkaloids from *Erythroxylum vacciniifolium* by liquid chromatography–UV detection–multiple mass spectrometry and liquid chromatography–nuclear magnetic resonance spectrometry. *Journal of Chromatography A* 2003; **1020**(1):75-89. doi: 10.1016/j.chroma.2003.08.052.

301. Barreiros ML, David JP, David JM *et al.* Ryanodane diterpenes from two *Erythroxylum* species. *Phytochemistry* 2007; **68**(13):1735-9. doi: 10.1016/j.phytochem.2007.05.007.

302. M. Sauvain CR, C. Moretti *et al.* A study of the chemical composition of *Erythroxylum coca* var. *coca* leaves collected in two ecological regions of Bolivia. *Journal of Ethnopharmacology* 1997; **56**:179-191.

303. Levrier C, Sadowski MC, Nelson CC *et al.* Cytotoxic C20 Diterpenoid alkaloids from the Australian endemic rainforest plant *Anopterus macleayanus*. *J Nat Prod* 2015; **78**(12):2908-16. doi: 10.1021/acs.jnatprod.5b00509.

304. Bai MM, Shi W, Tian JM *et al.* Soluble epoxide hydrolase inhibitory and anti-inflammatory components from the leaves of *Eucommia ulmoides* Oliver (duzhong). *J Agric Food Chem* 2015; **63**(8):2198-205. doi: 10.1021/acs.jafc.5b00055.

305. Zhang Y, Peng M, Liu L *et al.* Screening, identification, and potential interaction of active compounds from *Eucommia ulmodies* leaves binding with bovine serum albumin. *J Agric Food Chem* 2012; **60**(12):3119-25. doi: 10.1021/jf205135w.

306. Chika Takamura TH, Taro Ueda, Masateru Ono *et al.* Iridoids from the green leaves of *Eucommia ulmoides*. *J. Nat. Prod.* 2007; **70**:1312-1316.

307. Cardona Zuleta LM, Cavalheiro AJ, Siqueira Silva DH *et al.* Seco-iridoids from *Calycophyllum spruceanum* (Rubiaceae). *Phytochemistry* 2003; **64**(2):549-553. doi: 10.1016/s0031-9422(03)00153-5.

308. Hu Z, Lai Y, Zhang J *et al.* Phytochemical and chemotaxonomic studies on *Phyllanthus urinaria*. *Biochem Syst Ecol* 2014; **56**:60-64. doi: 10.1016/j.bse.2014.04.016.

309. Rivière C, Nguyen Thi Hong V, Tran Hong Q *et al.* Mallotus species from Vietnamese mountainous areas: phytochemistry and pharmacological activities. *Phytochemistry Reviews* 2009; **9**(2):217-253. doi: 10.1007/s11101-009-9152-6.

310. Zhao W, Wolfender J-L, Mavi S *et al.* Diterpenes and sterols from *Neoboutonia melleri* (Euphorbiaceae). *Phytochemistry* 1998; **48**(7):1173-1177.

311. Reiersen B, Kiremire BT, Byamukama R *et al.* Anthocyanins acylated with gallic acid from chenille plant, *Acalypha hispida*. *Phytochemistry* 2003; **64**(4):867-871. doi: 10.1016/s0031-9422(03)00494-1.

312. Daniel J. Crawford TFS, Mario Silva O. Leaf Flavonoid chemistry and the relationships of the Lactoridaceae. *P1. Syst. Evol.* 1986; **153**:133-139.

313. Takao Konoshima MT, Mutsuo Kozuka. Studies on inhibitors of skin-tumor promotion, i. inhibitory effects of triterpenes from *Euptelea polyandra* on Epstein-Barr Virus Activation. *J Nat Prod* 1987; **50**(6):1167-1170.

314. Takao Konoshima TM, Midori Takasaki, Johji Yamahara *et al.* Constituents of the bark of *Euptelea polyandra*. *J. Nat. Prod.* 1985; **48**(4):683-684.

315. Yang X, Zhou R. Studies on chemotaxonomy of *Mucuna* and *Stizolobium*. *J China Pharmaceutical University* 1992; **23**(2):74-76.

316. Ionkova I, Shkondrov A, Krasteva I *et al.* Recent progress in phytochemistry, pharmacology and biotechnology of *Astragalus* saponins. *Phytochemistry Reviews* 2014; **13**(2):343-374. doi: 10.1007/s11101-014-9347-3.

317. Kodithuwakku Kankanange Indika Upali Arunakumara BCW, Siripala Subasinghe, Min-Ho Yoon. *Pterocarpus santalinus* Linn. f. (Rath handun): A review of its botany, uses, phytochemistry and pharmacology. *Journal of the Korean Society for Applied Biological Chemistry* 2011; **54**(4):496-500. doi: 10.3839/jksabc.2011.076.

318. Veitch NC, Kite GC, Lewis GP. Flavonol pentaglycosides of *Cordyla* (Leguminosae: Papilionoideae: Swartzieae): distribution and taxonomic implications. *Phytochemistry* 2008; **69**(12):2329-35. doi: 10.1016/j.phytochem.2008.05.026.

319. Giuseppe Dada AC. Lignan glycosides from the heartwood of european oak *Quercus petraea*. *J Nat Prod* 1989; **52**(6):1327-1330.

320. Martin Konig ES. Ellagitannins and Complex Tannins from *Quercus petraea* Bark. *J Nat Prod* 1994; **57**(10):1411-1415.

321. Loreto F. Distribution of isoprenoid emitters in the *Quercus* genus around the world: chemo-taxonomical implications and evolutionary considerations based on the ecological function of the trait. *Perspectives in Plant Ecology, Evolution and Systematics* 2002; **5**(3):185-192. doi: 10.1078/1433-8319-00033.

322. Karioti A, Sokovic M, Ciric A *et al.* Antimicrobial properties of *Quercus ilex* L. proanthocyanidin dimers and simple phenolics: evaluation of their synergistic activity with conventional antimicrobials and prediction of their pharmacokinetic profile. *J Agric Food Chem* 2011; **59**(12):6412-22. doi: 10.1021/jf2011535.

323. Yeh Schun GAC. Cytotoxic Steroids of *Gelsemium sempervirens*. *J Nat Prod* 1987; **50**(2):195-198.

324. Magora HB, Rahman MM, Gray AI *et al.* Swertiamarin from *Enicostemma axillare* subsp. *axillare* (Gentianaceae). *Biochem Syst Ecol* 2003; **31**(5):553-555. doi: 10.1016/s0305-1978(02)00200-4.

325. Chen G, Wei S-H, Yu C-Y. Secoiridoids from the roots of *Gentiana straminea*. *Biochem Syst Ecol* 2009; **37**(6):766-771. doi: 10.1016/j.bse.2009.12.004.

326. Anyanwu GO, Nisar Ur R, Onyeneke CE *et al.* Medicinal plants of the genus *Anthocleista*-A review of their ethnobotany, phytochemistry and pharmacology. *J Ethnopharmacol* 2015; **175**:648-67. doi: 10.1016/j.jep.2015.09.032.

327. Jan J. Rybczyński MRD, Anna Mikuła. The Gentianaceae -Volume 2: Biotechnology and Applications. *Springer Heidelberg New York Dordrecht London* 2015; **14,15,16**:319-399.

328. S. R. Jensen JS. Chemotaxonomy and pharmacology of Gentianaceae. *Gentianaceae-Systematic and Natural history. Struwe L. and Albert V. eds. Cambridge University Press* 2002; **6**:573-661.

329. Wu L-H, Annie Bligh SW, Leon CJ *et al.* Chemotaxonomically significant roburic acid from Section Cruciata of *Gentiana*. *Biochem Syst Ecol* 2012; **43**:152-155. doi: 10.1016/j.bse.2012.03.008.

330. Li W, Zhou W, Shim SH *et al.* Chemical constituents of the rhizomes and roots of *Gentiana scabra* (Gentianaceae). *Biochem Syst Ecol* 2015; **61**:169-174. doi: 10.1016/j.bse.2015.06.024.

331. Li L, Li MH, Zhang N *et al.* Chemical constituents from *Lomatogonium carinthiacum* (Gentianaceae). *Biochem Syst Ecol* 2011; **39**(4-6):766-768. doi: 10.1016/j.bse.2011.07.002.

332. Cui Z-H, Li Y, Wang Z-W *et al.* Chemical constituents from *Gentianopsis barbata* var. *sinensis* Ma (Gentianaceae). *Biochem Syst Ecol* 2013; **47**:101-103. doi: 10.1016/j.bse.2012.11.003.

333. Kumarasamy Y, Nahar L, Cox PJ *et al.* Bioactivity of secoiridoid glycosides from *Centaurium erythraea*. *Phytomedicine* 2003; **10**(4):344-7. doi: 10.1078/094471103322004857.

334. Lis-Balchin M, Guittonneau G-G. Preliminary investigations on the presence of alkaloids in the genus *Erodium* L'Her. (Geraniaceae). *Acta Botanica Gallica* 1995; **142**(1):31-35. doi: 10.1080/12538078.1995.10515689.

335. Peter F. Yeo HW-K. The chemotaxonomy of *Geranium* (Geraniaceae). *Plant Systematics and Evolution* 1990; **173**:1-15.

336. Lis-Balchin MT. A chemotaxonomic reappraisal of the Section Ciconium *Pelargonium* (Geraniaceae). *South African Journal of Botany* 1996; **62**(5):277-279. doi: 10.1016/s0254-6299(15)30657-8.

337. Stephanie Ivancheva AP. A chemosystematic study of eleven Geranium species. *Biochem Syst Ecol* 2000; **28**:255-260.

338. Wu N, Zu Y, Fu Y *et al.* Antioxidant activities and xanthine oxidase inhibitory effects of extracts and main polyphenolic compounds obtained from *Geranium sibiricum* L. *J Agric Food Chem* 2010; **58**(8):4737-43. doi: 10.1021/jf904593n.

339. Xiang-Hai Cai, Xiao-Dong Luo, Jun Zhou *et al.* Quinones from *Chirita eburnea*. *J Nat Prod* 2005; **68**(5):797-799.

340. Maria Helena Verdan AB, Eduardo Lemos de Sa *et al.* Lactones and quinones from the tubers of *Sinningia aggregata*. *J Nat Prod* 2010; **73**(8):1434-1437.

341. Maria Helena Verdan MElAS. Secondary metabolites and biological properties of Gesneriaceae species. *Chemistry & Biodiversity* 2012; **9**:2701-2731.

342. Jensen SR. Caffeoyl phenylethanoid glycosides in *Sanango racemosum* and in the Gesneriaceae. *Phytochemistry* 1996; **43**(4):777-783.

343. Ji Suk Lee YSC, Eun Jung Park, Jinwoong Kim *et al.* Phospholipase Cγ1 inhibitory principles from the sarcotestas of *Ginkgo biloba*. 1998; **61**:867-871.

344. Eckhard Leistner CD. *Ginkgo biloba* and Ginkgotoxin. *J. Nat. Prod.* 2010(73):86-92.

345. Sergei Bolshakov SVD, John Decatur, and Koji Nakanishi. A concise synthesis of ginkgolide m, a minor component of a terpene trilactone fraction from *Ginkgo biloba* Roots. *J. Nat. Prod.* 2006; **69**:429-431.

346. Buffeteau T, Cavagnat D, Bisson J *et al.* Unambiguous determination of the absolute configuration of dimeric stilbene glucosides from the rhizomes of *Gnetum africanum*. *J Nat Prod* 2014; **77**(8):1981-5. doi: 10.1021/np500427v.

347. Ota H, Akishita M, Tani H *et al.* trans-Resveratrol in *Gnetum gnemon* protects against oxidative-stress-induced endothelial senescence. *J Nat Prod* 2013; **76**(7):1242-7. doi: 10.1021/np300841v.

348. Eishin Kato YT, Fujio Sakan. Stilbenoids isolated from the seeds of melinjo (*Gnetum gnemon* L.) and their biological activity. *J Agr Food Chem*2009; **57**:2544–2549.

349. Zulfiqar Ali TT, Ibrahim Iliya *et al.* Phenolic constituents of *Gnetum klossii*. *J Nat Prod* 2003; **66**(4):558-560.

350. Riviere C, Pawlus AD, Merillon JM. Natural stilbenoids: distribution in the plant kingdom and chemotaxonomic interest in Vitaceae. *Nat Prod Rep* 2012; **29**(11):1317-33. doi: 10.1039/c2np20049j.

351. Qun Xu ML. Benzylisoquinoline alkaloids from *Gnetum parvifolium*. *J Nat Prod* 1999; **62**(7):1025-1027.

352. Martin F, Grkovic T, Sykes ML *et al.* Alkaloids from the Chinese vine *Gnetum montanum*. *J Nat Prod* 2011; **74**(11):2425-30. doi: 10.1021/np200700f.

353. Tamara Savchenko PW, Vladimir Sik *et al.* Distribution and identities of phytoecdysteroids in the genus *Briza* (Gramineae). *Biochem Syst Ecol* 1999; **26**:781—791.

354. Yang B, Zheng J, Laaksonen O *et al.* Effects of latitude and weather conditions on phenolic compounds in currant (*Ribes* spp.) cultivars. *J Agric Food Chem* 2013; **61**(14):3517-32. doi: 10.1021/jf4000456.

355. Sari H. Hakkinen SOK, I. Marina Heinonen *et al.* Content of the Flavonols Quercetin, Myricetin, and Kaempferol in 25 Edible Berries. *J Agr Food Chem*1999; **47**:2274-2279.

356. Stevic T, Savikin K, Ristic M *et al.* Composition and antimicrobial activity of the essential oil of the leaves of black currant (*Ribes nigrum* L.) cultivar Čačanska crna. *Journal of the Serbian Chemical Society* 2010; **75**(1):35-43. doi: 10.2298/jsc1001035s.

357. John Mexal WCM. Chemotaxonomy of *Ribes*. *The Southwestern Naturalist* 1977; **21**(4):523-530.

358. Doyle MF, Scogin R. A comparative phytochemical profile of the Gunneraceae. *New Zealand Journal of Botany* 1988; **26**(4):493-496. doi: 10.1080/0028825x.1988.10410656.

359. Cardini F, Bonzi LM. Carotenoid composition and its chemotaxonomic significance in leaves of ten species of the genus *Ceratozamia* (Cycads). *Journal of Plant Physiology* 2005; **162**(5):517-528. doi: 10.1016/j.jplph.2004.06.007.

360. Fang J, Paetz C, Hölscher D *et al.* Phenylphenalenones and related natural products from *Wachendorfia thyrsiflora* L. *Phytochemistry Letters* 2011; **4**(2):203-208. doi: 10.1016/j.phytol.2011.03.006.

361. Opitz S, Otalvaro F, Echeverri F *et al.* Isomeric oxabenzochrysenones from *Musa acuminata* and *Wachendorfia thyrsiflora*. *Nat Prod Lett* 2002; **16**(5):335-8. doi: 10.1080/10575630290033079.

362. Brkljaca R, Urban S. HPLC-NMR and HPLC-MS profiling and bioassay-guided identification of secondary metabolites from the australian plant *Haemodorum spicatum*. *J Nat Prod* 2015; **78**(7):1486-94. doi: 10.1021/np500905g.

363. Opitz S, Schnitzler JP, Hause B *et al.* Histochemical analysis of phenylphenalenone-related compounds in *Xiphidium caeruleum* (Haemodoraceae). *Planta* 2003; **216**(5):881-9. doi: 10.1007/s00425-002-0941-z.

364. Fang J, Holscher D, Schneider B. Co-occurrence of phenylphenalenones and flavonoids in *Xiphidium caeruleum* Aubl. flowers. *Phytochemistry* 2012; **82**:143-8. doi: 10.1016/j.phytochem.2012.07.005.

365. Keylor MH, Matsuura BS, Stephenson CR. Chemistry and biology of resveratrol-derived natural products. *Chem Rev* 2015; **115**(17):8976-9027. doi: 10.1021/cr500689b.

366. Iwashina T, Kitajima J, Takemura T. Flavonoids from the leaves of six Corylopsis species (Hamamelidaceae). *Biochem Syst Ecol* 2012; **44**:361-363. doi: 10.1016/j.bse.2012.06.017.

367. Pereira CAB, Oliveira FM, Conserva LM *et al.* Cinnamoyltyramine derivatives and other constituents from *Sparattanthelium tupiniquinorum* (Hernandiaceae). *Biochem Syst Ecol* 2007; **35**(9):637-639. doi: 10.1016/j.bse.2007.03.014.

368. Tchouya G, Obiang G, Bongui J-b *et al.* Phytochemical study of *Sacoglottis gabonensis* (Baill.) Urb. Isolation of bioactive compounds from the stem bark. *American Chemical Science Journal* 2016; **11**(4):1-5. doi: 10.9734/acsj/2016/22314.

369. Gousiadou C, Li H-Q, Gotfredsen C *et al.* Iridoids in Hydrangeaceae. *Biochem Syst Ecol* 2016; **64**:122-130. doi: 10.1016/j.bse.2015.12.002.

370. Qi S-H, Huang L-S, He F *et al.* Phytochemical and chemotaxonomic investigation of seagrass *Thalassia hemprichii* (Ehrenb.) Aschers (Hydrocharitaceae). *Biochem Syst Ecol* 2012; **43**:128-131. doi: 10.1016/j.bse.2012.03.006.

371. Nina Rønsted HS, Søren, Rosendal Jensen PM. Chlorogenic acid from three species of *Hydrostachys* (Hydrostachyaceae). *Biochem Syst Ecol* 2002; **30**:1105-1108.

372. Yin Z-Q, Wang Y, Ye W-C *et al.* Chemical constituents of *Hypericum perforatum* (St. John’s wort) growing in China. *Biochem Syst Ecol* 2004; **32**(5):521-523. doi: 10.1016/j.bse.2003.10.010.

373. Nogueira T, Marcelo-Curto MJ, Figueiredo AC *et al.* Chemotaxonomy of *Hypericum* genus from Portugal: Geographical distribution and essential oils composition of *Hypericum perfoliatum*, *Hypericum humifusum*, *Hypericum linarifolium* and *Hypericum pulchrum*. *Biochem Syst Ecol* 2008; **36**(1):40-50. doi: 10.1016/j.bse.2007.07.004.

374. Smelcerovic A, Spiteller M, Ligon AP *et al.* Essential oil composition of *Hypericum* L. species from Southeastern Serbia and their chemotaxonomy. *Biochem Syst Ecol* 2007; **35**(2):99-113. doi: 10.1016/j.bse.2006.09.012.

375. Hosni K, Msaâda K, Ben Taârit M *et al.* Essential oil composition of *Hypericum perfoliatum* L. and *Hypericum tomentosum* L. growing wild in Tunisia. *Industrial Crops and Products* 2008; **27**(3):308-314. doi: 10.1016/j.indcrop.2007.11.004.

376. Tatsis EC, Boeren S, Exarchou V *et al.* Identification of the major constituents of *Hypericum perforatum* by LC/SPE/NMR and/or LC/MS. *Phytochemistry* 2007; **68**(3):383-93. doi: 10.1016/j.phytochem.2006.11.026.

377. Epifano F, Fiorito S, Genovese S. Phytochemistry and pharmacognosy of the genus *Psorospermum*. *Phytochemistry Reviews* 2013; **12**(4):673-684. doi: 10.1007/s11101-013-9274-8.

378. Koorbanally C, Crouch NR, Langlois A *et al.* Homoisoflavanones and spirocyclic nortriterpenoids from three *Eucomis* species: *E. comosa*, *E. schijffii* and *E. pallidiflora* subsp. *pole-evansii* (Hyacinthaceae). *South African Journal of Botany* 2006; **72**(3):428-433. doi: 10.1016/j.sajb.2005.12.006.

379. Mulholland DA, Crouch NR, Koorbanally C *et al.* Infraspecific chemical variation in *Scilla zebrina* (Hyacinthaceae). *Biochem Syst Ecol* 2006; **34**(3):251-255. doi: 10.1016/j.bse.2005.10.005.

380. Koorbanally C, Mulholland DA, Crouch NR. Norlignans and homoisoflavanones from two South African *Drimiopsis* species (Hyacinthaceae: Hyacinthoideae). *Biochem Syst Ecol* 2006; **34**(7):588-592. doi: 10.1016/j.bse.2005.12.011.

381. Tracy Pohl CK, Neil R. Crouch, Dulcie A. Mulholland. Secondary metabolites of *Scilla plumbea*, *Ledebouria cooperi* and *Ledebouria ovatifolia* (Hyacinthaceae). *Biochem Syst Ecol* 2001; **29**:857-860.

382. Akihito Yokosuka KS, Takao Yamori, Yoshihiro Mimaki. Triterpene glycosides from *Curculigo orchioides* and their cytotoxic activity. *J Nat Prod* 2010; **73**(6):1102-1106.

383. Zhao M, Onakpa MM, Chen WL *et al.* 17-Norpimaranes and (9betaH)-17-Norpimaranes from the Tuber of *Icacina trichantha*. *J Nat Prod* 2015; **78**(4):789-96. doi: 10.1021/np5010328.

384. Guo D-Y, Ling T-J, Cai X-H. Chemical constituents of *Nothapodytes pittosporoides* (Icacinaceae). *Biochem Syst Ecol* 2015; **61**:293-296. doi: 10.1016/j.bse.2015.06.039.

385. Noormawati Haron STP. Distribution and taxonomic significance of flavonoids in the Olacaceae and Icacinaceae. *Biochem Syst Ecol* 1997; **25**(3):263-265.

386. Cong HJ, Zhao Q, Zhang SW *et al.* Terpenoid indole alkaloids from *Mappianthus iodoides* Hand.-Mazz. *Phytochemistry* 2014; **100**:76-85. doi: 10.1016/j.phytochem.2014.01.004.

387. Mizuno T, Okuyama Y, Iwashina T. Phenolic compounds from *Iris rossii*, and their chemotaxonomic and systematic significance. *Biochem Syst Ecol* 2012; **44**:157-160. doi: 10.1016/j.bse.2012.04.022.

388. Zhang Z, Liao L, Moore J *et al.* Antioxidant phenolic compounds from walnut kernels (*Juglans regia* L.). *Food Chemistry* 2009; **113**(1):160-165. doi: 10.1016/j.foodchem.2008.07.061.

389. Wu Y, Li Y-Y, Wu X *et al.* Chemical constituents from *Cyclocarya paliurus* (Batal.) Iljinsk. *Biochem Syst Ecol* 2014; **57**:216-220. doi: 10.1016/j.bse.2014.08.022.

390. Wang J, Liu J, Wen Q *et al.* Chemical constituents from the aerial parts of *Juncus setchuensis*. *Biochem Syst Ecol* 2010; **38**(5):1039-1041. doi: 10.1016/j.bse.2010.10.008.

391. Li ZB, Bao YM, Chen HB *et al.* A cytotoxic compound from the leaves of *Juglans mandshurica*. *Chinese Chemical Letters* 2007; **18**(7):846-848. doi: 10.1016/j.cclet.2007.05.043.

392. Si C-L, Xu J, Lu Y-Y *et al.* Hydrolysable tannins from *Juglans sigillata* stem barks. *Biochem Syst Ecol* 2011; **39**(3):225-227. doi: 10.1016/j.bse.2011.02.010.

393. Zhi-Hong Jiang TT, Isao Kouno. A lupane triterpene and two triterpene caffeates from *Rhoiptelea chiliantha* (Rhoipteleaceae). *Phytochemistry* 1995; **40**(4):1223-1226.

394. Zhang Y-W, Lin H, Bao Y-L *et al.* A new triterpenoid and other constituents from the stem bark of *Juglans mandshurica*. *Biochem Syst Ecol* 2012; **44**:136-140. doi: 10.1016/j.bse.2012.04.015.

395. Sabine von Mering JWK. Phylogeny, systematics, and recircumscription of

Juncaginaceae -a cosmopolitan wetland family. *Diversity, Phylogeny, and Evolution in the Monocotyledons, Aarhus University Press* 2010:22-79.

396. Simpson BB. Krameriaceae. *Flora Neotropica, New York Botanical Garden Press* 1989; **49**:1-108.

397. Baumgartner L, Sosa S, Atanasov AG *et al.* Lignan derivatives from *Krameria lappacea* roots inhibit acute inflammation in vivo and pro-inflammatory mediators in vitro. *J Nat Prod* 2011; **74**(8):1779-86. doi: 10.1021/np200343t.

398. Daniel J. Crawford TFS, Mario Silva O. Leaf Flavonoid Chemistry and the Relationships of the Lactoridaceae. *Plant Systematics and Evolution* 1986; **153**:133-139.

399. Zhiqin Guo ZL, Xiaohong Wang *et al.* Elsholtzia: phytochemistry and biological activities. *Chemistry Central Journal* 2012; **6**:1-8.

400. Tundis R, Peruzzi L, Menichini F. Phytochemical and biological studies of *Stachys* species in relation to chemotaxonomy: a review. *Phytochemistry* 2014; **102**:7-39. doi: 10.1016/j.phytochem.2014.01.023.

401. Zielinska S, Matkowski A. Phytochemistry and bioactivity of aromatic and medicinal plants from the genus *Agastache* (Lamiaceae). *Phytochem Rev* 2014; **13**:391-416. doi: 10.1007/s11101-014-9349-1.

402. Cioffi G, Bader A, Malafronte A *et al.* Secondary metabolites from the aerial parts of *Salvia palaestina* Bentham. *Phytochemistry* 2008; **69**(4):1005-12. doi: 10.1016/j.phytochem.2007.11.002.

403. Huang X. Chemotaxonomy of angiosperm: Lamiaceae. *J Int Pharm Res* 1984; **3**:129-137.

404. Xue X, Xiao Y, Gong L *et al.* Comparative 28-day repeated oral toxicity of Longdan Xieganwan, *Akebia trifoliate* (Thunb.) koidz., *Akebia quinata* (Thunb.) Decne. and *Caulis aristolochiae* manshuriensis in mice. *J Ethnopharmacol* 2008; **119**(1):87-93. doi: 10.1016/j.jep.2008.05.037.

405. Ikuta A. Saponins and Triterpenes from Callus Tissues of *Akebia trifoliata* and Comparison with the Constituents of Other Lardizabalaceous Callus Tissues. *Journal of Natural Prducts* 1995; **58**(9):1378-1383.

406. Jiang D, Shi S-P, Cao J-J *et al.* Triterpene saponins from the fruits of *Akebia quinata*. *Biochem Syst Ecol* 2008; **36**(2):138-141. doi: 10.1016/j.bse.2007.06.004.

407. Gao H, Wang Z. Triterpenoid saponins and phenylethanoid glycosides from stem of *Akebia trifoliata* var. *australis*. *Phytochemistry* 2006; **67**(24):2697-705. doi: 10.1016/j.phytochem.2006.09.003.

408. Zheng Qing YC-R. Chemotaxonomic study on the family of Lardizabalaceae. *Chinese Bulletin of Botany* 2001; **18**(3):332-339.

409. Batista AL, Yoshida NC, Garcez FR *et al.* Chemical constituents from *Nectandra cuspidata* Nees – Lauraceae. *Biochem Syst Ecol* 2015; **61**:229-231. doi: 10.1016/j.bse.2015.06.007.

410. Coy Barrera ED, Cuca Suárez LE. Chemical constituents from *Pleurothyrium cinereum* (van der Werff) (Lauraceae) from Colombia. *Biochem Syst Ecol* 2008; **36**(8):674-677. doi: 10.1016/j.bse.2008.05.007.

411. Mi-Ran Kim H-JJ, Byung-Sun Min *et al.* Constituents from the stems of *Actinodaphne lancifolia* (Lauraceae). *Phytochemistry* 2002; **59**:861-865.

412. Liu R, Zhang H-C, Zhou F *et al.* Flavonoids and alkaloids from the leaves of *Litsea fruticosa*. *Biochem Syst Ecol* 2013; **50**:293-295. doi: 10.1016/j.bse.2013.04.008.

413. Murai Y, Kokubugata G, Yokota M *et al.* Flavonoids and anthocyanins from six *Cassytha taxa* (Lauraceae) as taxonomic markers. *Biochem Syst Ecol* 2008; **36**(9):745-748. doi: 10.1016/j.bse.2008.06.007.

414. Subhash C. Joshi RCP, Dinesh S. Bisht, Chandra S. Mathela. Terpenoid diversity in the leaf essential oils of himalayan Lauraceae Species. *Chemistry & Biodiversity* 2009; **6**:1363-1373.

415. McRae JM, Yang Q, Crawford RJ *et al.* Acylated flavonoid tetraglycoside from *Planchonia careya* leaves. *Phytochemistry Letters* 2008; **1**(2):99-102. doi: 10.1016/j.phytol.2008.04.003.

416. McRae JM, Yang Q, Crawford RJ *et al.* Antibacterial compounds from *Planchonia careya* leaf extracts. *J Ethnopharmacol* 2008; **116**(3):554-60. doi: 10.1016/j.jep.2008.01.007.

417. Lin CY, Chen YH, Chang TC *et al.* Characteristic aroma-active compounds of floral scent in situ from *Barringtonia racemosa* and their dynamic emission rates. *J Agric Food Chem* 2013; **61**(51):12531-8. doi: 10.1021/jf404505p.

418. Jocélia P. C. Oliveira ÉLFF, Mariana H. Chaves *et al.* Chemical constituents of *Lecythis pisonis* and cytotoxic activity. *Revista Brasileira de Farmacognosia Brazilian Journal of Pharmacognosy* 2012; **22**(5):1140-1144. doi: 10.1590/s0102695x2012005000053.

419. Grevenstuk T, van der Hooft JJJ, Vervoort J *et al.* Iridoid and caffeoyl phenylethanoid glycosides of the endangered carnivorous plant *Pinguicula lusitanica* L. (Lentibulariaceae). *Biochem Syst Ecol* 2009; **37**(4):285-289. doi: 10.1016/j.bse.2009.05.003.

420. Laurence Dinan PW, Tamara Savchenko. Phytoecdysteroids in seeds of *Lloydia serotina* (Liliaceae). *Biochem Syst Ecol* 2001; **29**:923-928.

421. Su Z. Chemotaxonomy of Liliaceae. *Foreign Medical Sciences (Section of Pharmacy)* 1979; **4**(193-199).

422. Beejmohun V, Fliniaux O, Hano C *et al.* Coniferin dimerisation in lignan biosynthesis in flax cells. *Phytochemistry* 2007; **68**(22-24):2744-52. doi: 10.1016/j.phytochem.2007.09.016.

423. Attoumbré J, Mahamane Laoualy AB, Bienaimé C *et al.* Investigation of lignan accumulation in developing *Linum usitatissimum* seeds by immunolocalization and HPLC. *Phytochemistry Letters* 2011; **4**(2):194-198. doi: 10.1016/j.phytol.2011.03.004.

424. Schmidt TJ, Hemmati S, Klaes M *et al.* Lignans in flowering aerial parts of *Linum* species--chemodiversity in the light of systematics and phylogeny. *Phytochemistry* 2010; **71**(14-15):1714-28. doi: 10.1016/j.phytochem.2010.06.015.

425. Schmidt TJ, Klaes M, Sendker J. Lignans in seeds of *Linum* species. *Phytochemistry* 2012; **82**:89-99. doi: 10.1016/j.phytochem.2012.07.004.

426. Belma Konuklugil OB. Phenylpropanoid Glycosides from *Linum olympicum* (Linaceae). *Turkish Journal of Chemistry* 2004; **28**:741-744.

427. Hemmati S, Schmidt TJ, Fuss E. (+)-Pinoresinol/(-)-lariciresinol reductase from *Linum perenne* Himmelszelt involved in the biosynthesis of justicidin B. *FEBS Lett* 2007; **581**(4):603-10. doi: 10.1016/j.febslet.2007.01.018.

428. von Heimendahl CB, Schafer KM, Eklund P *et al.* Pinoresinol-lariciresinol reductases with different stereospecificity from *Linum album* and *Linum usitatissimum*. *Phytochemistry* 2005; **66**(11):1254-63. doi: 10.1016/j.phytochem.2005.04.026.

429. A.A. Muller JKK, K.G. Dietl *et al.* Iridoid glucosides-chemotaxonomic markers in Loasoideae. *Phytochemistry* 1999; **52**:67-78.

430. Khera S, Woldemichael GM, Singh MP *et al.* A novel antibacterial iridoid and triterpene from *Caiophora coronata*. *J Nat Prod* 2003; **66**(12):1628-31. doi: 10.1021/np030314a.

431. Maximilian Weigend JK, Andereas A. Muller. Phytochemistry and the systematics and ecology of Loasaceae and Gronoviaceae (Loasales). *Am J Bot*2000; **87**(8):1202-1210.

432. Yaron Sitrit SL, Racheli Ninio *et al.* Characterization of Monkey Orange (*Strychnos spinosa* Lam.), a Potential New Crop for Arid Regions. *J Agr Food Chem*2003; **51**:6256-6260.

433. M. Daniel SDS. Chemotaxonomy of Loganiaceae. *Current Science* 1979; **48**(9):383-385.

434. Nicoletti M. Flavonoids and Alkaloids from *Strychnos pseudoquina*. *J Nat Prod* 1984; **47**(6):953-957.

435. Gong Zhunan WY, Liang Qiaoli *et al.* A chemotaxonomic study of 27 species of the Loranthaceae plan t from China. *Guihaia* 2004; **24**(6):493-396.

436. Deborah Yara A. C. Santos MLFS, Antonio Salatino. Foliar flavonoids of *Lafoensia* (Lythraceae). *Biochem Syst Ecol* 2000; **28**:487-488.

437. Perez-Castorena AL, Maldonado E. Triterpenes and flavonoid glycosides from *Cuphea wrightii*. *Biochem Syst Ecol* 2003; **31**(3):331-334. doi: 10.1016/s0305-1978(02)00159-x.

438. Mirosław Furmanowa JJ. Alkaloids as taxonomic markers in some species of *Magnolia* L. and Liriodendron L. *Acta Societatis Botanicorum Poloniae* 2014; **49**(4):527-535.

439. Porter EA, Kite GC, Veitch NC *et al.* Phenylethanoid glycosides in tepals of *Magnolia salicifolia* and their occurrence in flowers of Magnoliaceae. *Phytochemistry* 2015; **117**:185-93. doi: 10.1016/j.phytochem.2015.02.025.

440. Li Shi-Sheng TN-H, Zhou Jun *et al.* Phytochemical and chemotaxonomic studies on *Liriodendron chinense* and *Paramichelia baillonii* (Magnoliaceae). *Acta Botanica Yunnanica* 2001; **23**(1):115-120.

441. Queiroz MM, Queiroz EF, Zeraik ML *et al.* Chemical composition of the bark of *Tetrapterys mucronata* and identification of acetylcholinesterase inhibitory constituents. *J Nat Prod* 2014; **77**(3):650-6. doi: 10.1021/np401003p.

442. Shugeng Cao RCG, James S. Miller *et al.* Cytotoxic Triterpenoids from *Acridocarpus vivy* from the Madagascar Rain Forest (Malpighiaceae). *J. Nat. Prod.* 2004; **67**:986-989.

443. Motta LB, Furlan CM, Salatino A *et al.* Flavonoids and the taxonomy of *Camarea* (Malpighiaceae). *Biochem Syst Ecol* 2009; **37**(3):201-205. doi: 10.1016/j.bse.2009.03.005.

444. Motta LB, Salatino A, Salatino MLF. Foliar cuticular alkanes of *Camarea* (Malpighiaceae) and their taxonomic significance. *Biochem Syst Ecol* 2009; **37**(1):35-39. doi: 10.1016/j.bse.2008.11.016.

445. de Sousa LR, Ramalho SD, Burger MC *et al.* Isolation of arginase inhibitors from the bioactivity-guided fractionation of *Byrsonima coccolobifolia* leaves and stems. *J Nat Prod* 2014; **77**(2):392-6. doi: 10.1021/np400717m.

446. Oliveira da Silva AC, Morais de Oliveira AF, Alves Cursino dos Santos DY *et al.* An approach to chemotaxonomy to the fatty acid content of some Malvaceae species. *Biochem Syst Ecol* 2010; **38**(5):1035-1038. doi: 10.1016/j.bse.2010.10.006.

447. Rizk RM, Soliman MI. Biochemical and molecular genetic characterization of some species of family Malvaceae, Egypt. *Egyptian Journal of Basic and Applied Sciences* 2014; **1**(3-4):167-176. doi: 10.1016/j.ejbas.2014.06.002.

448. Lai X-Y, Zhao Y-Y, Liang H. A flavonoid glucuronide from *Abelmoschus manihot* (L.) Medik. *Biochem Syst Ecol* 2007; **35**(12):891-893. doi: 10.1016/j.bse.2007.04.007.

449. Gossan DP, Alabdul Magid A, Kouassi-Yao PA *et al.* Glycosidase inhibitors from the roots of *Glyphaea brevis*. *Phytochemistry* 2015; **109**:76-83. doi: 10.1016/j.phytochem.2014.10.029.

450. Dinan L, Bourne P, Whiting P. Phytoecdysteroid profiles in seeds of *Sida* spp. (Malvaceae). *Phytochem Anal* 2001; **12**(2):110-9. doi: 10.1002/pca.566.

451. Erwin, Noor A, Soekamto NH *et al.* Waltherione C and cleomiscosin from *Melochia umbellata* var. *degrabrata* K. (Malvaceae), biosynthetic and chemotaxonomic significance. *Biochem Syst Ecol* 2014; **55**:358-361. doi: 10.1016/j.bse.2014.03.020.

452. Abdullah Y, Schneider B, Petersen M. Occurrence of rosmarinic acid, chlorogenic acid and rutin in Marantaceae species. *Phytochemistry Letters* 2008; **1**(4):199-203. doi: 10.1016/j.phytol.2008.09.010.

453. Carballo-Arce AF, Ta CAK, Rocha MEdN *et al.* Antimicrobial activities of Marcgraviaceae species and isolation of a naphthoquinone from *Marcgravia nervosa* (Marcgraviaceae). *Botany* 2015; **93**(7):413-424. doi: 10.1139/cjb-2015-0038.

454. Wang Y, Gao W, Li X *et al.* Chemotaxonomic study of the genus *Paris* based on steroidal saponins. *Biochem Syst Ecol* 2013; **48**:163-173. doi: 10.1016/j.bse.2012.12.011.

455. Irwin RE, Cook D, Richardson LL *et al.* Secondary compounds in floral rewards of toxic rangeland plants: impacts on pollinators. *J Agric Food Chem* 2014; **62**(30):7335-44. doi: 10.1021/jf500521w.

456. Challinor VL, Stuthe JM, Parsons PG *et al.* Structure and bioactivity of steroidal saponins isolated from the roots of *Chamaelirium luteum* (false unicorn). *J Nat Prod* 2012; **75**(8):1469-79. doi: 10.1021/np300393y.

457. Michelangeli FA, Rodriguez E. Absence of cyanogenic glycosides in the tribe Miconieae (Melastomataceae). *Biochem Syst Ecol* 2005; **33**(4):335-339. doi: 10.1016/j.bse.2004.11.008.

458. Marcia C. Bomfim-Patrıcioa AS, Angela B. Martins. Flavonoids of *Lavoisiera*, *Microlicia* and *Trembleya* (Melastomataceae) and their taxonomic meaning. *Biochem Syst Ecol* 2001; **29**:711-726.

459. Sirat HM, Rezali MF, Ujang Z. Isolation and identification of radical scavenging and tyrosinase inhibition of polyphenols from *Tibouchina semidecandra* L. *J Agric Food Chem* 2010; **58**(19):10404-9. doi: 10.1021/jf102231h.

460. Calderón AI, Terreaux C, Gupta MP *et al.* Occurrence of taxiphyllin and 3, 3′-di-O-methylellagic acid 4′-β-D-glucoside in *Henriettella fascicularis*. *Biochem Syst Ecol* 2003; **31**(7):789-791. doi: 10.1016/s0305-1978(03)00019-x.

461. Serna DM, Martinez JH. Phenolics and polyphenolics from Melastomataceae species. *Molecules* 2015; **20**(10):17818-47. doi: 10.3390/molecules201017818.

462. Wilfred R. Chan VS, Kathleen A. Medfor. Triterpenes from *Miconia stenostachya*. *J Nat Prod* 1992; **55**(7):963-966.

463. Rodrigues J, Rinaldo D, dos Santos LC *et al.* An unusual C6-C6" linked flavonoid from *Miconia cabucu* (Melastomataceae). *Phytochemistry* 2007; **68**(13):1781-4. doi: 10.1016/j.phytochem.2007.04.020.

464. Linder HP. Melianthaceae. *Flowering Plants - Eudicots, Springer Berlin Heidelberg* 2007; **9**:250-259.

465. de Wet H, van Heerden FR, van Wyk BE. Alkaloidal variation in *Cissampelos capensis* (Menispermaceae). *Molecules* 2011; **16**(4):3001-9. doi: 10.3390/molecules16043001.

466. De Wet H, van Heerden FR, van Wyk B-E. Alkaloids of *Antizoma angustifolia* (Menispermaceae). *Biochem Syst Ecol* 2004; **32**(12):1145-1152. doi: 10.1016/j.bse.2004.04.003.

467. de Wet H, van Heerden FR, van Wyk B-E. Alkaloids of *Antizoma miersiana* (Menispermaceae). *Biochem Syst Ecol* 2005; **33**(8):799-807. doi: 10.1016/j.bse.2004.12.014.

468. Blanchfield JT, Sands DPA, Kennard CHL *et al.* Characterisation of alkaloids from some Australian *Stephania* (Menispermaceae) species. *Phytochemistry* 2003; **63**(6):711-720. doi: 10.1016/s0031-9422(03)00240-1.

469. Semwal DK, Badoni R, Semwal R *et al.* The genus *Stephania* (Menispermaceae): chemical and pharmacological perspectives. *J Ethnopharmacol* 2010; **132**(2):369-83. doi: 10.1016/j.jep.2010.08.047.

470. Bruce A. Bohm KWNaRO. Flavonoids of the Menyanthaceae: Intra-and Interfamilial Relationships. *Am J Bot*1986; **73**(2):204-213.

471. Biswas T, Gupta M, Achari B *et al.* Hopane-type saponins from *Glinus lotoides* Linn. *Phytochemistry* 2005; **66**(6):621-6. doi: 10.1016/j.phytochem.2005.02.012.

472. Arafa I Hameda NAE-E. Triterpene saponins from *Glinus lotoides* var. *dictamnoides*. *Phytochemistry* 1999; **50**:447-480.

473. Gilda G. Leitao NKS, Simone S.V. Soares *et al.* Chemistry and pharmacology of Monimiaceae: a special focus on *Siparuna* and *Mollinedia*. *Journal of Ethnopharmacology* 1999; **65**:87-102.

474. Royer M, Herbette G, Eparvier V *et al.* Secondary metabolites of *Bagassa guianensis* Aubl. wood: a study of the chemotaxonomy of the Moraceae family. *Phytochemistry* 2010; **71**(14-15):1708-13. doi: 10.1016/j.phytochem.2010.06.020.

475. Felipe Otalvaro HG, Dirk Holscher *et al.* Dimeric phenylphenalenones from *Musa acuminata* and various Haemodoraceae species. Crystal structure of anigorootin. *Phytochemistry* 2002; **60**:61-66.

476. Javier G. Luis FE, Winston Quifiones *et al.* Irenolone and emenolone: two new types of phytoalexin from *Musa paradisiaca*. *J. Org. Chem* 1993; **58**(16):4306-4308.

477. Gutiérrez Jdrjj-Bmcmpa. Phenylphenalenone type compounds from the leaf fibers of abaca (*Musa textilis*). *J Agr Food Chem*2006; **54**(23):8744-8748.

478. Corke Jbycmsgwh. Anthocyanins, Flavonols, and Free Radical Scavenging Activity of Chinese Bayberry (*Myrica rubra*) Extracts and Their Color Properties and Stability. *J Agr Food Chem*2005; **53**(6):2327-2332.

479. Yang H, Ye X, Liu D *et al.* Characterization of unusual proanthocyanidins in leaves of bayberry (*Myrica rubra* Sieb. et Zucc.). *J Agric Food Chem* 2011; **59**(5):1622-9. doi: 10.1021/jf103918v.

480. Fang J, Paetz C, Schneider B. C-methylated flavanones and dihydrochalcones from *Myrica gale* seeds. *Biochem Syst Ecol* 2011; **39**(1):68-70. doi: 10.1016/j.bse.2011.01.009.

481. Yoshimura M, Yamakami S, Amakura Y *et al.* Diarylheptanoid sulfates and related compounds from *Myrica rubra* bark. *J Nat Prod* 2012; **75**(10):1798-802. doi: 10.1021/np300212c.

482. Wang M, Lincoln DE. Effects of light intensity and artificial wounding on monoterpene production in *Myrica cerifera* from two different ecological habitats. *Canadian Journal of Botany* 2004; **82**(10):1501-1508. doi: 10.1139/b04-107.

483. Spinola V, Llorent-Martinez EJ, Gouveia S *et al. Myrica faya*: a new source of antioxidant phytochemicals. *J Agric Food Chem* 2014; **62**(40):9722-35. doi: 10.1021/jf503540s.

484. Tene M, Tane P, Connolly JD. Triterpenoids and diarylheptanoids from *Myrica arborea*. *Biochem Syst Ecol* 2008; **36**(11):872-874. doi: 10.1016/j.bse.2008.06.008.

485. Yoshida Nldsmkm. Butanolides as a common feature of *Iryanthera lancifolia* and *Virola surinamensis*. *Phytochemistry* 1998; **49**(5):1405-141-.

486. Van Cuong Pham AJ, Thierry Se ´venet and Bernard Bodo. Cytotoxic Acylphenols from *Myristica maingayi*. *Tetrahedron* 2000; **56**:1707-1713.

487. Valderrama JCMn. Distribution of flavonoids in the Myristicaceae. *Phytochemistry* 2000; **55**:505-511.

488. Sergio M. Nunomura MY. Lignans and benzoic acid derivatives from pericarps of *Virola multinervia* (Myristicaceae). *Biochem Syst Ecol* 2000; **30**:985-987.

489. Yoshida Ebmsm. Lignoids, flavanoids and polyketides of *Virola surinamensis*. *Phytochemistry* 1997; **46**(4):745-749.

490. Gleadow RM, Vecchies AC, Woodrow IE. Cyanogenic *Eucalyptus nobilis* is polymorphic for both prunasin and specific β-glucosidases. *Phytochemistry* 2003; **63**(6):699-704. doi: 10.1016/s0031-9422(03)00245-0.

491. Gleadow RM, Haburjak J, Dunn JE *et al.* Frequency and distribution of cyanogenic glycosides in *Eucalyptus* L'Herit. *Phytochemistry* 2008; **69**(9):1870-4. doi: 10.1016/j.phytochem.2008.03.018.

492. Lapčík O, Klejdus B, Kokoška L *et al.* Identification of isoflavones in *Acca sellowiana* and two *Psidium* species (Myrtaceae). *Biochem Syst Ecol* 2005; **33**(10):983-992. doi: 10.1016/j.bse.2005.03.007.

493. Margareth B. C. Gallo FCdS, Paulo C. Vieira *et al.* New natural products from *Siphoneugena densiflora* Berg (Myrtaceae) and their chemotaxonomic significance. *J. Braz. Chem. Soc* 2006; **17**(2):279-288.

494. Mohammed AMA, Coombes PH, Crouch NR *et al.* Non-volatile isolates of two *Heteropyxis* species: A first chemotaxonomic assessment of subfamily Psiloxyloideae (Myrtaceae). *Biochem Syst Ecol* 2009; **37**(3):241-243. doi: 10.1016/j.bse.2009.03.006.

495. Tian L-W, Xu M, Wang D *et al.* Phenolic constituents from the leaves of *Syzygium forrestii* Merr. and Perry. *Biochem Syst Ecol* 2011; **39**(2):156-158. doi: 10.1016/j.bse.2011.01.014.

496. Brophy JJ, Craig DC, Goldsack RJ *et al.* Triumphalone, a diketone from the volatile oil of the leaves of *Melaleuca triumphalis*, and its spontaneous conversion into isotriumphalone. *Phytochemistry* 2006; **67**(18):2085-9. doi: 10.1016/j.phytochem.2006.06.003.

497. Hao Q, Li R, Yao Y *et al.* C-glycosylflavone from *Aletris spicata*. *Biochem Syst Ecol* 2012; **45**:191-193. doi: 10.1016/j.bse.2012.07.035.

498. Li LZ, Wang MH, Sun JB *et al.* Flavonoids and other constituents from *Aletris spicata* and their chemotaxonomic significance. *Nat Prod Res* 2014; **28**(15):1214-7. doi: 10.1080/14786419.2014.921918.

499. Sakushima Snhthkska. Alkaloids from embryo of the seed of *Nelumbo nucifera*. *J Nat Prod* 2004; **49**(3):547-548.

500. Mithofer A. Carnivorous pitcher plants: insights in an old topic. *Phytochemistry* 2011; **72**(13):1678-82. doi: 10.1016/j.phytochem.2010.11.024.

501. Marzouk MM, Hussein SR, Ibrahim LF *et al.* Flavonoids from *Neurada procumbens* L. (Neuradaceae) in Egypt. *Biochem Syst Ecol* 2014; **57**:67-68. doi: 10.1016/j.bse.2014.07.001.

502. Wang KB, Di YT, Bao Y *et al.* Peganumine A, a beta-carboline dimer with a new octacyclic scaffold from *Peganum harmala*. *Org Lett* 2014; **16**(15):4028-31. doi: 10.1021/ol501856v.

503. Salame R, Gravel E, Poupon E. Questions about the structures of nitraraine and nitraraidine. *Tetrahedron Letters* 2011; **52**(48):6453-6456. doi: 10.1016/j.tetlet.2011.09.101.

504. Thoison O, Sevenet T, Niemeyer HM *et al.* Insect antifeedant compounds from *Nothofagus dombeyi* and *N. pumilio*. *Phytochemistry* 2004; **65**(14):2173-6. doi: 10.1016/j.phytochem.2004.04.002.

505. Wollenweber E, Stevens JF, Dörr M *et al.* Taxonomic significance of flavonoid variation in temperate species of *Nothofagus*. *Phytochemistry* 2003; **62**(7):1125-1131. doi: 10.1016/s0031-9422(02)00666-0.

506. G. Nageshwar MR. A note on the chemosystematics of *Boerhavia* and *Bouginvillea* (Nyctaginaceae). *Feddes Repertorium* 1994; **105**:45-47.

507. Shu-Wei Yang RU, James McAlpine *et al.* Three new phenolic compounds from a manipulated plant cell culture, *Mirabilis jalapa*. *J. Nat. Prod.* 2001; **64**: 313-317.

508. Messana FFI. Two new isoflavonoids from *Boerhaavia coccinea*. *J. Nat. Prod.* 1991; **54**(2):597-598.

509. Andersen TFØM. Acylated anthocyanins from leaves of the water lily, *Nymphaéa marliacea*. *Phytochemistry* 1997; **46**(2):353-357.

510. Andersen Tfålbkøm. Flavonoids from blue flowers of *Nymphaèa caerulea*. *Phytochemistry* 1999; **51**(8):1133-1137.

511. Elo Manga SS, Tih AE, Ghogomu RT *et al.* Biflavonoid constituents of *Campylospermum mannii*. *Biochem Syst Ecol* 2009; **37**(4):402-404. doi: 10.1016/j.bse.2009.04.002.

512. Fidelis QC, Castro RN, Guilhon GM *et al.* Flavonoids and other compounds from *Ouratea ferruginea* (Ochnaceae) as anticancer and chemopreventive agents. *Molecules* 2012; **17**(7):7989-8000. doi: 10.3390/molecules17077989.

513. Pegnyemb DE, Tih RG, Sondengam BL *et al.* Flavonoids from leaves of *Ochna afzelii*. *Biochem Syst Ecol* 2003; **31**(2):219-221. doi: 10.1016/s0305-1978(02)00089-3.

514. Zintchem AAà, Atchadé AdT, Tih RG *et al.* Flavonoids from *Ouratea staudtii* Van Tiegh. (ex Keay) (Ochnaceae). *Biochem Syst Ecol* 2007; **35**(4):255-256. doi: 10.1016/j.bse.2006.10.018.

515. Pegnyemb DE, Tih RG, Sondengam BL *et al.* Flavonoids of *Ochna afzelii*. *Phytochemistry* 2003; **64**(2):661-665. doi: 10.1016/s0031-9422(03)00267-x.

516. Babajide OJ, Babajide OO, Daramola AO *et al.* Flavonols and an oxychromonol from *Piliostigma reticulatum*. *Phytochemistry* 2008; **69**(11):2245-50. doi: 10.1016/j.phytochem.2008.05.003.

517. Likhitwitayawuid K, Kaewamatawong R, Ruangrungsi N. Mono- and biflavonoids of *Ochna integerrima*. *Biochem Syst Ecol* 2005; **33**(5):527-536. doi: 10.1016/j.bse.2004.10.014.

518. Abouem a Zintchem A, Bikobo DN, de Theodore Atchade A *et al.* Nitrile glucosides and serotobenine from *Campylospermum glaucum* and *Ouratea turnarea*. *Phytochemistry* 2008; **69**(11):2209-13. doi: 10.1016/j.phytochem.2008.04.013.

519. Fidelis QC, Ribeiro TAN, Araújo MF *et al. Ouratea* genus: chemical and pharmacological aspects. *Revista Brasileira de Farmacognosia* 2014; **24**(1):1-19. doi: 10.1590/0102-695x20142413361.

520. Ndongo JT, Shaaban M, Mbing JN *et al.* Phenolic dimers and an indole alkaloid from *Campylospermum flavum* (Ochnaceae). *Phytochemistry* 2010; **71**(16):1872-8. doi: 10.1016/j.phytochem.2010.08.006.

521. Mbing JN, Pegnyemb DE, Tih RG *et al.* Two biflavonoids from *Ouratea flava* stem bark. *Phytochemistry* 2003; **63**(4):427-431. doi: 10.1016/s0031-9422(03)00161-4.

522. Ngo Mbing J, Enguehard-Gueiffier C, Atchade Ade T *et al.* Two biflavonoids from *Ouratea nigroviolacea*. *Phytochemistry* 2006; **67**(24):2666-70. doi: 10.1016/j.phytochem.2006.07.027.

523. Gntbdbe. Structure and dynamic of three indole alkaloids from the *Campylospermum* genus (Ochnaceae). *Helvetica Chimica Acta* 2013; **96**(7):1298-1304.

524. El-Seedi HR, Larsson S, Backlund A. Chemosystematic value of cyclopeptide alkaloids from *Heisteria nitida* (Olacaceae). *Biochem Syst Ecol* 2005; **33**(8):831-839. doi: 10.1016/j.bse.2004.12.023.

525. Wanxia Tang HH, Kenichi Harada, Miwa Kubo *et al.* Clerodane diterpenoids with NGF-potentiating activity from *Ptychopetalum olacoides*. *J. Nat. Prod.* 2008; **71**:1760-1763.

526. Jerz G, Waibel R, Achenbach H. Cyclohexanoid protoflavanones from the stem-bark and roots of *Ongokea gore*. *Phytochemistry* 2005; **66**(14):1698-706. doi: 10.1016/j.phytochem.2005.04.031.

527. Ping NHS. Distribution and taxonomic significance of flavonoids in the Olacaceae and Icacinaceae. *Biochemical Systematics & Ecology* 1997; **25**(3):263-265.

528. Ma J, Pawar RS, Grundel E *et al.* Sesquiterpenoid tropolone glycosides from *Liriosma ovata*. *J Nat Prod* 2015; **78**(2):315-9. doi: 10.1021/np5006696.

529. Kostova I, Iossifova T. Chemical components of *Fraxinus* species. *Fitoterapia* 2007; **78**(2):85-106. doi: 10.1016/j.fitote.2006.08.002.

530. Søren Rosendal Jensena HF, Eva Wallander. Chemotaxonomy of the Oleaceae: iridoids as taxonomic markers. *Phytochemistry* 2002; **60**:213–231.

531. Egan P, Middleton P, Shoeb M *et al.* GI 5, a dimer of oleoside, from *Fraxinus* *excelsior* (Oleaceae). *Biochem Syst Ecol* 2004; **32**(11):1069-1071. doi: 10.1016/j.bse.2004.04.007.

532. Romani A, Pinelli P, Mulinacci N *et al.* HPLC Analysis of flavonoids and secoiridoids in leaves of *Ligustrum vulgare* L. (Oleaceae). *J Agr Food Chem*2000; **48**(9):4091-4096. doi: 10.1021/jf9913256.

533. Gousiadou C, Kokubun T, Martins J *et al.* Iridoid glucosides in the endemic *Picconia azorica* (Oleaceae). *Phytochemistry* 2015; **115**:171-4. doi: 10.1016/j.phytochem.2015.01.010.

534. Deliu Constantin Acatmm. *Epilobium* Sp. (Willow herb): micropropagation and production of secondary metabolites. *Biotechnology for Medicinal Plants* 2012; **6**:149-170.

535. Yang J, Jiang J, Huang G *et al.* Phenanthrenes from *Eria stricta* Lindl. *Biochem Syst Ecol* 2014; **54**:333-336. doi: 10.1016/j.bse.2014.03.011.

536. Liu L, Li J, Zeng K-W *et al.* Three new phenanthrenes from *Cremastra appendiculata* (D. Don) Makino. *Chinese Chemical Letters* 2013; **24**(8):737-739. doi: 10.1016/j.cclet.2013.03.045.

537. Ju J. The study on chemical constitute of Orchidaceae. *Drugs Clinic* 2000; **3**:95-104.

538. Jiang Y, Tu PF. Analysis of chemical constituents in *Cistanche* species. *J Chromatogr A* 2009; **1216**(11):1970-9. doi: 10.1016/j.chroma.2008.07.031.

539. Liu X-M, Li J, Jiang Y *et al.* Chemical constituents from *Cistanche sinensis* (Orobanchaceae). *Biochem Syst Ecol* 2013; **47**:21-24. doi: 10.1016/j.bse.2012.09.003.

540. Qu Z-y, Zhang Y-w, Yao C-l *et al.* Chemical constituents from *Orobanche cernua* Loefling. *Biochem Syst Ecol* 2015; **60**:199-203. doi: 10.1016/j.bse.2015.04.028.

541. Huang W, Wu S-B, Wang Y-L *et al.* Chemical constituents from *Striga asiatica* and its chemotaxonomic study. *Biochem Syst Ecol* 2013; **48**:100-106. doi: 10.1016/j.bse.2012.10.010.

542. Daňková I, Žemlička M, Švajdlenka E *et al.* The chemotaxonomic significance of phenylethanoid glycosides of *Lathraea squamaria* L. (Orobanchaceae). *Biochem Syst Ecol* 2016; **64**:53-56. doi: 10.1016/j.bse.2015.11.006.

543. Zhang RX, Li MX, Jia ZP. *Rehmannia glutinosa*: review of botany, chemistry and pharmacology. *J Ethnopharmacol* 2008; **117**(2):199-214. doi: 10.1016/j.jep.2008.02.018.

544. Rasmussen LS, Rank C, Jensen SR. Transfer of iridoid glucosides from host plant *Galium verum* to hemiparasitic *Euphrasia stricta*. *Biochem Syst Ecol* 2006; **34**(10):763-765. doi: 10.1016/j.bse.2006.05.006.

545. Andersen Srtfø. Flavone C-Glycosides from leaves of *Oxalis triangularis*. *J Agr Food Chem*2006; **53**(26):10057-10060

546. lwashina T. The structure and distribution of the flavonoids in plants. *Journal of Plant Research* 2000; **113**:287-299.

547. Tsai YC, Yu ML, El-Shazly M *et al.* Alkaloids from *Pandanus amaryllifolius*: isolation and their plausible biosynthetic formation. *J Nat Prod* 2015; **78**(10):2346-54. doi: 10.1021/acs.jnatprod.5b00252.

548. Tan MA, Kitajima M, Kogure N *et al.* New pyrrolidine alkaloids from the roots of *Pandanus amaryllifolius*. *Tetrahedron Letters* 2010; **51**(31):4143-4146. doi: 10.1016/j.tetlet.2010.05.150.

549. Rajeswari J, Kesavan K, Jayakar B. Phytochemical and pharmacological evaluation of prop roots of *Pandanus fascicularis* Lam. *Asian Pacific Journal of Tropical Medicine* 2011; **4**(8):649-653. doi: 10.1016/s1995-7645(11)60165-x.

550. Tan MA, Nonato MG, Kogure N *et al.* Secondary metabolites from *Pandanus simplex*. *Biochem Syst Ecol* 2012; **40**:4-5. doi: 10.1016/j.bse.2011.09.001.

551. Bhattacharjee P, Kshirsagar A, Singhal RS. Supercritical carbon dioxide extraction of 2-acetyl-1-pyrroline from *Pandanus amaryllifolius* Roxb. *Food Chemistry* 2005; **91**(2):255-259. doi: 10.1016/j.foodchem.2004.01.062.

552. Yu J, Xiao P-G. A preliminary study of the chemistry and systematics of Paeoniaceae. *Journal of University of Chinese Academy of Sciences* 1990; **25**(3):172-179.

553. Yu X, Gao X, Zhu Z *et al.* Alkaloids from the tribe Bocconieae (papaveraceae): a chemical and biological review. *Molecules* 2014; **19**(9):13042-60. doi: 10.3390/molecules190913042.

554. Sabine Krist Gshuafbgb. Analysis of volatile compounds and triglycerides of seed oils extracted from different poppy varieties (*Papaver somniferum* L.). *J Agr Food Chem*2005; **53**(21):8310-8316.

555. Meyer A, Imming P. Benzylisoquinoline alkaloids from the Papaveraceae: the heritage of Johannes Gadamer (1867-1928). *J Nat Prod* 2011; **74**(11):2482-7. doi: 10.1021/np2005049.

556. Preininger V. Chemotaxonomy of the Papaveraceae alkaloids. *The Chemistry and Biology of Isoquinoline Alkaloids, Springer-Verlag Berlin Heidelberg* 1985:23-37.

557. Doncheva T, Kostova N, Yordanova G *et al.* Comparison of alkaloid profile from *Glaucium corniculatum* (Papaveraceae) of Algerian and Bulgarian origin. *Biochem Syst Ecol* 2014; **56**:278-280. doi: 10.1016/j.bse.2014.07.007.

558. Ilyas WRM. Flower Pigments. Flavonoids from *Argemone mexicana* Linn. (Papaveraceae). *Journal of Organic Chemistry* 1962; **27**:153-155.

559. Tatsis EC, Bohm H, Schneider B. Occurrence of nudicaulin structural variants in flowers of papaveraceous species. *Phytochemistry* 2013; **92**:105-12. doi: 10.1016/j.phytochem.2013.04.011.

560. Su Z. Chemotaxonomy of Papaveraceae. *J Int Pharm Res* 1981; **2**(1):65-70.

561. Mabry AUT. C-Glycosylflavonoids of *Passiflora serratifolia*. *J. nat. prod* 1980; **43**:162-163.

562. Jaroszewski J. Cyanohydrin glycosides of *Passiflora*: distribution pattern, a saturated cyclopentane derivative from *P. guatemalensis*, and formation of pseudocyanogenic a-hydroxyamidesas isolation artefacts. *Phytochemistry* 2009; **59**(5):501-511.

563. Mabry SMT. Flavonoids of *Passiflora pavonis*. *J Nat Prod* 1981; **44**(5):623-624.

564. Mabry SMT. The flavonoids of *Passiflora sexflora*. *J Nat Prod* 1982; **45**(6):782.

565. Akira Ikuta HT, Yasumasa Morita, and Kouichi Yoshimura. Ursane- and oleanane-type triterpenes from *Ternstroemia gymnanthera* Callus Tissues. *J. Nat. Prod.* 2003; **66**:1051-1054.

566. Era M, Matsuo Y, Shii T *et al.* Diastereomeric ellagitannin isomers from *Penthorum chinense.* *J Nat Prod* 2015; **78**(8):2104-9. doi: 10.1021/acs.jnatprod.5b00439.

567. Iwashina T, Tobe H, Takahashi H *et al.* Flavonoids from achlorophyllous plant, *Petrosavia sakuraii* (Petrosaviaceae). *Biochem Syst Ecol* 2011; **39**(4-6):883-884. doi: 10.1016/j.bse.2011.06.021.

568. Park I, Shin S, Kim C *et al.* Larvicidal activity of lignans identified in *Phryma leptostachya* var. *asiatica* roots against three mosquito species. *J. Agric. Food Chem.* 2005; **53**:969-972.

569. Gaire BP, Subedi L. Phytochemistry, pharmacology and medicinal properties of *Phyllanthus emblica* Linn. *Chin J Integr Med* 2014. doi: 10.1007/s11655-014-1984-2.

570. Liu Y, Young K, Rakotondraibe LH *et al.* Antiproliferative compounds from *Cleistanthus boivinianus* from the Madagascar dry forest. *J Nat Prod* 2015; **78**(7):1543-7. doi: 10.1021/np501020m.

571. Ren Y, Lantvit DD, Deng Y *et al.* Potent cytotoxic arylnaphthalene lignan lactones from *Phyllanthus poilanei*. *J Nat Prod* 2014; **77**(6):1494-504. doi: 10.1021/np5002785.

572. Summon Koul TKR, Andotra CS. Acinospesigenin-A, -B, and -C:  Three New Triterpenoids from *Phytolacca acinosa*. *J. Nat. Prod* 2003; **66**: 1121-1123.

573. Hasrat JA, Pieters L, Claeys M *et al.* Adenosine-1 Active Ligands:  Cirsimarin, a Flavone Glycoside from *Microtea debilis*. *J. Nat. Prod.* 1997(638-641).

574. Wang L, Bai L, Nagasawa T *et al.* Bioactive Triterpene Saponins from the Roots of *Phytolacca americana*. *J. Nat. Prod.* 2008; **71**:35-40.

575. Galarraga Montes E, Amaro-Luis JM. Icosandrin, a novel peltogynoid from the fruits of *Phytolacca icosandra* (Phytolaccaceae). *Nat Prod Res* 2016; **30**(1):89-94. doi: 10.1080/14786419.2015.1038537.

576. Kang S, Woo W. Triterpenes from the Berries of *Phytolacca americana*. *J. nat. prod.* 1980; **43**(4):510-513.

577. Zhen H, Zhang Q. Chemotaxonomy of Phytolaccaceae. *Chin Tradition Med* 1999; **21**(12):650-652.

578. Genderen GNH. Chemical relationship between Pinaceae. *Biochemical Systematics & Ecology* 1980; **8**(3):237-240.

579. Gerson EA, Kelsey RG. Piperidine alkaloids in North American *Pinus* taxa: implications for chemosystematics. *Biochem Syst Ecol* 2004; **32**(1):63-74. doi: 10.1016/s0305-1978(03)00174-1.

580. Semiz G, Heijari J, Isik K *et al.* Variation in needle terpenoids among *Pinus sylvestris* L. (Pinaceae) provenances from Turkey. *Biochem Syst Ecol* 2007; **35**(10):652-661. doi: 10.1016/j.bse.2007.05.013.

581. Santos PRDd, Moreira DdL, Guimaraes EF *et al.* Essential oil analysis of 10 Piperaceae species from the Brazilian Atlantic forest. *Phytochemistry* 2001; **58**:547-551.

582. Parmar V, Jain S, Bisht K *et al.* Phytochemistry of the genus *Piper*. *Phytochemistry* 1997; **46**(4):597-673.

583. Ghosh K, Bhattacharya TK. Chemical Constituents of *Piper betle* Linn. (Piperaceae) Roots. *Molecules* 2005; **10**:798-802.

584. Scott IM, Jensen HR, Philogène BJR *et al.* A review of *Piper* spp. (Piperaceae) phytochemistry, insecticidal activity and mode of action. *Phytochemistry Reviews* 2007; **7**(1):65-75. doi: 10.1007/s11101-006-9058-5.

585. JAY M. Chemotaxonomic researches on vascular plants. XIX. Flavonoid distribution in the Pittosporaceae. *Botanical Journal of the Linnean Society* 1969; **62**:423-429.

586. Seo Y, Berger JM, Hoch J *et al.* A new triterpene saponin from *Pittosporum viridiflorum* from the Madagascar rainforest. *J. Nat. Prod.* 2002; **65**:65-68.

587. Taskova RM, Gotfredsen CH, Jensen SR. Chemotaxonomic markers in *Digitalideae* (Plantaginaceae). *Phytochemistry* 2005; **66**(12):1440-7. doi: 10.1016/j.phytochem.2005.04.020.

588. N. Rønsted, H. Franzyk, P. Mølgaard *et al.* Chemotaxonomy and evolution of *Plantago* L. *Plant Syst. Evol.* 2003; **242**:63-82.

589. Tipirdamaz Gcbybtr. Chemotaxonomy of *Ballota* Species. *Chemistry of Natural Compounds* 2005; **41**(3):299-302.

590. Nina Rønsted, Edith Gobel, Henrik Franzyk *et al.* Chemotaxonomy of *Plantago*. Iridoid glucosides and caffeoyl phenylethanoid glycosides (Plantaginaceae). *Phytochemistry* 2000; **55**:337-348.

591. Taskova RM, Gotfredsen CH, Jensen SR. Chemotaxonomy of Veroniceae and its allies in the Plantaginaceae. *Phytochemistry* 2006; **67**(3):286-301. doi: 10.1016/j.phytochem.2005.11.011.

592. Kaouadji M. Further acylated kaempferol rhamnosides from *Platanus acerifolia* Buds. *J Nat Prod* 1993; **56**(9):1618-1621.

593. Kaouadji M, Ravanel P, Mariotte A. New prenylated flavanones from *Platanus acerifolia* Buds. *J Nat Prod* 1996; **49**(1):153-155.

594. Yue J-M, Xu J. Chemical components from *Ceratostigma willmottianum*. *J. Nat. Prod* 1997; **60**:1031-1033.

595. Bipul R. Acharya, Bhabatarak Bhattacharyya, Chakrabarti G. The natural naphthoquinone plumbagin exhibits antiproliferative activity and disrupts the microtubule network through tubulin binding. *Biochemistry* 2008; **47**:7838–7845.

596. Pensri Whiting, Tamara Savchenko, Satyajit D. Sarker *et al.* Phytoecdysteroids in the genus *Limonium* (Plumbaginaceae). *Biochem Syst Ecol* 1998; **26**:695—698.

597. Murai Y, Fujinami R, Imaichi R *et al.* Flavonoids from riverweeds, *Cladopus japonicus* and *Hydrobryum japonicum* (Podostemaceae). *Biochem Syst Ecol* 2009; **37**(4):538-540. doi: 10.1016/j.bse.2009.07.004.

598. Arisawa M, Kinghorn A, Cordell G *et al.* Ipomopsin, a new biscoumarin from *Ipomopsis aggregata*. *J Nat Prod* 1984; **47**(1):106-112.

599. Arisawa M, Funayama S, Pezzuto J. Potential anticancer agents XXXII. hydroquinone from *Ipomopsis aggregata*. *J Nat Prod* 1984; **47**(2):393-394.

600. Desbène S, Hanquet B, Shoyama Y *et al.* Biologically active triterpene saponins from callus tissue of *Polygala amarella*. *J Nat Prod* 1999; **62**(2): 923-926.

601. Gaoussou T, Anne-Claire M, Tomofumi M *et al.* Presenegenin glycosides from *Securidaca welwitschii*. *Helvetica Chimica Acta* 2010; **93**(11):2237-2244.

602. Tabopda T, Mitaine-Offer A, Miyamoto T *et al.* Acylated triterpene saponins from *Atroxima liberica*. *Helvetica Chimica Acta* 2011; **94**(11):2066-2076.

603. Hokanson G. The lignans of *Polygala polygama* (Polygalaceae): deoxypodophyllotoxin and three new lignan lactones. *J Nat Prod* 2004; **42**(4):378-384.

604. Ma Wenzhe, Wei Xiaoyi, Ling Tiejun *et al.* New phenolics from *Polygala fallax*. *J. Nat. Prod.* 2003; **66**:441-443.

605. Anne-Claire Mitaine-Offer, Tomofumi Miyamoto, Khan IA *et al.* Three new triterpene saponins from two species of *Carpolobia*. *J. Nat. Prod.* 2002; **65**:553-557.

606. Lacaille-Dubois M-A, Mitaine-Offer A-C. Triterpene saponins from Polygalaceae. *Phytochemistry Reviews* 2005; **4**(2-3):139-149. doi: 10.1007/s11101-005-2606-6.

607. Xue-Dong Yang, Li-Zhen Xu, Yang S-L. Xanthones from the stems of *Securidaca inappendiculata*. *Phytochemistry* 2001; **58**:1245-1249.

608. Yao H, Duan J, Ai F *et al.* Chemical constituents from a Chinese fern *Polypodium hastatum* Thunb. *Biochem Syst Ecol* 2012; **44**:275-278. doi: 10.1016/j.bse.2012.06.013.

609. Xiang L, Xing D, Wang W *et al.* Alkaloids from *Portulaca oleracea* L. *Phytochemistry* 2005; **66**(21):2595-601. doi: 10.1016/j.phytochem.2005.08.011.

610. Erkan N. Antioxidant activity and phenolic compounds of fractions from *Portulaca oleracea* L. *Food Chemistry* 2012; **133**(3):775-781. doi: 10.1016/j.foodchem.2012.01.091.

611. Xu X, Yu L, Chen G. Determination of flavonoids in *Portulaca oleracea* L. by capillary electrophoresis with electrochemical detection. *J Pharm Biomed Anal* 2006; **41**(2):493-9. doi: 10.1016/j.jpba.2006.01.013.

612. Ohsaki A, Kasetani Y, Asaka Y *et al.* A diterpenoid from *Portulaca pilosa*. *Phytochemistry* 1995; **40**(1):205-207.

613. Miller JM. Flavonol glycoside variation in diploids of the *Claytonia perfoliata* complex (Portulacaceae). *Systematic Botany* 1981; **6**(1):27-30.

614. Yan J, Sun LR, Zhou ZY *et al.* Homoisoflavonoids from the medicinal plant *Portulaca oleracea*. *Phytochemistry* 2012; **80**:37-41. doi: 10.1016/j.phytochem.2012.05.014.

615. Ayumi Ohsaki, Kozo Shibata, Takashi Kubota *et al.* Phylogenetic and chemotaxonomic significance of diterpenes in some *Portulaca* species (Portulacaceae). *Biochem Syst Ecol* 1999; **27**:289-296.

616. Cocozza C, Parente A, Zaccone C *et al.* Chemical, physical and spectroscopic characterization of *Posidonia oceanica* (L.) Del. residues and their possible recycle. *Biomass and Bioenergy* 2011; **35**(2):799-807. doi: 10.1016/j.biombioe.2010.10.033.

617. Agostini, S, Jm., D, Pergent G. Distribution of phenolic compounds in the seagrass *Posidonia oceanica*. *Phytochemistry* 1998; **48**(4):611-617.

618. Heglmeier A, Zidorn C. Secondary metabolites of *Posidonia oceanica* (Posidoniaceae). *Biochem Syst Ecol* 2010; **38**(5):964-970. doi: 10.1016/j.bse.2010.07.001.

619. Costa J, Desjobert J, Pergent G. Variations in the concentration of phenolic compounds in the seagrass under conditions of competition. *Phytochemistry* 2004; **65**(24):3211-3220. doi: 10.1016/j.phytochem.2004.09.003.

620. Waridel P, Wolfender J-L, Lachavanne J-B *et al.* Ent-Labdane diterpenes from the aquatic plant *Potamogeton pectinatus*. *Phytochemistry* 2003; **64**(7):1309-1317. doi: 10.1016/j.phytochem.2003.08.014.

621. Waridel P, Wolfender JL, Lachavanne JB *et al.* Ent-Labdane glycosides from the aquatic plant *Potamogeton lucens* and analytical evaluation of the lipophilic extract constituents of various *Potamogeton* species. *Phytochemistry* 2004; **65**(7):945-54. doi: 10.1016/j.phytochem.2004.01.018.

622. Cangiano, T, Dellagreca, M, Fiorentino A *et al.* Lactone diterpenes from the aquatic plant *Potamogeton natans*. *Phytochemistry* 2001; **56**(5):496-473.

623. Theunis MH, Foubert K, Pollier J *et al.* Determination of saponins in *Maesa lanceolata* by LC-UV: development and validation. *Phytochemistry* 2007; **68**(22-24):2825-30. doi: 10.1016/j.phytochem.2007.09.019.

624. França H, Corrêa AL, Oliveira AP *et al.* Flavonoids from *Myrsine rubra* M. F. Freitas & Kinoshita (Myrsinaceae). *Biochem Syst Ecol* 2011; **39**(4-6):885-887. doi: 10.1016/j.bse.2011.06.022.

625. Mark S. Butler, Peter L. Katavic, Davis RA *et al.* 10-Hydroxydarlingine, a new tropane alkaloid from the Australian proteaceous plant *Triunia erythrocarpa*. *J. Nat. Prod.* 2000; **63**:688-689.

626. Perry NB, Brennan NJ. Antimicrobial and cytotoxic phenolic glycoside esters from the New Zealand tree *Toronia toru*. *J. Nat. Prod.* 1997; **60**:623-626.

627. Gerhard Lang, Anthony L. J. Cole, John W. Blunt *et al.* Excelsione, a depsidone from an endophytic fungus isolated from the New Zealand endemic tree *Knightia excelsa*. *J. Nat. Prod.* 2007; **70**:310-311.

628. L. Verotta, F. Orsini, F. Pelizzoni *et al.* Polyphenolic glycosides from African Proteaceae. *J. Nat. Prod.* 1999; **62**:1526-1531.

629. Kanchanapoom T, Kamel MS, Picheansoonthon C *et al.* Hydrolyzable tannins and phenylpropanoid from *Rafflesia kerrii* Meijer (Rafflesiaceae). *Journal of Natural Medicines* 2007; **61**(4):478-479. doi: 10.1007/s11418-007-0181-4.

630. Zhang LT, Zhang YW, Takaishi Y *et al.* Antitumor triterpene saponins from *Anemone flaccida*. *Chinese Chemical Letters* 2008; **19**(2):190-192. doi: 10.1016/j.cclet.2007.11.029.

631. Hao D, Gu X, Xiao P *et al.* Chemical and biological research of *Clematis* medicinal resources. *Chinese Science Bulletin* 2012; **58**(10):1120-1129. doi: 10.1007/s11434-012-5628-7.

632. Li, X, Yang, C, Liu Y *et al.* Triterpenoid glycosides from *Anemoclema glaucifolium*. *Phytochemistry* 1995; **39**(5):1175-1179.

633. Li F, Sun C-R, Chen B *et al.* Triterpenoid saponins from *Anemone raddeana*. *Phytochemistry Letters* 2012; **5**(2):258-261. doi: 10.1016/j.phytol.2012.01.007.

634. Berrehal D, Khalfallah A, Kabouche A *et al.* Flavonoid glycosides from *Randonia africana* Coss. (Resedaceae). *Biochem Syst Ecol* 2010; **38**(5):1007-1009. doi: 10.1016/j.bse.2010.09.019.

635. Berrahal D, Kabouche A, Kabouche Z *et al.* Flavonoid glycosides from *Reseda villosa* (Resedaceae). *Biochem Syst Ecol* 2006; **34**(10):777-779. doi: 10.1016/j.bse.2006.06.004.

636. Mitra Noori, Dehshiri MM, Ghorbani M. Investigation of leaf flavonoids of *Reseda* (Tourn.) et L. (Resedaceae) members in markazi province, Iran. *Journal of Medicinal Plants and By-products* 2012; **2**:171-176.

637. Hussein SR, Elkhateeb A, Marzouk MM *et al.* Phytochemical investigation of *Oligomeris linifolia* (Vahl) Macbr. (Resedaceae). *Biochem Syst Ecol* 2013; **49**:73-76. doi: 10.1016/j.bse.2013.03.020.

638. Wu Y, Chen M, Du M-B *et al.* Chemical constituents from the fruit of *Zizyphus jujuba* Mill. var. *spinosa*. *Biochem Syst Ecol* 2014; **57**:6-10. doi: 10.1016/j.bse.2014.07.009.

639. Meng Y-J, Zhang Y-W, Jiang H-Y *et al.* Chemical constituents from the roots of *Zizyphus jujuba* Mill. var. *spinosa.* *Biochem Syst Ecol* 2013; **50**:182-186. doi: 10.1016/j.bse.2013.04.001.

640. Guo S, Duan J-a, Tang Y *et al.* Triterpenoids from the fruits of *Ziziphus jujuba* var. *spinosa*. *Biochem Syst Ecol* 2011; **39**(4-6):880-882. doi: 10.1016/j.bse.2011.06.020.

641. Sudarat Homhual, Nuntavan Bunyapraphatsara, Kondratyuk T *et al.* Bioactive dammarane triterpenes from the mangrove plant *Bruguiera gymnorrhiza*. *J. Nat. Prod.* 2006; **69**:421-424.

642. Ammanamanchi S. R. Anjaneyulu, Vadali Lakshmana Rao, Emil Lobkovsky *et al.* Ceriopsin E, a new epoxy ent-kaurene diterpenoid from *Ceriops decandra*. *J. Nat. Prod.* 2002; **65**:592-594.

643. Manilal A, Merdekios B, Idhayadhulla A *et al.* An in vitro antagonistic efficacy validation of *Rhizophora mucronata*. *Asian Pacific Journal of Tropical Disease* 2015; **5**(1):28-32. doi: 10.1016/s2222-1808(14)60622-8.

644. Nebula M, Harisankar HS, Chandramohanakumar N. Metabolites and bioactivities of Rhizophoraceae mangroves. *Natural Products and Bioprospecting* 2013; **3**(5):207-232. doi: 10.1007/s13659-013-0012-0.

645. Surat Laphookhieo, Chatchanok Karalai, Chanita Ponglimanont *et al.* Pentacyclic triterpenoid esters from the fruits of *Bruguiera cylindrica*. *J. Nat. Prod.* 2004; **67**:886-888.

646. Kato, A, Okada, M, Hashimoto Y. Sulfur-containing alkaloids from *Cassipourea guianensis*. *J Nat Prod* 1985; **48**(2):289-292.

647. Zhang Y, Deng Z, Gao T *et al.* Tagalsins A-H, dolabrane-type diterpenes from the mangrove plant, *Ceriops tagal*. *Phytochemistry* 2005; **66**(12):1465-71. doi: 10.1016/j.phytochem.2005.04.018.

648. Yuki Mikanagi, Norio Saito, Masato Yokoi *et al.* Anthocyanins in flowers of genus *Rosa*, sections *Cinnamomeae* (=Rosa), *Chinenses*, *Gallicanae* and some modern garden roses. *Biochem Syst Ecol* 2000; **28**:887-902.

649. Li J, Deng Y, Yuan C *et al.* Antioxidant and quinone reductase-inducing constituents of black chokeberry (*Aronia melanocarpa*) fruits. *J Agric Food Chem* 2012; **60**(46):11551-9. doi: 10.1021/jf303712e.

650. Kumarasamy Y, Cox PJ, Jaspars M *et al.* Cyanogenic glycosids from *Prunus spinosa* (Rosaceae). *Biochem Syst Ecol* 2003; **31**(9):1063-1065. doi: 10.1016/s0305-1978(03)00063-2.

651. Treutter D, Wang D, Farag MA *et al.* Diversity of phenolic profiles in the fruit skin of *Prunus domestica* plums and related species. *J Agric Food Chem* 2012; **60**(48):12011-9. doi: 10.1021/jf303644f.

652. Murai Y, Iwashina T. Flavonol glucuronides from *Geum calthifolium* var. *nipponicum* and *Sieversia pentapetala* (Rosaceae). *Biochem Syst Ecol* 2010; **38**(5):1081-1082. doi: 10.1016/j.bse.2010.10.012.

653. Tomlinson CTM, Nahar L, Copland A *et al.* Flavonol glycosides from the seeds of *Agrimonia eupatoria* (Rosaceae). *Biochem Syst Ecol* 2003; **31**(4):439-441. doi: 10.1016/s0305-1978(02)00170-9.

654. Sarangowa O, Kanazawa T, Nishizawa M *et al.* Flavonol glycosides in the petal of *Rosa* species as chemotaxonomic markers. *Phytochemistry* 2014; **107**:61-8. doi: 10.1016/j.phytochem.2014.08.013.

655. Mikanagi, Y, Yokoi, M, Ueda, Y *et al.* Flower flavonol and anthocyanin distribution in subgenus *Rosa*. *Biochemical Systematics & Ecology* 1995; **23**(2):183-200.

656. Şöhretoğlu D, Sterner O. Isoflavonoids, flavonoids and flavans from *Potentilla astracanica*. *Biochem Syst Ecol* 2011; **39**(4-6):666-668. doi: 10.1016/j.bse.2011.05.020.

657. Liu Q-B, Huang X-X, Yan X-J *et al.* Neolignans from the seeds of *Prunus tomentosa* (Rosaceae) and their chemotaxonomic interest. *Biochem Syst Ecol* 2014; **55**:236-240. doi: 10.1016/j.bse.2014.03.030.

658. Ogah O, Watkins CS, Ubi BE *et al.* Phenolic compounds in Rosaceae fruit and nut crops. *J Agric Food Chem* 2014; **62**(39):9369-86. doi: 10.1021/jf501574q.

659. Yan G, Li S, Hu J *et al.* Phenolic constituents from the roots of *Rosa laevigata* (Rosaceae). *Biochem Syst Ecol* 2014; **52**:23-26. doi: 10.1016/j.bse.2013.09.006.

660. Şöhretoğlu D, Kırmızıbekmez H. Polyphenols from *Potentilla recta*. *Biochem Syst Ecol* 2011; **39**(2):132-134. doi: 10.1016/j.bse.2011.01.010.

661. Edwards JE, Brown PN, Talent N *et al.* A review of the chemistry of the genus *Crataegus*. *Phytochemistry* 2012; **79**:5-26. doi: 10.1016/j.phytochem.2012.04.006.

662. Tomczyk M. Secondary metabolites from *Potentilla argentea*. *Biochem Syst Ecol* 2006; **34**(10):770-773. doi: 10.1016/j.bse.2006.06.002.

663. Xue P-F, Zhao Y-Y, Wang B *et al.* Secondary metabolites from *Potentilla discolor* Bunge (Rosaceae). *Biochem Syst Ecol* 2006; **34**(11):825-828. doi: 10.1016/j.bse.2006.07.003.

664. Xue P-F, Luo G, Zeng W-z *et al.* Secondary metabolites from *Potentilla multifida* L. (Rosaceae). *Biochem Syst Ecol* 2005; **33**(7):725-728. doi: 10.1016/j.bse.2004.12.012.

665. Tomczyk M. Secondary metabolites from *Potentilla recta* L. and *Drymocallis rupestris* (L.) Soják (syn. *Potentilla rupestris* L.) (Rosaceae). *Biochem Syst Ecol* 2011; **39**(4-6):893-896. doi: 10.1016/j.bse.2011.07.006.

666. Lee J. Sorbitol, *Rubus* fruit, and misconception. *Food Chem* 2015; **166**:616-22. doi: 10.1016/j.foodchem.2014.06.073.

667. Mai K, Reddy M, Radhakrishnaiah M *et al.* Chemosystematics of *Gardenia*. *Proceedings Plant Sciences* 1989; **99**(3):259-264.

668. Mongrand S, Badoc A, Patouille B *et al.* Chemotaxonomy of the Rubiaceae family based on leaf fatty acid composition. *Phytochemistry* 2005; **66**(5):549-59. doi: 10.1016/j.phytochem.2004.12.021.

669. Sirikantaramas S, Sudo H, Asano T *et al.* Transport of camptothecin in hairy roots of *Ophiorrhiza pumila*. *Phytochemistry* 2007; **68**(22-24):2881-6. doi: 10.1016/j.phytochem.2007.08.028.

670. Tian LJ, Yang NY, Chen WQ. Triterpene saponins from *Lysimachia christinae*. *J Asian Nat Prod Res* 2008; **10**(3-4):291-6. doi: 10.1080/10286020701605265.

671. Mukhlesur Rahman M, Anwarul Islam M, Khondkar P *et al.* Alkaloids and lignans from *Zanthoxylum budrunga* (Rutaceae). *Biochem Syst Ecol* 2005; **33**(1):91-96. doi: 10.1016/j.bse.2004.04.016.

672. Sadgrove NJ, Goncalves-Martins M, Jones GL. Chemogeography and antimicrobial activity of essential oils from *Geijera parviflora* and *Geijera salicifolia* (Rutaceae): two traditional Australian medicinal plants. *Phytochemistry* 2014; **104**:60-71. doi: 10.1016/j.phytochem.2014.05.004.

673. Sadgrove NJ, Telford IR, Greatrex BW *et al.* Composition and antimicrobial activity of the essential oils from the *Phebalium squamulosum* species complex (Rutaceae) in New South Wales, Australia. *Phytochemistry* 2014; **97**:38-45. doi: 10.1016/j.phytochem.2013.10.015.

674. Chlouchi A, Girard C, Tillequin F *et al.* Coumarins and furoquinoline alkaloids from *Philotheca deserti* var. *deserti* (Rutaceae). *Biochem Syst Ecol* 2006; **34**(1):71-74. doi: 10.1016/j.bse.2005.07.002.

675. Chlouchi A, Muyard F, Girard C *et al.* Coumarins from the twigs of *Diplolaena mollis* P. G. Wilson (Rutaceae). *Biochem Syst Ecol* 2005; **33**(9):967-969. doi: 10.1016/j.bse.2005.03.002.

676. García-Beltrán O, Areche C, Cassels BK *et al.* Coumarins isolated from *Esenbeckia alata* (Rutaceae). *Biochem Syst Ecol* 2014; **52**:38-40. doi: 10.1016/j.bse.2013.12.011.

677. Braga PAC, Severino VGP, de Freitas SDL *et al.* Dihydrocinnamic acid derivatives from Hortia species and their chemotaxonomic value in the Rutaceae. *Biochem Syst Ecol* 2012; **43**:142-151. doi: 10.1016/j.bse.2012.03.005.

678. Zhou Y, Lv H, Wang W *et al.* Flavonoids and anthraquinones from *Murraya tetramera* C. C. Huang (Rutaceae). *Biochem Syst Ecol* 2014; **57**:78-80. doi: 10.1016/j.bse.2014.07.016.

679. Simonsen HT, Adsersen A, Smitt UW *et al.* Methoxyflavones from *Melicope borbonica* and *M. obscura* (Rutaceae). *Biochem Syst Ecol* 2003; **31**(3):327-330. doi: 10.1016/s0305-1978(02)00158-8.

680. Epifano F, Fiorito S, Genovese S *et al.* Phytochemistry of the genus *Skimmia* (Rutaceae). *Phytochemistry* 2015; **115**:27-43. doi: 10.1016/j.phytochem.2015.02.014.

681. Lukaseder B, Vajrodaya S, Hehenberger T *et al.* Prenylated flavanones and flavanonols as chemical markers in *Glycosmis* species (Rutaceae). *Phytochemistry* 2009; **70**(8):1030-7. doi: 10.1016/j.phytochem.2009.05.007.

682. Chlouchi A, Girard C, Bévalot F *et al.* Taxonomically significant coumarins from three *Philotheca* species (Rutaceae). *Biochem Syst Ecol* 2007; **35**(4):251-254. doi: 10.1016/j.bse.2006.09.011.

683. Yuan Q. Chemotaxonomy of Rutaceae. *J Int Pharm Res* 1986; **5**:257-262.

684. Alabdul Magid A, Morjani H, Harakat D *et al.* Triterpenoid glycosides from the leaves of *Meliosma henryi*. *Phytochemistry* 2015; **109**:49-56. doi: 10.1016/j.phytochem.2014.10.035.

685. Bonnie Rasmussen, Aimee-Justine Nkurunziza, Witt M *et al.* Dovyalicin-type spermidine alkaloids from *Dovyalis* Species. *J. Nat. Prod.* 2006; **69**:1300-1304.

686. Rehill B, Clauss A, Wieczorek L *et al.* Foliar phenolic glycosides from *Populus fremontii*, *Populus angustifolia*, and their hybrids. *Biochem Syst Ecol* 2005; **33**(2):125-131. doi: 10.1016/j.bse.2004.06.004.

687. Zhang XF, Li X, Min BS *et al.* A new diterpenoid from the stem bark of *Populus davidiana*. *Chinese Chemical Letters* 2008; **19**(9):1080-1082. doi: 10.1016/j.cclet.2008.06.005.

688. Kwon D-J, Bae Y-S. Phenolic glucosides from bark of *Populus alba×glandulosa* (Salicaceae). *Biochem Syst Ecol* 2009; **37**(2):130-132. doi: 10.1016/j.bse.2009.01.011.

689. Si C-L, Wu L, Zhu Z-Y. Phenolic glycosides from *Populus davidiana* bark. *Biochem Syst Ecol* 2009; **37**(3):221-224. doi: 10.1016/j.bse.2009.01.007.

690. Boeckler GA, Gershenzon J, Unsicker SB. Phenolic glycosides of the Salicaceae and their role as anti-herbivore defenses. *Phytochemistry* 2011; **72**(13):1497-509. doi: 10.1016/j.phytochem.2011.01.038.

691. Arenas M, Tomás-Barberán F, Núñez, D *et al.* Study of the flavonoids as chematoxonomic markers in *Populus* (Salicaceae) of Spain. preliminary results. *Lagascalia* 1997:813-818.

692. Bennett R, Mellon F, Rosa E *et al.* Profiling Glucosinolates, Flavonoids, Alkaloids, and Other Secondary Metabolites in tissues of *Azima tetracantha* L. (Salvadoraceae). *J Agr Food Chem*2004; **52**:5856-5862.

693. El-Jaber N, Estevez-Braun A, Ravelo AG *et al.* Acetylenic acids from the aerial parts of *Nanodea muscosa*. *J Nat Prod* 2003; **66**(5):722-4. doi: 10.1021/np020513e.

694. Tae Hoon Kim, Hideyuki Ito, Hatano T *et al.* Bisabolane- and Santalane-Type Sesquiterpenoids from *Santalum album* of Indian Origin. *J. Nat. Prod.* 2005; **68**:1805-1808.

695. Iwashina T, López-Sáez JA, Kitajima J. Flavonoids from *Osyris alba*. *Biochem Syst Ecol* 2008; **36**(2):146-147. doi: 10.1016/j.bse.2007.06.008.

696. Do Thi Thu Huong, Marie-Therese Martin, Marc Litaudon *et al.* Pyrrolizidine alkaloids from *Amphorogyne spicata*. *J. Nat. Prod.* 1998; **61**:1444-1446.

697. Kreipl AT, Konig WA. Sesquiterpenes from the east African sandalwood *Osyris tenuifolia*. *Phytochemistry* 2004; **65**(14):2045-9. doi: 10.1016/j.phytochem.2004.05.007.

698. Disadee W, Mahidol C, Sahakitpichan P *et al.* Unprecedented furan-2-carbonyl C-glycosides and phenolic diglycosides from *Scleropyrum pentandrum*. *Phytochemistry* 2012; **74**:115-22. doi: 10.1016/j.phytochem.2011.11.001.

699. Silva FL, Moreno PRH, Braz-Filho R *et al.* Chemical constituents of *Cardiospermum corindum* L. and their distribution in Sapindaceae. *Biochem Syst Ecol* 2014; **57**:137-140. doi: 10.1016/j.bse.2014.07.021.

700. Yuan W, Wang P, Deng G *et al.* Cytotoxic triterpenoid saponins from *Aesculus glabra* Willd. *Phytochemistry* 2012; **75**:67-77. doi: 10.1016/j.phytochem.2011.11.012.

701. Zhang Z, Li S. Cytotoxic triterpenoid saponins from the fruits of *Aesculus pavia* L. *Phytochemistry* 2007; **68**(15):2075-86. doi: 10.1016/j.phytochem.2007.05.020.

702. Ma Q, Xie H, Li S *et al.* Flavonoids from the pericarps of *Litchi chinensis*. *J Agric Food Chem* 2014; **62**(5):1073-8. doi: 10.1021/jf405750p.

703. Voutquenne L, Guinot P, Froissard C *et al.* Haemolytic acylated triterpenoid saponins from *Harpullia austro-caledonica*. *Phytochemistry* 2005; **66**(7):825-35. doi: 10.1016/j.phytochem.2005.02.009.

704. Jankowski WJ, Chojnacki T. The occurrence and characteristics of long chain polyprenols from leaves of Sapindaceae. *Journal of Plant Physiology* 1995; **147**(3-4):289-293. doi: 10.1016/s0176-1617(11)82154-1.

705. Weckerle CS, Stutz MA, Baumann TW. Purine alkaloids in *Paullinia*. *Phytochemistry* 2003; **64**(3):735-742. doi: 10.1016/s0031-9422(03)00372-8.

706. Yuan W, Wang P, Su Z *et al.* Triterpenoid saponins from *Aesculus sylvatica* W. Bartram. *Phytochemistry Letters* 2015; **14**:111-114. doi: 10.1016/j.phytol.2015.09.011.

707. Huang HC, Wu MD, Tsai WJ *et al.* Triterpenoid saponins from the fruits and galls of *Sapindus mukorossi*. *Phytochemistry* 2008; **69**(7):1609-16. doi: 10.1016/j.phytochem.2007.10.033.

708. Zhang Z, Li S, Zhang S *et al.* Triterpenoid saponins from the fruits of *Aesculus pavia*. *Phytochemistry* 2006; **67**(8):784-94. doi: 10.1016/j.phytochem.2006.01.017.

709. Umadevi, I, Daniel M. Chemosystematics of the Sapindaceae. *Feddes Repertorium* 1991; **102**(7-8):607-612.

710. Muhammad A, Guerrero-Analco JA, Martineau LC *et al.* Antidiabetic compounds from *Sarracenia purpurea* used traditionally by the Eeyou Istchee Cree First Nation. *J Nat Prod* 2012; **75**(7):1284-8. doi: 10.1021/np3001317.

711. Medina-Holguin AL, Holguin FO, Micheletto S *et al.* Chemotypic variation of essential oils in the medicinal plant, *Anemopsis californica*. *Phytochemistry* 2008; **69**(4):919-27. doi: 10.1016/j.phytochem.2007.11.006.

712. Zhuang T, Liang J-Y, Sun J-B *et al.* Secondary metabolites from *Saururus chinensis* and their chemotaxonomic significance. *Biochem Syst Ecol* 2014; **56**:95-98. doi: 10.1016/j.bse.2014.04.002.

713. Zhuang, T, Li, F, Huang, L *et al.* Secondary metabolites from the plants of the family Saururaceae and their biological properties. *Chemistry & Biodiversity* 2015; **12**:194-220.

714. Lai-King S, Richard SM, Geoffrey BD. Phytochemistry of *Illicium dunnianum* and the systematic position of the Illiciaceae. *Phytochemistry* 1997; **44**(6):1109-1108.

715. Yang JH, Pu JX, Wen J *et al.* Unusual cycloartane triterpenoids from *Kadsura ananosma*. *Phytochemistry* 2015; **109**:36-42. doi: 10.1016/j.phytochem.2014.10.014.

716. Blunden G, Patel AV, Armstrong N. Betaine distribution in the Scrophulariaceae and some previously included families. *Biochem Syst Ecol* 2003; **31**(4):359-365. doi: 10.1016/s0305-1978(02)00154-0.

717. Li Z-H, Long P, Bai S *et al.* Chemical constituents from *Cymbaria dahurica* L. (Scrophulariaceae). *Biochem Syst Ecol* 2014; **57**:11-14. doi: 10.1016/j.bse.2014.07.012.

718. Gousiadou C, Kokubun T, Gotfredsen CH *et al.* Further iridoid glucosides in the genus *Manulea* (Scrophulariaceae). *Phytochemistry* 2015; **109**:43-8. doi: 10.1016/j.phytochem.2014.10.004.

719. Albach DC, Li H-Q, Zhao N *et al.* Molecular systematics and phytochemistry of *Rehmannia* (Scrophulariaceae). *Biochem Syst Ecol* 2007; **35**(5):293-300. doi: 10.1016/j.bse.2006.11.003.

720. Jensen SR, Li HQ, Albach DC *et al.* Phytochemistry and molecular systematics of *Triaenophora rupestris* and *Oreosolen wattii* (Scrophulariaceae). *Phytochemistry* 2008; **69**(11):2162-6. doi: 10.1016/j.phytochem.2008.05.010.

721. Venditti A, Frezza C, Riccardelli M *et al.* Secondary metabolites from *Scrophularia canina* L. *Nat Prod Res* 2015:1-5. doi: 10.1080/14786419.2015.1122598.

722. Hans C. Krebs, Jeannot V. Rakotoarimanga, Rasoanaivo P *et al.* Alkaloids of *Perriera madagascariensis*. *J. Nat. Prod.* 1997; **60**:1183-1185.

723. Samuel L. Miller, Tinto WF, McLean S *et al.* Quassiols B-D, new squalene triterpenes from *Quassia multiflora*. *Tetrahedron* 1995; **51**(44):11959-11966.

724. Gibbons, S, Craven, L, Dunlop C *et al.* The secondary metabolites of aff. Samadera SAC-2825: An australian Simaroubaceae with unusual chemistry. *Phytochemistry* 1997; **44**(6):1109-1114.

725. Luisa Balderramaa, Alessandra Bracab, Garcia E *et al.* Triterpenes and anthraquinones from *Picramnia sellowii* Planchon in Hook (Simaroubaceae). *Biochem Syst Ecol* 2001; **29**:331-333.

726. Facundo, V, Azevedo, M, Rodrigues R *et al.* Chemical constituents from three medicinal plants: *Piper renitens*, *Siparuna guianensis* and *Alternanthera brasiliana*. *Revista Brasileira De Farmacognosia* 2012; **22**(5):1134-1139. doi: 10.1590/s0102695x2012005000040.

727. Nelita GcaFdB, Mir eacute ia ABP, Vany F *et al.* Antimicrobial activity and medicinal biomass of *Siparuna guianensis* in Brazilian Cerrado forest, a global hotspot. *J Med Plants Res* 2015; **9**(37):968-980. doi: 10.5897/jmpr2015.5884.

728. Fischer DC, de Amorim Gualda NC, Bachiega D *et al.* In vitro screening for antiplasmodial activity of isoquinoline alkaloids from Brazilian plant species. *Acta Trop* 2004; **92**(3):261-6. doi: 10.1016/j.actatropica.2004.08.009.

729. Zhang L, Liao CC, Huang HC *et al.* Antioxidant phenylpropanoid glycosides from *Smilax bracteata*. *Phytochemistry* 2008; **69**(6):1398-404. doi: 10.1016/j.phytochem.2008.01.002.

730. Wu LS, Wang XJ, Wang H *et al.* Cytotoxic polyphenols against breast tumor cell in *Smilax china* L. *J Ethnopharmacol* 2010; **130**(3):460-4. doi: 10.1016/j.jep.2010.05.032.

731. Li YL, Gan GP, Zhang HZ *et al.* A flavonoid glycoside isolated from *Smilax china* L. rhizome in vitro anticancer effects on human cancer cell lines. *J Ethnopharmacol* 2007; **113**(1):115-24. doi: 10.1016/j.jep.2007.05.016.

732. Shao B, Guo HZ, Cui YJ *et al.* Simultaneous determination of six major stilbenes and flavonoids in *Smilax china* by high performance liquid chromatography. *J Pharm Biomed Anal* 2007; **44**(3):737-42. doi: 10.1016/j.jpba.2007.03.008.

733. Shao B, Guo H, Cui Y *et al.* Steroidal saponins from *Smilax china* and their anti-inflammatory activities. *Phytochemistry* 2007; **68**(5):623-30. doi: 10.1016/j.phytochem.2006.10.026.

734. Zhang C-L, Gao J-M, Zhu W. Steroidal saponins from the rhizomes and roots of *Smilax scobinicaulis*. *Phytochemistry Letters* 2012; **5**(1):49-52. doi: 10.1016/j.phytol.2011.09.005.

735. Challinor VL, Parsons PG, Chap S *et al.* Steroidal saponins from the roots of *Smilax* sp.: structure and bioactivity. *Steroids* 2012; **77**(5):504-11. doi: 10.1016/j.steroids.2012.01.009.

736. Lu Y-Y, Luo J-G, Kong L-Y. Chemical constituents from *Solanum torvum*. *Chinese Journal of Natural Medicines* 2011; **9**(1):30-32. doi: 10.1016/s1875-5364(11)60015-0.

737. Tetenyi P. A chemotaxonomic classification of the Solanaceae. *Annals of the Missouri Botanical Garden* 1987; **74**(3):600-608.

738. Michael W. Evolution of secondary metabolites from an ecological and molecular phylogenetic perspective. *Phytochemistry* 2003; **64**(1):3-19. doi: 10.1016/s0031-9422(03)00300-5.

739. Hakkinen ST, Tilleman S, Swiatek A *et al.* Functional characterisation of genes involved in pyridine alkaloid biosynthesis in tobacco. *Phytochemistry* 2007; **68**(22-24):2773-85. doi: 10.1016/j.phytochem.2007.09.010.

740. Bertrand C, Comte G, Piola F. Solid-phase microextraction of volatile compounds from flowers of two *Brunfelsia* species. *Biochem Syst Ecol* 2006; **34**(5):371-375. doi: 10.1016/j.bse.2005.12.005.

741. Nakamura S, Hongo M, Sugimoto S *et al.* Steroidal saponins and pseudoalkaloid oligoglycoside from Brazilian natural medicine, "fruta do lobo" (fruit of *Solanum lycocarpum*). *Phytochemistry* 2008; **69**(7):1565-72. doi: 10.1016/j.phytochem.2008.02.003.

742. Jing-Jy Cheng, Li-Jie Zhang, Cheng H-L *et al.* Cytotoxic hexacyclic triterpene acids from *Euscaphis japonica*. *J. Nat. Prod.* 2010; **73**:1655-1658.

743. Ren-Wang Jiang, Po-Ming Hon, Zhou Y *et al.* Alkaloids and chemical diversity of *Stemona tuberosa* *J. Nat. Prod.* 2006; **69**(749-754).

744. Brem B, Seger C, Pacher T *et al.* Antioxidant dehydrotocopherols as a new chemical character of *Stemona species*. *Phytochemistry* 2004; **65**(19):2719-29. doi: 10.1016/j.phytochem.2004.08.023.

745. Mackova Z, Koblovska R, Lapcik O. Distribution of isoflavonoids in non-leguminous taxa - an update. *Phytochemistry* 2006; **67**(9):849-55. doi: 10.1016/j.phytochem.2006.01.020.

746. Kaltenegger E, Brem B, Mereiter K *et al.* Insecticidal pyrido[1,2-a]azepine alkaloids and related derivatives from *Stemona species*. *Phytochemistry* 2003; **63**(7):803-816. doi: 10.1016/s0031-9422(03)00332-7.

747. Jiang RW, Hon PM, Xu YT *et al.* Isolation and chemotaxonomic significance of tuberostemospironine-type alkaloids from *Stemona tuberosa*. *Phytochemistry* 2006; **67**(1):52-7. doi: 10.1016/j.phytochem.2005.10.004.

748. Zraunig A, Pacher T, Brecker L *et al.* Phenylbenzofuran-type stilbenoids from *Stemona* species. *Phytochemistry Letters* 2014; **9**:33-36. doi: 10.1016/j.phytol.2014.04.007.

749. Schinnerl J, Brem B, But PP *et al.* Pyrrolo- and pyridoazepine alkaloids as chemical markers in *Stemona* species. *Phytochemistry* 2007; **68**(10):1417-27. doi: 10.1016/j.phytochem.2007.03.002.

750. Kil Y-S, Han A-R, Seo EK. Tuberostemonine O from the roots of *Stemona tuberosa*. *Bulletin of the Korean Chemical Society* 2014; **35**(6):1891-1893. doi: 10.5012/bkcs.2014.35.6.1891.

751. Holscher D, Schneider B. Phenalenones from *Strelitzia reginae*. *J. Nat. Prod.* 2000; **63**:1027-1028.

752. Timmers MA, Guerrero-Medina JL, Esposito D *et al.* Characterization of phenolic compounds and antioxidant and anti-inflammatory activities from mamuyo (*Styrax ramirezii* Greenm.) fruit. *J Agric Food Chem* 2015; **63**(48):10459-65. doi: 10.1021/acs.jafc.5b04781.

753. Li J, Huang J, Ge J *et al.* Chemotaxonomic significance of n-alkane distributions from leaf wax in genus of *Sinojackia* species (Styracaceae). *Biochem Syst Ecol* 2013; **49**:30-36. doi: 10.1016/j.bse.2013.02.001.

754. Patrícia M. Pauletti, Helder L. Teles, Silva DHS *et al.* The Styracaceae. *Revista Brasileira de Farmacognosia Brazilian Journal of Pharmacognosy* 2006; **16**(4):576-590.

755. Feng Wang, Huiming Hua, Pei Y *et al.* Triterpenoids from the resin of *Styrax tonkinensis* and their antiproliferative and differentiation effects in human leukemia HL-60 cells. *J. Nat. Prod.* 2006; **69**(807-810).

756. Meijun Tang, Diandian Shen, Youcai Hu *et al.* Cytotoxic triterpenoid saponins from *Symplocos chinensis*. *J. Nat. Prod.* 2004; **67**:1969-1974.

757. Huo C, Liang H, Zhao Y *et al.* Neolignan glycosides from *Symplocos caudata*. *Phytochemistry* 2008; **69**(3):788-95. doi: 10.1016/j.phytochem.2007.08.022.

758. Acebey-Castellon IL, Voutquenne-Nazabadioko L, Doan Thi Mai H *et al.* Triterpenoid saponins from *Symplocos lancifolia*. *J Nat Prod* 2011; **74**(2):163-8. doi: 10.1021/np100502y.

759. Liu JB, Ding YS, Zhang Y *et al.* Anti-inflammatory hydrolyzable tannins from *Myricaria bracteata*. *J Nat Prod* 2015; **78**(5):1015-25. doi: 10.1021/np500953e.

760. Orabi MA, Taniguchi S, Sakagami H *et al.* Hydrolyzable tannins of tamaricaceous plants. V. Structures of monomeric-trimeric tannins and cytotoxicity of macrocyclic-type tannins isolated from *Tamarix nilotica*. *J Nat Prod* 2013; **76**(5):947-56. doi: 10.1021/np4001625.

761. Andhiwal, C, Kishore, K, Itoh, T *et al.* Unusually high ratio of saturated unsaturated sterols in *Tamarix gallica*. *J Nat Prod* 1982; **45**(222-223).

762. Sheng-Hong Li, Hong-Jie Zhang, Niu X-M *et al.* Chemical constituents from *Amentotaxus yunnanensis* and *Torreya yunnanensis*. *J. Nat. Prod.* 2003; **66**:1002-1005.

763. Li C, Huo C, Zhang M *et al.* Chemistry of Chinese yew, *Taxus chinensis* var. *mairei*. *Biochem Syst Ecol* 2008; **36**(4):266-282. doi: 10.1016/j.bse.2007.08.002.

764. Ge GB, Zhang YY, Hao DC *et al.* Chemotaxonomic study of medicinal *Taxus* species with fingerprint and multivariate analysis. *Planta Med* 2008; **74**(7):773-9. doi: 10.1055/s-2008-1074531.

765. Rozendaal ELMv, Kurstjens SJL, Beek TAv *et al.* Chemotaxonomy of *Taxus*. *Phytochemistry* 1999; **52**:427-433.

766. Huo C, Zhang X, Li C *et al.* A new taxol analogue from the leaves of *Taxus cuspidata*. *Biochem Syst Ecol* 2007; **35**(10):704-708. doi: 10.1016/j.bse.2007.03.005.

767. Zhang M, Dong M, Yang Y *et al.* A rare 3,8-seco-taxane from the leaves of *Taxus cuspidata*. *Biochem Syst Ecol* 2008; **36**(9):733-736. doi: 10.1016/j.bse.2008.03.012.

768. Huo C, Zhao Y, Si X *et al.* Two new taxanes from *Taxus canadensis*. *Biochem Syst Ecol* 2007; **35**(12):909-913. doi: 10.1016/j.bse.2007.05.006.

769. Gupta S, Gillett G. Chemotaxonomic studies of Hawaiian *Wikstroemia*. *Economic Botany* 1968; **23**:24-31.

770. Ko YC, Feng HT, Lee RJ *et al.* The determination of flavonoids in *Wikstroemia indica* C. A. Mey. by liquid chromatography with photo-diode array detection and negative electrospray ionization tandem mass spectrometry. *Rapid Commun Mass Spectrom* 2013; **27**(1):59-67. doi: 10.1002/rcm.6423.

771. Jiang H, Ma Q, Huang S *et al.* A new guaiane-type sesquiterpene with 15 known compounds from *Wikstroemia scytophylla* Diels. *Chinese Journal of Chemistry* 2012; **30**(6):1335-1338. doi: 10.1002/cjoc.201200011.

772. Xu W-C, Shen J-G, Jiang J-Q. Phytochemical and biological studies of the plants from the genus *Daphne*. *Chemistry & Biodiversity* 2011; **8**:1215-1233.

773. Pang X, Xie X. The pharmacological research progress of Thymelaceae. *J Henan Univ* 2007; **26**:9-11.

774. Bayer C, Appel O. Tropaeolaceae. *Kubitzki Klaus, Bayer Clemens (ed.) Flowering Plants. Springer-Verlag Berlin Heidelberg* 2003:400-404.

775. Greca M, Monaco P, Previtera L. Stigmasterols from *Typha latifolia*. *J Nat Prod* 1990; **53**(6):1430-1435.

776. Büchel K, Fenning T, Gershenzon J *et al.* Elm defence against herbivores and pathogens: morphological, chemical and molecular regulation aspects. *Phytochemistry Reviews* 2015. doi: 10.1007/s11101-015-9442-0.

777. Budzianowki, J, Skrzypczak, L, Walkowiak D. Flavonoids of *Parietaria officinalis*. *J Nat Prod* 1985; **48**(2):336-337.

778. Xing Fu Cai, Xuejun Jin, Dongho Lee *et al.* Phenanthroquinolizidine alkaloids from the roots of *Boehmeria pannosa* potently inhibit hypoxia-inducible factor-1 in AGS human gastric cancer cells. *J. Nat. Prod.* 2006; **69**:1095-1097.

779. Luca Rastrelli, Francesco De Simone, Gerardo Mora *et al.* Phenolic constituents of *Phenax angustifolius*. *J. Nat. Prod.* 2001; **64**:79-81.

780. Chaurasia N, Wichtl M. Sterols and steryl glycosides from *Urtica dioica*. *J Nat Prod* 1987; **50**(5):881-885.

781. Li J, Coleman CM, Wu H *et al.* Triterpenoids and flavonoids from *Cecropia schreberiana* Miq. (Urticaceae). *Biochem Syst Ecol* 2013; **48**:96-99. doi: 10.1016/j.bse.2012.11.018.

782. Franzyk H, Jensen SR, Olsen CE *et al.* A 9-Hydroxyiridoid Isolated from *Junellia seriphioides* (Verbenaceae). *Organic Letters* 2000; **2**(5):699-700. doi: 10.1021/ol0055521.

783. de Sena Filho JG, Rabbani ARC, dos Santos Silva TR *et al.* Chemical and molecular characterization of fifteen species from the *Lantana* (Verbenaceae) genus. *Biochem Syst Ecol* 2012; **45**:130-137. doi: 10.1016/j.bse.2012.07.024.

784. de Oliveira FC, Barbosa FG, Mafezoli J *et al.* Chemical constituents of *Lippia rigida* Schauer (Verbenaceae). *Biochem Syst Ecol* 2013; **51**:328-330. doi: 10.1016/j.bse.2013.09.009.

785. Reddy M, Mai M, Radhakrishnaiah M *et al.* Chemotaxonomy of *Viticeae* (Verbenaceae). *Feddes Repertorium* 1990; **101**(3-4):153-157.

786. Li Y, Li Z-H, Zhang C-H *et al.* Chemical constituents from *Vitis heyneana* Roem. & Schult (Vitaceae). *Biochem Syst Ecol* 2013; **50**:266-268. doi: 10.1016/j.bse.2013.04.012.

787. Wagner R, Simas FF, Pereira GCZ *et al.* Structure of a glycoglucuronomannan from the gum exudate of *Vochysia tucanorum* (family Vochysiaceae). *Carbohydrate Polymers* 2007; **69**(3):512-521. doi: 10.1016/j.carbpol.2007.01.005.

788. Araújo FW, Souza M, Filbo R. Vismiaefolic acid, a new triterpene from *Vochysia vismiaefolia*. *J Nat Prod* 1990; **53**(6):1436-1440.

789. Carnevale Neto F, Pilon AC, Silva DHS *et al.* Vochysiaceae: secondary metabolites, ethnopharmacology and pharmacological potential. *Phytochemistry Reviews* 2011; **10**(3):413-429. doi: 10.1007/s11101-011-9213-5.

790. Wayman KA, de Lange PJ, Larsen L *et al.* Chemotaxonomy of Pseudowintera: sesquiterpene dialdehyde variants are species markers. *Phytochemistry* 2010; **71**(7):766-72. doi: 10.1016/j.phytochem.2010.01.017.

791. Larsen L, Lorimer SD, Perry NB. Contrasting chemistry of fruits and leaves of two *Pseudowintera* species: Sesquiterpene dialdehyde cinnamates and prenylated flavonoids. *Biochem Syst Ecol* 2007; **35**(5):286-292. doi: 10.1016/j.bse.2006.11.001.

792. Zhong J, Huang Y, Ding W *et al.* Chemical constituents of *Aloe barbadensis* Miller and their inhibitory effects on phosphodiesterase-4D. *Fitoterapia* 2013; **91**:159-65. doi: 10.1016/j.fitote.2013.08.027.

793. Duewell H. Chemotaxonomy of the genus *Xanthorrhoea*. *Biochemical Systematics & Ecology* 1997; **25**(8):717-738.

794. Grace OM, Dzajic A, Jager AK *et al.* Monosaccharide analysis of succulent leaf tissue in *Aloe*. *Phytochemistry* 2013; **93**:79-87. doi: 10.1016/j.phytochem.2013.03.015.

795. O'Brien C, Van Wyk BE, Van Heerden FR. Physical and chemical characteristics of *Aloe ferox* leaf gel. *South African Journal of Botany* 2011; **77**(4):988-995. doi: 10.1016/j.sajb.2011.08.004.

796. Wu X, Ding W, Zhong J *et al.* Simultaneous qualitative and quantitative determination of phenolic compounds in *Aloe barbadensis* Mill by liquid chromatography-mass spectrometry-ion trap-time-of-flight and high performance liquid chromatography-diode array detector. *J Pharm Biomed Anal* 2013; **80**:94-106. doi: 10.1016/j.jpba.2013.02.034.

797. Barros Cota B, Braga de Oliveira A, Guilherme Guimarães K *et al.* Chemistry and antifungal activity of *Xyris* species (Xyridaceae): a new anthraquinone from *Xyris pilosa*. *Biochem Syst Ecol* 2004; **32**(4):391-397. doi: 10.1016/j.bse.2003.11.006.

798. Varanda EM, Rondinoni C, Yara Db *et al.* Flavonoids from *Xyris* species (Xyridaceae). *Biochem Syst Ecol* 2002; **30**:997-998.

799. Ruangrungsi N, Sekine T, Phadungcharoen T *et al.* Isocoumarins from *Xyris indica*. *Phytochemistry* 1995; **38**(2):481-483.

800. Manokam N, Nuntawong N. Chemical constituents from the rhizomes of *Globba reflexa* Craib. *Biochem Syst Ecol* 2014; **57**:395-398. doi: 10.1016/j.bse.2014.09.021.

801. Williams, C, Harborne J. The leaf flavonoids of the Zingiberales. *Biochemical Systematics & Ecology* 1997; **5**:221-229.

802. Kawasaki W, Matsui K, Akakabe Y *et al.* Volatiles from *Zostera marina*. *Phytochemistry* 1998; **47**(1):27-29.

803. Wu T-S, Shi L-S, Kuo S-C. Alkaloids and other constituents from *Tribulus terrestris*. *Phytochemistry* 1999; **50**:1411-1415.

804. Dinchev D, Janda B, Evstatieva L *et al.* Distribution of steroidal saponins in *Tribulus terrestris* from different geographical regions. *Phytochemistry* 2008; **69**(1):176-86. doi: 10.1016/j.phytochem.2007.07.003.

805. Hussein SR, Marzouk MM, Ibrahim LF *et al.* Flavonoids of *Zygophyllum album* L.f. and *Zygophyllum simplex* L. (Zygophyllaceae). *Biochem Syst Ecol* 2011; **39**(4-6):778-780. doi: 10.1016/j.bse.2011.07.009.

806. De Combarieu E, Fuzzati N, Lovati M *et al.* Furostanol saponins from *Tribulus terrestris*. *Fitoterapia* 2003; **74**(6):583-591. doi: 10.1016/s0367-326x(03)00152-7.

807. Mitra N, Mohammad-Mehdi D, Mohammad Reza Z. *Tribulus Terrestris* L. (Zygophyllaceae) flavonoid compounds. *International Journal of Modern Botany* 2012; **2**(3):35-39. doi: 10.5923/j.ijmb.20120203.01.

808. Chavez KJ, Feng X, Flanders JA *et al.* Spirocyclic lignans from *Guaiacum* (Zygophyllaceae) induce apoptosis in human breast cancer cell lines. *J Nat Prod* 2011; **74**(5):1293-7. doi: 10.1021/np100891y.

809. Wang Y, Ohtani K, Kasai R *et al.* Steroidal saponins from fruits of *Tribulus terrestris*. *Phytochemistry* 1996; **42**(5):1417-1422.

810. Duan J-A. Chemotaxonomic studies on flavonoids constitutes of Zygophyllaceae in China. *Acta Bot Boreal Occident Sin* 1999; **19**(4):725-731.

811. Maehara S, Simanjuntak P, Maetani Y *et al.* Ability of endophytic filamentous fungi associated with *Cinchona ledgeriana* to produce Cinchona alkaloids. *J Nat Med* 2013; **67**(2):421-3. doi: 10.1007/s11418-012-0701-8.

812. Erdemoglu N, Ozkan S, Tosun F. Alkaloid profile and antimicrobial activity of *Lupinus angustifolius* L. alkaloid extract. *Phytochemistry Reviews* 2007; **6**(1):197-201. doi: 10.1007/s11101-006-9055-8.

813. Pereira RM, Ferreira-Silva GA, Pivatto M *et al.* Alkaloids derived from flowers of *Senna spectabilis*, (-)-cassine and (-)-spectaline, have antiproliferative activity on HepG2 cells for inducing cell cycle arrest in G1/S transition through ERK inactivation and downregulation of cyclin D1 expression. *Toxicol In Vitro* 2016; **31**:86-92. doi: 10.1016/j.tiv.2015.11.018.

814. El Hamdani N, Fdil R, Tourabi M *et al.* Alkaloids extract of *Retama monosperma* (L.) Boiss. seeds used as novel eco-friendly inhibitor for carbon steel corrosion in 1M HCl solution: Electrochemical and surface studies. *Applied Surface Science* 2015; **357**:1294-1305. doi: 10.1016/j.apsusc.2015.09.159.

815. Guven KC, Percot A, Sezik E. Alkaloids in marine algae. *Mar Drugs* 2010; **8**(2):269-84. doi: 10.3390/md8020269.

816. Jiang H, Liu Y-B, Li Y *et al.* Analgesic corynanthe-type alkaloids from *Strychnos angustiflora*. *Tetrahedron* 2016; **72**(10):1276-1284. doi: 10.1016/j.tet.2015.11.011.

817. Hisiger S, Jolicoeur M. Analysis of *Catharanthus roseus* alkaloids by HPLC. *Phytochemistry Reviews* 2007; **6**(2-3):207-234. doi: 10.1007/s11101-006-9036-y.

818. Graziose R, Rathinasabapathy T, Lategan C *et al.* Antiplasmodial activity of aporphine alkaloids and sesquiterpene lactones from *Liriodendron tulipifera* L. *J Ethnopharmacol* 2011; **133**(1):26-30. doi: 10.1016/j.jep.2010.08.059.

819. Gravel E, Poupon E. Biosynthesis and biomimetic synthesis of alkaloids isolated from plants of the *Nitraria* and *Myrioneuron* genera: an unusual lysine-based metabolism. *Nat Prod Rep* 2010; **27**(1):32-56. doi: 10.1039/b911866g.

820. Kitajima M. Chemical studies on monoterpenoid indole alkaloids from medicinal plant resources *Gelsemium* and *Ophiorrhiza*. *Journal of Natural Medicines* 2006; **61**(1):14-23. doi: 10.1007/s11418-006-0101-z.

821. Pilli RA, Rosso GB, de Oliveira Mda C. The chemistry of stemona alkaloids: an update. *Nat Prod Rep* 2010; **27**(12):1908-37. doi: 10.1039/c005018k.

822. Waterman PG. Chemical Taxonomy of Alkaloids. In: Margaret F. Roberts, Wink Ms (ed.)*. Alkaloids: Biochemistry, Ecology, and Medicinal Applications*: Springer US, 1998, 87-107.

823. Khadem S, Marles RJ. Chromone and flavonoid alkaloids: occurrence and bioactivity. *Molecules* 2011; **17**(1):191-206. doi: 10.3390/molecules17010191.

824. Kobayashi J, Kubota T. The *Daphniphyllum* alkaloids. *Nat Prod Rep* 2009; **26**(7):936-62. doi: 10.1039/b813006j.

825. Hartmann T, Hggstrm Hk, Theuring C *et al.* Detoxification of pyrrolizidine alkaloids by the harvestman *Mitopus morio* (Phalangidae) a predator of alkaloid defended leaf beetles. *Chemoecology* 2003; **13**(3):123-127. doi: 10.1007/s00049-003-0236-2.

826. Soto IM, Carreira VP, Corio C *et al.* Differences in tolerance to host cactus alkaloids in *Drosophila koepferae* and *D. buzzatii*. *PLoS One* 2014; **9**(2): e88370. doi: 10.1371/journal.pone.0088370.

827. Freye E. The Different Types of Alkaloids in Coca. 2009:27-27. doi: 10.1007/978-90-481-2448-0_5.

828. Beaulieu WT, Panaccione DG, Hazekamp CS *et al.* Differential allocation of seed-borne ergot alkaloids during early ontogeny of morning glories (Convolvulaceae). *J Chem Ecol* 2013; **39**(7):919-30. doi: 10.1007/s10886-013-0314-z.

829. Ma Y, Mao XY, Huang LJ *et al.* Diterpene alkaloids and diterpenes from *Spiraea japonica* and their anti-tobacco mosaic virus activity. *Fitoterapia* 2016; **109**:8-13. doi: 10.1016/j.fitote.2015.11.019.

830. Wang FP, Chen QH, Liu XY; Diterpenoid alkaloids. *Nat Prod Rep* 2010; **27**(4):529-70. doi: 10.1039/b916679c.

831. Hol WH. The effect of nutrients on pyrrolizidine alkaloids in Senecio plants and their interactions with herbivores and pathogens. *Phytochem Rev* 2011; **10**(1):119-126. doi: 10.1007/s11101-010-9188-7.

832. Raj D, Kokotkiewicz A, Drys A *et al.* Effect of plant growth regulators on the accumulation of indolizidine alkaloids in Securinega suffruticosa callus cultures. *Plant Cell, Tissue and Organ Culture (PCTOC)* 2015; **123**(1):39-45. doi: 10.1007/s11240-015-0811-6.

833. Bienaimé C, Melin A, Bensaddek L *et al.* Effects of plant growth regulators on cell growth and alkaloids production by cell cultures of *Lycopodiella inundata*. *Plant Cell, Tissue and Organ Culture (PCTOC)* 2015; **123**(3):523-533. doi: 10.1007/s11240-015-0856-6.

834. Kucht S, Gross J, Hussein Y *et al.* Elimination of ergoline alkaloids following treatment of *Ipomoea asarifolia* (Convolvulaceae) with fungicides. *Planta* 2004; **219**(4):619-25. doi: 10.1007/s00425-004-1261-2.

835. Wallwey C, Li SM. Ergot alkaloids: structure diversity, biosynthetic gene clusters and functional proof of biosynthetic genes. *Nat Prod Rep* 2011; **28**(3):496-510. doi: 10.1039/c0np00060d.

836. Kadam PD, Chuan HH. Erratum to: Rectocutaneous fistula with transmigration of the suture: a rare delayed complication of vault fixation with the sacrospinous ligament. *Int Urogynecol J* 2016; **27**(3):505. doi: 10.1007/s00192-016-2952-5.

837. Naumann C, Hartmann T, Ober D. Evolutionary recruitment of a flavin-dependent monooxygenase for the detoxification of host plant-acquired pyrrolizidine alkaloids in the alkaloid-defended arctiid moth *Tyria jacobaeae*. *Proc Natl Acad Sci U S A* 2002; **99**(9):6085-90. doi: 10.1073/pnas.082674499.

838. Dutta A, Batra J, Pandey-Rai S *et al.* Expression of terpenoid indole alkaloid biosynthetic pathway genes corresponds to accumulation of related alkaloids in *Catharanthus roseus* (L.) G. Don. *Planta* 2005; **220**(3):376-83. doi: 10.1007/s00425-004-1380-9.

839. Chang A, Cai Z, Wang Z *et al.* Extraction and isolation of alkaloids of *Sophora alopecuroides* and their anti-tumor effects in h22 tumor-bearing mice. *African Journal of Traditional, Complementary and Alternative Medicines* 2014; **11**(2):245. doi: 10.4314/ajtcam.v11i2.3.

840. Cordell GA. Fifty years of alkaloid biosynthesis in Phytochemistry. *Phytochemistry* 2013; **91**:29-51. doi: 10.1016/j.phytochem.2012.05.012.

841. Pelser PB, de Vos H, Theuring C *et al.* Frequent gain and loss of pyrrolizidine alkaloids in the evolution of Senecio section Jacobaea (Asteraceae). *Phytochemistry* 2005; **66**(11):1285-95. doi: 10.1016/j.phytochem.2005.04.015.

842. Duarte P, Memelink J, Sottomayor M. Fusion with fluorescent proteins for subcellular localization of enzymes involved in plant alkaloid biosynthesis. *Methods Mol Biol* 2010; **643**:275-90. doi: 10.1007/978-1-60761-723-5_19.

843. Lovkova MY, Buzuk GN, Sokolova SM. Genetic aspects of the interrelation between alkaloids and chemical elements in *Atropa belladonna* L. and Glaucium flavum Crantz. Plants. *Applied Biochemistry and Microbiology* 2008; **44**(4):416-419. doi: 10.1134/s0003683808040145.

844. Yadav HK, Shukla S, Singh SP. Genetic variability and interrelationship among opium and its alkaloids in opium poppy (*Papaver Somniferum* L.). *Euphytica* 2006; **150**(1-2):207-214. doi: 10.1007/s10681-006-9111-x.

845. Sun B, Zhang F, Zhou GJ *et al.* Genetic variation in alkaloid accumulation in leaves of Nicotiana. *J Zhejiang Univ Sci B* 2013; **14**(12):1100-9. doi: 10.1631/jzus.B1300130.

846. He JY, Ma N, Zhu S *et al.* The genus *Codonopsis* (Campanulaceae): a review of phytochemistry, bioactivity and quality control. *J Nat Med* 2015; **69**(1):1-21. doi: 10.1007/s11418-014-0861-9.

847. Torres MS, White JF. Grass endophyte-mediated plant stress tolerance: alkaloids and their functions. 2010; **17**:477-493. doi: 10.1007/978-90-481-9449-0_24.

848. Kumar S, Singh A, Bajpai V *et al.* Identification, characterization and distribution of monoterpene indole alkaloids in Rauwolfia species by Orbitrap Velos Pro mass spectrometer. *J Pharm Biomed Anal* 2016; **118**:183-94. doi: 10.1016/j.jpba.2015.10.037.

849. Panjikar S, Stoeckigt J, O'Connor S *et al.* The impact of structural biology on alkaloid biosynthesis research. *Nat Prod Rep* 2012; **29**(10):1176-200. doi: 10.1039/c2np20057k.

850. Michael JP. Indolizidine and quinolizidine alkaloids. *Nat Prod Rep* 2005; **22**(5):603-26. doi: 10.1039/b413748p.

851. Zeng J, Liu Y, Liu W *et al.* Integration of transcriptome, proteome and metabolism data reveals the alkaloids biosynthesis in *Macleaya cordata* and *Macleaya microcarpa*. *PLoS One* 2013; **8**(1): e53409. doi: 10.1371/journal.pone.0053409.

852. Hank H, Szke, Tth K *et al.* Investigation of tropane alkaloids in genetically transformed *Atropa belladonna* L. cultures. *Chromatographia* 2004. doi: 10.1365/s10337-004-0240-x.

853. Bogdanov MG, Keremedchieva R, Svinyarov I. Ionic liquid-supported solid–liquid extraction of bioactive alkaloids. III. Ionic liquid regeneration and glaucine recovery from ionic liquid-aqueous crude extract of *Glaucium flavum* Cr. (Papaveraceae). *Separation and Purification Technology* 2015; **155**:13-19. doi: 10.1016/j.seppur.2015.02.003.

854. Zhang R-R, Tian H-Y, Wu Y *et al.* Isolation and chemotaxonomic significance of stenine- and stemoninine-type alkaloids from the roots of *Stemona tuberosa*. *Chinese Chemical Letters* 2014; **25**(9):1252-1255. doi: 10.1016/j.cclet.2014.03.051.

855. Reina L, Bennadji Z, Vinciguerra V *et al.* Isolation and structural characterization of new piperidine alkaloids from *Prosopis affinis*. *Phytochemistry Letters* 2015; **14**:265-269. doi: 10.1016/j.phytol.2015.10.022.

856. Kang KB, Ming G, Kim GJ *et al.* Jubanines F-J, cyclopeptide alkaloids from the roots of *Ziziphus jujuba*. *Phytochemistry* 2015; **119**:90-5. doi: 10.1016/j.phytochem.2015.09.001.

857. Bunsupa S, Katayama K, Ikeura E *et al.* Lysine decarboxylase catalyzes the first step of quinolizidine alkaloid biosynthesis and coevolved with alkaloid production in leguminosae. *Plant Cell* 2012; **24**(3):1202-16. doi: 10.1105/tpc.112.095885.

858. Sato F, Hashimoto T, Hachiya A *et al.* Metabolic engineering of plant alkaloid biosynthesis. *Proc Natl Acad Sci U S A* 2001; **98**(1):367-72. doi: 10.1073/pnas.011526398.

859. Jin Z. Muscarine, imidaozle, oxazole and thiazole alkaloids. *Nat Prod Rep* 2013; **30**(6):869-915. doi: 10.1039/c3np70006b.

860. Adler LS, Irwin RE. Nectar alkaloids decrease pollination and female reproduction in a native plant. *Oecologia* 2012; **168**(4):1033-41. doi: 10.1007/s00442-011-2153-3.

861. Zhang J, Huang ZH, Qiu XH *et al.* Neutral fragment filtering for rapid identification of new diester-diterpenoid alkaloids in roots of *Aconitum carmichaeli* by ultra-high-pressure liquid chromatography coupled with linear ion trap-orbitrap mass spectrometry. *PLoS One* 2012; **7**(12): e52352. doi: 10.1371/journal.pone.0052352.

862. Cheng Y-B, Lo IW, Shyur L-F *et al.* New alkaloids from formosan zoanthid *Zoanthus kuroshio*. *Tetrahedron* 2015; **71**(45):8601-8606. doi: 10.1016/j.tet.2015.09.023.

863. Iqbal N, Adhikari A, Kanwal N *et al.* New immunomodulatory steroidal alkaloids from *Sarcococa saligna*. *Phytochemistry Letters* 2015; **14**:203-208. doi: 10.1016/j.phytol.2015.10.009.

864. Busque J, Pedrosa MM, Cabellos B *et al.* Phenological changes in the concentration of alkaloids of *Carex brevicollis* in an Alpine rangeland. *J Chem Ecol* 2010; **36**(11):1244-54. doi: 10.1007/s10886-010-9865-4.

865. Hartmann T, Theuring C, Beuerle T *et al.* Phenological fate of plant-acquired pyrrolizidine alkaloids in the polyphagous arctiid Estigmene acrea. *Chemoecology* 2004; **14**(3-4). doi: 10.1007/s00049-004-0276-2.

866. Kiani M, Sefidkon F, Babaei A *et al.* Phytochemical profiling of medicinal isosteroidal alkaloids of Iranian *Fritillaria* spp. (Liliaceae). *Industrial Crops and Products* 2015; **70**:451-458. doi: 10.1016/j.indcrop.2015.03.064.

867. Zhang P, Shao L, Shi Z *et al.* Pregnane alkaloids from *Sarcococca ruscifolia* and their cytotoxic activity. *Phytochemistry Letters* 2015; **14**:31-34. doi: 10.1016/j.phytol.2015.08.010.

868. Dehghan E, Häkkinen ST, Oksman-Caldentey K-M *et al.* Production of tropane alkaloids in diploid and tetraploid plants and in vitro hairy root cultures of Egyptian henbane (*Hyoscyamus muticus* L.). *Plant Cell, Tissue and Organ Culture (PCTOC)* 2012; **110**(1):35-44. doi: 10.1007/s11240-012-0127-8.

869. Kamel KA, Święcicki W, Kaczmarek Z *et al.* Quantitative and qualitative content of alkaloids in seeds of a narrow-leafed lupin (*Lupinus angustifolius* L.) collection. *Genetic Resources and Crop Evolution* 2015; **63**(4):711-719. doi: 10.1007/s10722-015-0278-7.

870. Michael JP. Quinoline, quinazoline and acridone alkaloids. *Nat Prod Rep* 2004; **21**(5):650-68. doi: 10.1039/b310691h.

871. Naeem M, Aftab T, Ansari AA *et al.* Radiolytically degraded sodium alginate enhances plant growth, physiological activities and alkaloids production in *Catharanthus roseus* L. *Journal of Radiation Research and Applied Sciences* 2015; **8**(4):606-616. doi: 10.1016/j.jrras.2015.07.005.

872. Robert RF. *Plant alkaloids: a guide to their discovery and distribution.* New York, NY: Food Products Press, 1996.

873. Kang YM, Min JY, Moon HS *et al.* Rapid in vitro adventitious shoot propagation of Scopolia parviflora through rhizome cultures for enhanced production of tropane alkaloids. *Plant Cell Rep* 2004; **23**(3):128-33. doi: 10.1007/s00299-004-0820-0.

874. Glenn WS, Runguphan W, O'Connor SE. Recent progress in the metabolic engineering of alkaloids in plant systems. *Curr Opin Biotechnol* 2013; **24**(2):354-65. doi: 10.1016/j.copbio.2012.08.003.

875. del Campo ML, Smedley SR, Eisner T. Reproductive benefits derived from defensive plant alkaloid possession in an arctiid moth (*Utetheisa ornatrix*). *Proc Natl Acad Sci U S A* 2005; **102**(38):13508-12. doi: 10.1073/pnas.0505725102.

876. Schrittwieser JH, Resch V. The role of biocatalysis in the asymmetric synthesis of alkaloids. *RSC Adv* 2013; **3**(39):17602-17632. doi: 10.1039/c3ra42123f.

877. Bakri M, Chen Q, Ma Q *et al.* Separation and purification of two new and two known alkaloids from leaves of *Nitraria sibirica* by pH-zone-refining counter-current chromatography. *J Chromatogr B Analyt Technol Biomed Life Sci* 2015; **1006**:138-45. doi: 10.1016/j.jchromb.2015.10.038.

878. Zhang P-t, Pan B-y, Liao Q-f *et al.* Separation of five quinolone alkaloids from fruits of *Evodia rutaecarpa* by high-speed counter-current chromatography. *Chinese Herbal Medicines* 2014; **6**(1):47-52. doi: 10.1016/s1674-6384(14)60006-x.

879. Ishikura M, Abe T, Choshi T *et al.* Simple indole alkaloids and those with a nonrearranged monoterpenoid unit. *Nat Prod Rep* 2015; **32**(10):1389-471. doi: 10.1039/c5np00032g.

880. Kikura-Hanajiri R, Kawamura M, Maruyama T *et al.* Simultaneous analysis of mitragynine, 7-hydroxymitragynine, and other alkaloids in the psychotropic plant “kratom” (*Mitragyna speciosa*) by LC-ESI-MS. *Forensic Toxicology* 2009; **27**(2):67-74. doi: 10.1007/s11419-009-0070-5.

881. Joosten L, Mulder PPJ, Klinkhamer PGL *et al.* Soil-borne microorganisms and soil-type affect pyrrolizidine alkaloids in *Jacobaea vulgaris*. *Plant and Soil* 2009; **325**(1-2):133-143. doi: 10.1007/s11104-009-9963-7.

882. Wang LL, Kakiuchi N, Mikage M. Studies of *Ephedra* plants in Asia. Part 6: Geographical changes of anatomical features and alkaloids content of *Ephedra sinica*. *J Nat Med* 2010; **64**(1):63-9. doi: 10.1007/s11418-009-0374-0.

883. Sun Y, Peng T, Zhao L *et al.* Studies of interaction between two alkaloids and double helix DNA. *Journal of Luminescence* 2014; **156**:108-115. doi: 10.1016/j.jlumin.2014.07.014.

884. Singh S, Verma SK. Study of the distribution profile of piperidine alkaloids in various parts of *Prosopis juliflora* by the application of Direct Analysis in Real Time Mass Spectrometry (DART-MS). *Natural Products and Bioprospecting* 2012; **2**(5):206-209. doi: 10.1007/s13659-012-0069-1.

885. Jiang P, Liu H, Xu X *et al.* Three new alkaloids and three new phenolic glycosides from *Liparis odorata*. *Fitoterapia* 2015; **107**:63-8. doi: 10.1016/j.fitote.2015.10.003.

886. Takos AM, Rook F. Towards a molecular understanding of the biosynthesis of amaryllidaceae alkaloids in support of their expanding medical use. *Int J Mol Sci* 2013; **14**(6):11713-41. doi: 10.3390/ijms140611713.

887. Yu F, De Luca V; Transport of monoterpenoid indole alkaloids in *Catharanthus roseus*. 2014; **22**:63-75. doi: 10.1007/978-3-319-06511-3_5.

888. Zhou R, Duan J. *Plant Chemotaxonomy.* Shanghai, China: Shanghai science and Technology Press, 2005.

889. Gould K, Davies K, Winefield C. *Anthocyanins: Biosynthesis, Functions, and Applications.* Springer-Verlag New York: Springer-Verlag New York, 2009.

890. Häkkinen S, Kärenlampi S, Heinonen I *et al.* Content of the Flavonols Quercetin, Myricetin, and Kaempferol in 25 Edible Berries. *J Agr Food Chem*1994; **47**(6):2274-2279.

891. Crawford DJ. Flavonoid chemistry and angiosperm evolution. *The Botanical Review* 1978; **44**(4):431-456.

892. Iwashina T. The structure and distribution of the flavonoids in plants. *Journal of Plant Research* 2000; **113**(3):287-299.

893. Sultanbawa M. Xanthonoids of tropical plants. *Tetrahedron* 1980; **36**(11):1465-1506.

894. Lapcik O. Isoflavonoids in non-leguminous taxa: a rarity or a rule? *Phytochemistry* 2007; **68**(22-24):2909-16. doi: 10.1016/j.phytochem.2007.08.006.

895. Marin P. Flavonoids as taxonomic markers in flowering plants. *Glas Inst Bot I Baste Univ U Beoggradu* 1996; **30**:19-37.

896. Veitch NC, Grayer RJ. Flavonoids and their glycosides, including anthocyanins. *Nat Prod Rep* 2008; **25**(3):555-611. doi: 10.1039/b718040n.

897. Ventura CP, Dias de Souza Filho J, Braga de Oliveira A *et al.* A flavanone and other constituents of the Brazilian endemic species *Trembleya laniflora* (D. Don) Cogn. (Melastomataceae). *Biochem Syst Ecol* 2007; **35**(1):40-41. doi: 10.1016/j.bse.2006.08.001.

898. Seigler DS. *Plant secondary metabolism.* New York: Springer Science & Business Media, 1998.

899. Williams CA, Grayer RJ. Anthocyanins and other flavonoids. *Natural Product Reports* 2004; **21**:539-573. doi: 10.1039/.

900. L Coradin, DE Giannasi, Prance G. Chemosystematic studies in the Chrysobalanaceae. I. Flavonoids in Parinari. *Brittonia* 1985; **37**(2):169-178.

901. Jay M. Chemotaxonomic researches on vascular plants. XIX. Flavonoid distribution in the Pittosporaceae. *Botanical Journal of the Linnean Society* 1969; **62**(4):423-429.

902. Ping NHS. Distribution and taxonomic significance of flavonoids in the Olacaceae and Icacinaceae. *Biochemical Systematics & Ecology* 1997; **25**:263-265.

903. Valderrama J. Distribution of flavonoids in the Myristicaceae. *Phytochemistry* 2000; **55**:505-511.

904. BA Bohm, A Reid, M Devore *et al.* Flavonoid chemistry of Calyceraceae. *Canadian Journal of Botany* 2011; **73**(12):1962-1965.

905. Doyle JJ. Flavonoid Races of *Claytonia virginica* (Portulacaceae). *Am J Bot*1983; **70**(7):1085-1091.

906. P Mann, B Tofern, M Kaloga *et al.* Flavonoid sulfates from the Convolvulaceae. *Phytochemistry* 1999; **50**(2):267-271.

907. Harborne J. Flavonoids and the evolution of the angiosperms. *Biochemical Systematics & Ecology* 1977; **5**:7-22.

908. K Likhitwitayawuid, R Rungserichai, N Ruangrungsi *et al.* Flavonoids from *Ochna integerrima*. *Phytochemistry* 2001; **56**(4):353-357.

909. EM Varanda, C Rondinoni, Santos D. Flavonoids from *Xyris* species (Xyridaceae). *Biochemical Systematics & Ecology* 2002; **30**:997-998.

910. M Sharaf, MA El-Ansari, Saleh N. Flavonoids of four *Cleome* and three *Capparis* species. *Biochemical Systematics & Ecology* 1997; **25**(2):161-166.

911. DYAC Santos, MLF Salatino, Salatino A. Flavonoids of species of *Cuphea* (Lythraceae) from Brazil. *Biochemical Systematics & Ecology* 1995; **23**(1):99-103.

912. V.M. Malikov, V.P. Bruskov, M.P. Yuldashev *et al. Flavonoids: Plant Sources, Structure and Properties.* Springer Science+Business Media New York: Springer-Verlag New York, 2013.

913. M Noori, MM Dehshiri, Ghorbani M. Investigation of leaf flavonoids of *Reseda* (Tourn.) et L. (Resedaceae). *Journal of Medicinal Plants and By-Products,* 2012; **2**:171-176.

914. Harborne JB. *The Flavonoids: Advances in Research since 1980.* Springer Science+Business Media Dordrecht: Springer US, 1980.

915. Chen Bhslbib. Simultaneous determination of phenolic acids and flavonoids in *Chenopodium formosanum* Koidz. (djulis) by HPLC-DAD-ESI-MS/MS. *J Pharm Biomed Anal* 2016; **132**:109-116. doi: 10.1016/j.jpba.2016.09.027.

916. Harborne J. A chemotaxonomic survey of flavonoids in leaves of the Oleaceae. *Botanical Journal of the Linnean Society* 1980; **81**:155-167.

917. Koes R, Verweij W, Quattrocchio F. Flavonoids: a colorful model for the regulation and evolution of biochemical pathways. *Trends Plant Sci* 2005; **10**(5):236-42. doi: 10.1016/j.tplants.2005.03.002.

918. Li J, Coleman CM, Wu H *et al.* Triterpenoids and flavonoids from *Cecropia schreberiana* Miq. (Urticaceae). *Biochem Syst Ecol* 2013; **48**:96-99. doi: 10.1016/j.bse.2012.11.018.

919. Li Y-L, Gan G-P, Zhang H-Z *et al.* A flavonoid glycoside isolated from *Smilax china* L. rhizome in vitro anticancer effects on human cancer cell lines. *Journal of Ethnopharmacology* 2007; **113**(1):115-124. doi: 10.1016/j.jep.2007.05.016.

920. Mitra N, Mohammad-Mehdi D, Mohammad Reza Z. *Tribulus Terrestris* L. (Zygophyllaceae) flavonoid compounds. *International Journal of Modern Botany* 2012; **2**(3):35-39. doi: 10.5923/j.ijmb.20120203.01.

921. Onyilagha J, Bala A, Hallett R *et al.* Leaf flavonoids of the cruciferous species, *Camelina sativa,* *Crambe* spp., *Thlaspi arvense* and several other genera of the family Brassicaceae. *Biochem Syst Ecol* 2003; **31**(11):1309-1322. doi: 10.1016/s0305-1978(03)00074-7.

922. Llorent-Martínez EJ, Gouveia S, Castilho PC. Analysis of phenolic compounds in leaves from endemic trees from Madeira Island. A contribution to the chemotaxonomy of Laurisilva forest species. *Industrial Crops and Products* 2015; **64**:135-151. doi: 10.1016/j.indcrop.2014.10.068.

923. Mudnic I, Modun D, Rastija V *et al.* Antioxidative and vasodilatory effects of phenolic acids in wine. *Food Chemistry* 2010; **119**(3):1205-1210. doi: 10.1016/j.foodchem.2009.08.038.

924. Heleno SA, Martins A, Queiroz MJ *et al.* Bioactivity of phenolic acids: metabolites versus parent compounds: a review. *Food Chem* 2015; **173**:501-13. doi: 10.1016/j.foodchem.2014.10.057.

925. Ross KA, Beta T, Arntfield SD. A comparative study on the phenolic acids identified and quantified in dry beans using HPLC as affected by different extraction and hydrolysis methods. *Food Chem* 2009; **113**(1):336-344. doi: 10.1016/j.foodchem.2008.07.064.

926. M. Nardini, E. Cirillo, F. Natella *et al.* Detection of bound phenolic acids: prevention by ascorbic acid and ethylenediaminetetraacetic acid of degradation of phenolic acids during alkaline hydrolysis. *Food Chemistry* 2002; **79**:119-124.

927. Määttä-Riihinen, K, Kamal-Eldin, A, Mattila, P *et al.* Distribution and contents of phenolic compounds in eighteen Scandinavian berry species. *J Agr Food Chem*2004; **52**(14):4477-4486.

928. Zhongkai Zhou, Kevin Robards, Stuart Helliwell *et al.* The distribution of phenolic acids in rice. *Food Chemistry* 2004; **87**:401-406. doi: 10.1016/j.foodchem.2003.12.015.

929. Luthria DL, Pastor-Corrales MA. Phenolic acids content of fifteen dry edible bean (*Phaseolus vulgaris* L.) varieties. *J Food Compos Anal* 2006; **19**(2):205-211. doi: 10.1016/j.jfca.2005.09.003.

930. Fernanda M.F. Roleira, Elisiário J. Tavares-da-Silva, Carla L. Varela a SCC *et al.* Plant derived and dietary phenolic antioxidants: Anticancer properties. *Food Chemistry* 2015; **183**:235-258. doi: 10.1016/j.foodchem.2015.03.039.

931. Agrawal AA, Salminen JP, Fishbein M. Phylogenetic trends in phenolic metabolism of milkweeds (*Asclepias*): evidence for escalation. *Evolution* 2009; **63**(3):663-73. doi: 10.1111/j.1558-5646.2008.00573.x.

932. Boudet AM. Evolution and current status of research in phenolic compounds. *Phytochemistry* 2007; **68**(22-24):2722-35. doi: 10.1016/j.phytochem.2007.06.012.

933. Ibrahim RK, Stafford HA. *Phenolic Metabolism in Plants.* Springer Science+Business Media New York: Springer US, 1992.

934. Šamec D, Valek-Žulj L, Martinez S *et al.* Phenolic acids significantly contribute to antioxidant potency of *Gynostemma pentaphyllum* aqueous and methanol extracts. *Industrial Crops & Products* 2016; **84**:104-107. doi: 10.1016/j.indcrop.2016.01.035.

935. T Swain, JB Harborne, Sumere C. *Biochemistry of Plant Phenolics.* New York and London: Plenum Press, 1979.

936. Vítor Spínola, Joana Pinto, Castilho PC. Identification and quantification of phenolic compounds of selected fruits from Madeira Island by HPLC-DAD–ESI-MSn and screening for their antioxidant activity. *Food Chemistry 1* 2015; **173**:14-30. doi: 10.1016/j.foodchem.2014.09.163.

937. A Mohagheghzadeh, S Hemmati, I Mehregan *et al.* Linum persicum: Lignans and placement in Linaceae *Phytochemistry Reviews* 2003; **2**(3):363-369.

938. Bagniewska-Zadworna, Barakat, Lakomy *et al.* Lignin and lignans in plant defence: insight from expression profiling of cinnamyl alcohol dehydrogenase genes during development and following fungal infection in *Populus*. *Plant Science* 2014; **229**:111-121. doi: 10.1016/j.plantsci.2014.08.015.

939. BS Min, MK Na, SR Oh *et al.* New furofuran and butyrolactone lignans with antioxidant activity from the stem bark of *Styrax japonica*. *J Nat Prod* 2011; **67**:1980-1984.

940. F Kawamura, S Kawai, Ohashi H. Sesquilignans and lignans from *Tsuga heterophylla*. *Phytochemistry* 1997; **44**:1351-1357.

941. G Dad, A Corbani, P Manitto *et al.* Lignan glycosides from the heartwood of European oak *Quercus petraea*. *J Nat Prod* 2004; **52**(6):1327-1330.

942. Hokanson G. The lignans of *Polygala polygama* (Polygalaceae): deoxypodophyllotoxin and three new lignan lactones. *J Nat Prod* 1979; **42**:378-384.

943. IK Park, SC Shin, CS Kim *et al.* Larvicidal Activity of Lignans Identified in *Phryma leptostachya* var. *asiatica* roots against three mosquito species. *J Agr Food Chem*2005; **53**:969-972.

944. J Liu, L Wang, D Zhao *et al.* A new lignan from *Saururus chinensis*. *Chemistry of Natural Compounds* 2010; **46**(4):631-633.

945. JBG Siqueira, MDGB Zoghbi, JA Cabral *et al.* Lignans from *Protium tenuifolium*. *J Nat Prod* 2004; **58**(5):730-732.

946. KR Rezende, Kato M. Dibenzylbutane and aryltetralone lignans from seeds of *Virola sebifera*. *Phytochemistry* 2002; **61**(4):427-432.

947. L Pistelli, R Venturi, A Marsili *et al.* Alkaloids and coumarins from *Gymnosporia senegalensis* var. *spinosa* (Celastraceae). *Biochemical Systematics & Ecology* 1998; **26**(6):677-679.

948. N Turgutkara, ÇAkır Ö. Comparative phylogenetic analysis of phenylpropanoid metabolism genes of legume plants. *Plant Omics* 2015; **8**(1):55-61.

949. Norberto Peporine Lopes, Ema Ester de Almeida Blumenthal, Alberto José Cavalheiro *et al.* Lignans, γ-lactones and propiophenones of *Virola surinamensis*. *Phytochemistry* 1996; **43**(5):1089-1092.

950. Q Song, FR Fronczek, Fischer N. Dibenzocyclooctadiene-type lignans from *Magnolia pyramidata*. *Phytochemistry* 2000; **55**(6):653-661.

951. R Torres, A Urzua, Modak B. Isopregomisin, a 1, 4-Bis(phenyl)-2, 3-dimethylbutane Lignan from *Porlieria chilensis*. *J Nat Prod* 1989; **52**(2):402-403.

952. S Cowan, B Bartholomew, AA Watson *et al.* Lignans from *Cupressus lusitanica* (Cupressaceae). *Biochemical Systematics & Ecology* 2001; **29**:109-111.

953. S Liao, Y Wu, Yue J. Lignans from *Wikstroemia hainanensis*. *Cheminform* 2006; **37**(4):73-80.

954. S Susplugas, NV Hung, J Bignon *et al.* Cytotoxic arylnaphthalene lignans from a Vietnamese Acanthaceae, *Justicia patentiflora*. *J Nat Prod* 2005; **68**(5):734-738.

955. SG Cao, KY Sim, J Pereira *et al.* Coumarins from *Calophyllum teysmannii. Phytochemistry* 1998; **47**(6):1051-1055.

956. SM Nunomura, Yoshida M. Lignans and benzoic acid derivatives from pericarps of *Virola multinervia* (Myristicaceae). *Biochemical Systematics & Ecology* 2002; **30**(10):985-987.

957. T Kanchanapoom, MS Kamel, R Kasai *et al.* Lignan glucosides from *Acanthus ilicifolius*. *Phytochemistry* 2001; **56**(4):369-372.

958. T Kosuge, Conn EE. The metabolism of aromatic compounds in higher plants. I. Coumarin and o-coumaric acid. *Journal of Biological Chemistry* 1959; **234**:2133-2137.

959. T Okunishi, T Umezawa, Shimada M. Isolation and enzymatic formation of lignans of *Daphne genkwa* and *Daphne odora*. *Journal of Wood Science* 2001; **47**(5):383-388.

960. Z Latif, TG Hartley, MJ Rice *et al.* Isobutylamides and coumarins from *Melicope melanophloia*. *Biochemical Systematics & Ecology* 1998; **26**(4):467-468.

961. ZH Jiang, T Tanaka, H Iwata *et al.* Ellagitannins and Lignan Glycosides from *Balanophora japonica* (Balanophoraceae). *Cheminform* 2005; **53**(3):339-341.

962. Beatrycze Nowicka, Kruk J. Occurrence, biosynthesis and function of isoprenoid quinones. *BBA* 2010; **1797**:1587-1605. doi: 10.1016/j.bbabio.2010.06.007.

963. Gaunt, JK, Stowe BB. Analysis and distribution of tocopherols and quinones in the pea plant. *Plant Physiology* 1967; **42**(6):851-858.

964. J Avilla, A Teixidò, C Velázquez *et al.* Insecticidal activity of *Maytenus* species (Celastraceae) nortriterpene quinone methides against codling moth, *Cydia pomonella* (L.) (Lepidoptera: Tortricidae). *J Agr Food Chem*2000; **48**(1):88-92.

965. Kruk J, Strzałka K. Occurrence and function of α-tocopherol quinone in plants. *J Plant Physiol* 1995; **145**(4):405-409. doi: 10.1016/S0176-1617(11)81762-1.

966. Lederer E. The origin and function of some methyl groups in branched-chain fatty acids, plant sterols and quinones. *Biochemical Journal* 1964; **93**:449-468.

967. Luigi Lucini, Marco Pellizzoni, Molinari GP. Anthraquinones and β-polysaccharides content and distribution in *Aloe* plants grown under different light intensities. *Biochem Syst Ecol* 2013; **51**:264-268. doi: 10.1016/j.bse.2013.09.007.

968. MH Verdan, A Barison, EL de Sá *et al.* Lactones and quinones from the tubers of *Sinningia aggregata*. *J Nat Prod* 2010; **73**:1434–1437.

969. Pedersen JA. On the application of electron paramagnetic resonance in the study of naturally occurring quinones and quinols. *Spectrochimica Acta* 2002; **58**:1257-1270.

970. Thomson R. *Naturally occurring quinones.* New York: Butterworths Scientific Publications, 1957.

971. Thomson RH. Distribution of naturally occurring quinones. *Int J Clin Pharm* 1991; **13**(2):70-73. doi: 10.1007/BF01974983.

972. Usai M, Marchetti M. Anthraquinone distribution in the hypogeal apparatus of *Rubia peregrina* L. growing wild in Sardinia. *Natural Product Research,* 2010; **24**(7):626-632. doi: 10.1080/14786410902884842.

973. W Daugherty, S Smith, C Wigal *et al.* Distribution of 5-hydroxy-1,4-naphthoquinone and other napthoquinone derivatives in the Juglandaceae (Walnut Family) and Related Families. *10th Annual Penn State Symposium on Phytochemicals and Health, State Coll*. State College, PA, 1995.

974. Xiang-Hai Cai, Xiao-Dong Luo, Jun Zhou *et al.* Quinones from *Chirita eburnea*. *J Nat Prod* 2005; **68**:797-799.

975. Danuta Sobolewska, Klaudia Michalska, Irma Podolak *et al.* Steroidal saponins from the genus *Allium*. *Phytochemistry Reviews* 2016; **15**:1-35. doi: 10.1007/s11101-014-9381-1).

976. GF Pauli, JB Friesen, T Gödecke *et al.* Occurrence of progesterone and related animal steroids in two higher plants. *J Nat Prod* 2010; **73**(3):338-345.

977. L. Dinan, T. Savchenko, Whiting P. On the distribution of phytoecdysteroids in plants. *Cellular and Molecular Life Sciences* 2001; **58**:1121-1132.

978. Maria Renata de M. B. Borin, Gottlieb OR. Steroids, taxonomic markers? *Plant Systematics and Evolution* 1993; **184**(1):41–76. doi: 10.1007/bf00937778.

979. Y Schun, Cordell G. Cytotoxic steroids of *Gelsemium sempervirens*. *J Nat Prod* 1987; **50**(2):195-198.

980. Y Wang, K Ohtani, R Kasai *et al.* Steroidal saponins from fruits of *Tribulus terrestris*. *Phytochemistry* 1996; **42**(5):1417-1422.

981. Barbehenn, RV, Constabel CP. Tannins in plant-herbivore interactions. *Phytochemistry* 2011; **72**:1551-1565. doi: 10.1016/j.phytochem.2011.01.040.

982. Bate-Smith E, Metcalfe C. The nature and systematic distribution of tannins in dicotyledonous plants. *Bot J Linn Soc* 1957; **55**:669-705.

983. Colin M. Orians, Megan E. Gri$ths, Bernadette M. Roche *et al.* Phenolic glycosides and condensed tannins in *Salix sericea*, *S. eriocephala* and their F1 hybrids - not all hybrids are created equal. *Biochem Syst Ecol* 2000; **28**:619-632.

984. Haslam E. Vegetable tannins - lessons of a phytochemical lifetime. *Phytochemistry* 2007; **68**(22-24):2713-21. doi: 10.1016/j.phytochem.2007.09.009.

985. JF Stevens, H Hart, RCHJV Ham *et al.* Distribution of alkaloids and tannins in the Crassulaceae. *Biochemical Systematics & Ecology* 1995; **23**(2):157-165.

986. JM Koponen, AM Happonen, PH Mattila *et al.* Contents of anthocyanins and ellagitannins in selected foods consumed in Finland. *J Agr Food Chem*2007; **55**(4):1612-1619.

987. M König, E Scholz, R Hartmann *et al.* Ellagitannins and complex tannins from *Quercus petraea* Bark. *J Nat Prod* 1994; **57**(10):1411-1415.

988. MA Orabi, S Taniguchi, M Yoshimura *et al.* Hydrolyzable tannins of tamaricaceous plants. iii. hellinoyl- and macrocyclic-type ellagitannins from *Tamarix nilotica*. *J Nat Prod* 2010; **73**:870-879.

989. Moilanen J, Koskinen P, Salminen J-P. Distribution and content of ellagitannins in Finnish plant species. *Phytochemistry* 2015; **116**:188-197. doi: 10.1016/j.phytochem.2015.03.002.

990. Mole S. The systematic distribution of tannins in the leaves of angiosperms: A tool for ecological studies. *Biochemical Systematics & Ecology* 1993; **21**:833-846.

991. Petra Grundhofera, Ruth Niemetza, Gerhard Schillingb *et al.* Biosynthesis and subcellular distribution of hydrolyzable tannins. *Phytochemistr* 2001; **57**:915-927.

992. T Okuda, T Yoshida, Hatano T. Correlation of oxidative transformations of hydrolyzable tannins and plant evolution. *Phytochemistry* 2000; **55**:513-529.

993. ZH Jiang, XY Wen, T Tanaka *et al.* Cytotoxic hydrolyzable tannins from *Balanophora japonica*. *J Nat Prod* 2008; **71**(4):719-723.

994. AD Bakuridze, TD Dargaeva, GG Nikolaeva *et al.* Iridoids of plants of the genus *Gentiana* from the family Gentianaceae. *Chemistry of Natural Compounds* 1987; **27**:1-7.

995. Andrew RL, Keszei A, Foley WJ. Intensive sampling identifies previously unknown chemotypes, population divergence and biosynthetic connections among terpenoids in *Eucalyptus tricarpa*. *Phytochemistry* 2013; **94**:148-58. doi: 10.1016/j.phytochem.2013.05.002.

996. Carrier DJ, Beek TAv, Heijden Rvd *et al.* Distribution of ginkgolides and terpenoid biosynthetic activity in *Ginkgo biloba*. *Phytochemistry* 1998; **48**(1):89-92. doi: 10.1016/s0031-9422(97)00450-0.

997. Elodie A. Courtois, Christopher Baraloto, C.E. Timothy Paine *et al.* Differences in volatile terpene composition between the bark and leaves of tropical tree species. *Phytochemistry* 2012; **82**:81-88. doi: 10.1016/j.phytochem.2012.07.003.

998. Francesco Loreto, Francesca Bagnoli, Fineschi S. One species, many terpenes: matching chemical and biological diversity. *Trends in Plant Science* 2009; **14**(8):416-420. doi: 10.1016/j.tplants.2009.06.003.

999. G. H. Neil Towers, Stafford HA. *Recent Advances in Phytochemistry.* Springer-Verlag US: Springer US, 1990.

1000. Gilsane Lino von Poser, Jan Schripsema, Amelia T. Henriques *et al.* The distribution of iridoids in Bignoniaceae. *Biochem Syst Ecol* 2000; **28**:351-366.

1001. Kishan Gopal Ramawat, Mérillon J-M. *Natural Products: Phytochemistry, Botany and Metabolism of Alkaloids, Phenolics and Terpenes.* Springer-Verlag Berlin Heidelberg: Springer Berlin Heidelberg, 2013.

1002. LJ Elnaggar, Beal J. Iridoids. A Review. *J Nat Prod* 1980; **43**:649-707.

1003. M Guiso, C Marra, F Piccioni *et al.* Iridoid and phenylpropanoid glucosides from *Tecoma capensis*. *Phytochemistry* 1997; **45**:193-194.

1004. Amoa OP, Ntie-Kang F, Lifongo LL *et al.* The potential of anti-malarial compounds derived from African medicinal plants, part I: a pharmacological evaluation of alkaloids and terpenoids. *Malaria Journal* 2013; **12**(1):449-474.

1005. Harrewijn P, Minks A, Mollema C. Evolution of plant volatile production in insect-plant relationships. *Chemoecology* 1994; **5**(2):55-73. doi: 10.1007/BF01259434.

1006. R Julkunen-Tiitto, M Rousi, J Bryant *et al.* Chemical diversity of several Betulaceae species: comparison of phenolics and terpenoids in northern birch stems. *Trees* 1996; **11**:16-22.

1007. SC Joshi, RC Padalia, DS Bisht *et al.* Terpenoid diversity in the leaf essential oils of himalayan Lauraceae species. *Chemistry & Biodiversity* 2009; **6**:1364-1373.

1008. SSM Isabel, Kaplan M. Biosynthesis significance of iridoids in chemosystematics. *J Brazil Chem Soc* 2001; **12**(2):144-153.
